# Porphyrin‐Based Porous Materials for Antibacterial Applications

**DOI:** 10.1002/advs.78159

**Published:** 2026-10-06

**Authors:** Xiao‐Xue Liang, Xin Li, Ying Xiong, Xin Wang, Ying‐Wei Yang

**Affiliations:** ^1^ China‐Japan Union Hospital of Jilin University and College of Chemistry Jilin University Changchun People’ Republic of China; ^2^ College of Chemistry Liaoning University Shenyang People’ Republic of China

**Keywords:** antibacterials, photodynamic therapy, porous materials, porphyrins

## Abstract

Bacterial infections are serious threats to human life and health and constitute a major global health challenge. Although antibiotics effectively combat bacteria, the emergence of antibiotic resistance severely compromises their efficacy. Therefore, an efficient and practical approach is urgently sought to improve antibacterial efficiency. Porphyrins, as typical photosensitizers (PSs), serve as favorable candidates for constructing porous materials with enhanced properties due to their advantages, including easy modification, strong light‐absorbing ability, and good biocompatibility. Typically, porphyrin‐based porous materials are categorized into four classic types according to their linkage modes, including porphyrin‐based metal–organic frameworks (PMOFs), porphyrin‐based covalent organic frameworks (PCOFs), porphyrin‐based hydrogen‐bonded organic frameworks (PHOFs), and amorphous porphyrin‐based porous polymers (APPPs). This review summarizes antimicrobial advances in porphyrin‐based porous materials from the perspective of synthetic strategies and structural design in the past 10 years. Key areas include healthcare, food, agriculture, functional textiles, and wastewater treatment. Moreover, the challenges and future development directions in this field are discussed. This review inspires and motivates more researchers to focus on the innovative construction of porphyrin‐based porous materials to facilitate the efficient realization of antimicrobial applications.

## Graphical abstract

Porphyrin‐based porous materials have excellent photophysical properties and tunable structures for high‐efficiency antibacterial therapy. This review comprehensively discusses the structural design strategies and synthetic pathways of the four main types of porphyrin‐based porous materials, summarizes their cross‐domain antimicrobial applications, and prospects key challenges and future optimization directions.

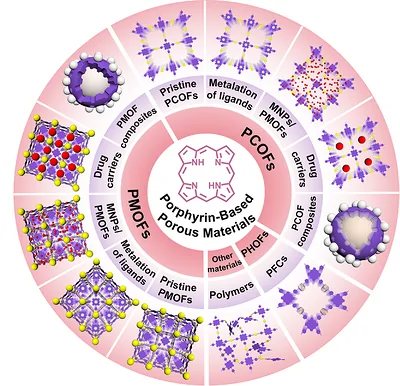

## 1 Introduction

Bacterial infections pose significant threats to human health and have led to a series of major global public health problems [1, 2]. Since the first antibiotic, penicillin, was used for antimicrobial therapy, countless lives have been saved [3]. Conventional antibiotics inhibit bacterial growth primarily by interfering with protein function, disrupting cell wall synthesis, and disrupting metabolic pathways. However, with the increasing use of antibiotics, bacteria gradually develop multidrug resistance through evolution [4]. This not only decreases the effectiveness of antibiotics but also leads to a rapid increase in the number of resistant bacterial species, posing an unprecedented challenge to human health. Therefore, the identification, development, and application of antimicrobial materials with better efficacy, lower usage dose, and higher biocompatibility are urgently needed [5].

Phototherapy is a non‐invasive therapeutic method that irradiates patients with low‐intensity light of specific wavelengths for the treatment of cancer, bacterial infection, and other diseases [6]. It is characterized by minimal risk of drug resistance and precise targeting, and can be categorized into photodynamic therapy (PDT) and photothermal therapy (PTT) according to different mechanisms [7]. PDT is a therapeutic means that generates reactive oxygen species (ROS) via photosensitizers (PSs), specific wavelengths of light, and oxygen (O_2_) [8]. PSs are excited to higher energy states upon light absorption, generating ROS through type I reaction (electron transfer) or type II reaction (energy transfer) pathways and producing singlet oxygen (^1^O_2_) and superoxide anions ($O_{2}^{\cdot -}$,). These ROS can simultaneously induce oxidative damage to bacterial membranes, proteins, and nucleic acids. This mechanism is fundamentally different from that of conventional antibiotics, which usually act on specific molecular targets. Consequently, common antibiotic resistance mechanisms, such as drug efflux, antibiotic‐inactivating enzymes, and target modification, are generally ineffective in protecting bacteria from the multi‐site oxidative damage induced by PDT [9, 10]. PTT ablates cells through the photothermal conversion effect of the photothermal agents [11, 12]. The principle is that light irradiates electrons in photothermal agents and releases energy through non‐radiation processes, increasing the local temperature to damage diseased tissues. Notably, porphyrins, as molecules with strong light absorption ability, have been widely studied as PSs and photothermal agents.

Porphyrins and their derivatives are widely distributed in nature, such as hemoglobin (in animals) and chlorophyll (in plants) [13]. Porphyrins, as conjugated macrocyclic molecules with 18 π electrons, exhibit broad light absorption across the UV and visible ranges [14]. Due to their excellent photophysical properties and high thermal and chemical stability, they are widely used in biomedicine and photo/electrocatalysis. However, due to strong π interactions among aromatic macrocycles, facile aggregation, poor solubility, and low photosensitization efficiency, the practical applications of porphyrin molecules are limited [14]. A possible solution is to develop porphyrin‐based porous materials to optimize the optical properties. Moreover, this strategy exploits porphyrin's intrinsic properties, achieving synergistic PDT, PTT, and other therapies, significantly overcoming the limitations of monotherapy.

Porous materials contain abundant pores ranging from micropores to macropores. They exhibit high specific surface areas, high absorption capacity, customizable structures, and excellent chemical stability. Because of their excellent biocompatibility and high loading efficiency, porous materials are extensively used in biomedical fields, including antimicrobial therapy [15], anticancer, and bioimaging [16]. Among them, porphyrin‐based porous materials have emerged as strong candidates for antibacterial applications due to their intrinsic photosensitivity. Typically, the emerging porphyrin‐based porous materials can be categorized as porphyrin‐based metal–organic frameworks (PMOFs), porphyrin‐based covalent organic frameworks (PCOFs), porphyrin‐based hydrogen‐bonded organic frameworks (PHOFs), and amorphous porphyrin‐based porous polymers (APPPs). Both the original and the post‐modified porphyrin‐based porous derivatives play important roles in the antimicrobial field. Therefore, the design, synthesis, and optimization of porphyrin‐based porous materials are of great importance.

Recently, many studies on porphyrin‐based materials applied in biomedicine have been reported. Some reviews focus on the specific types of porphyrin‐based materials, such as metal–organic frameworks (MOFs) and covalent organic frameworks (COFs), for biomedical applications exemplified by antimicrobial therapy [17, 18, 19, 20, 21, 22, 23, 24, 25, 26, 27]. Other reviews discuss phototherapy as an important therapeutic strategy in biomedicine [28, 29, 30, 31, 32]. However, comprehensive reviews specifically focusing on the antimicrobial applications of porphyrin‐based porous materials remain limited. To provide a systematic overview of this important area, this review summarizes the advantages and recent advances of porphyrin‐based porous materials in antimicrobial applications, including wound healing, [33, 34] food preservation, [35] osteomyelitis, [36, 37] periodontitis, [38] wastewater treatment, [39] and functional textiles. Additionally, special attention is focused on the construction strategies, block species, synthetic methods, and antibacterial mechanisms. Finally, we discuss the current challenges and future perspectives, including simplification of synthetic procedures, assessment of long‐term toxicity, elucidation of antimicrobial mechanisms, expansion of antibacterial spectrum, and design of new porphyrin‐based porous materials (Scheme 1).

**SCHEME 1:**
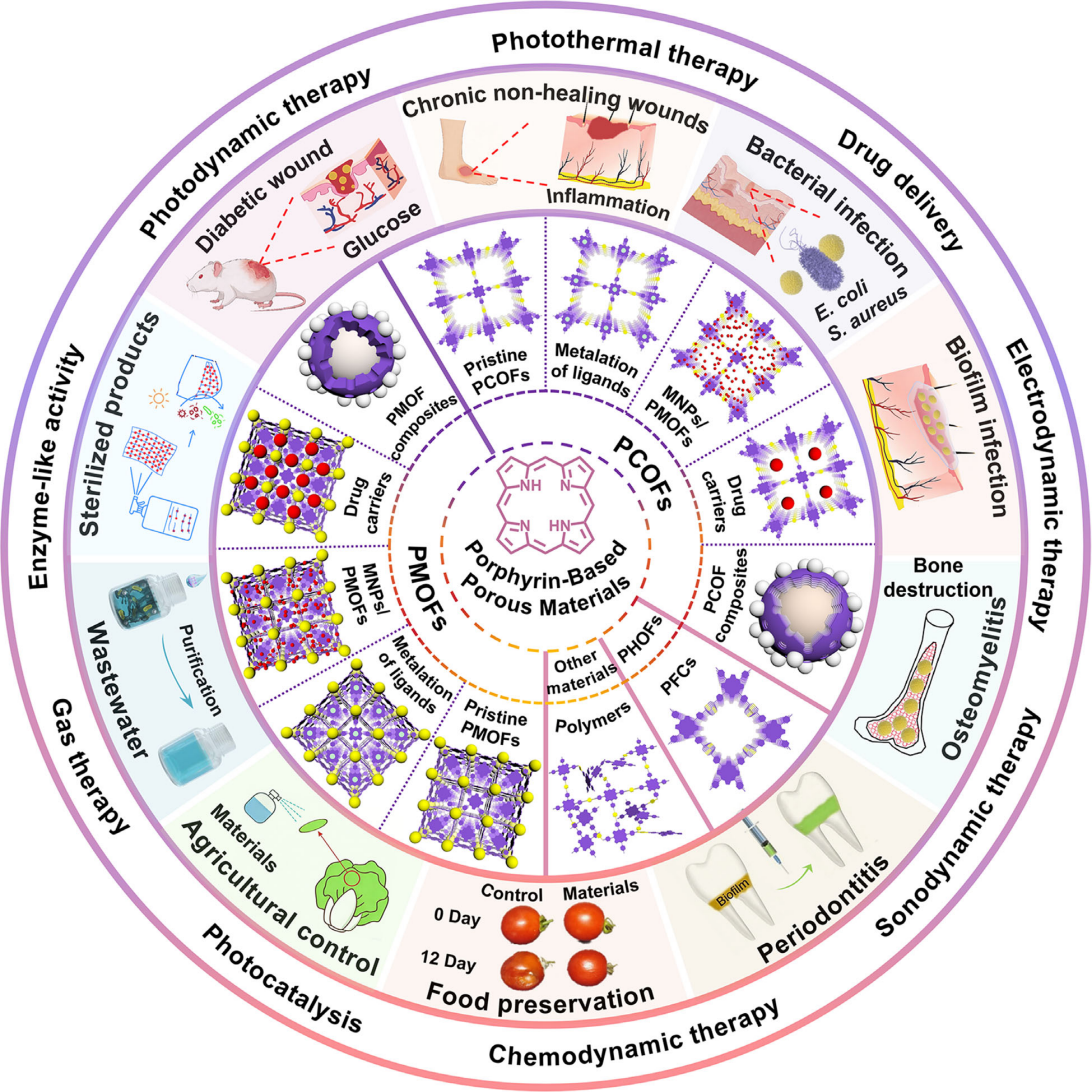
Schematic illustration of porphyrin‐based porous materials for antibacterial applications. Some elements were created with Autodesk 3ds Max and Microsoft PowerPoint.

## 2 The Construction Strategies of Porphyrin‐Based Porous Materials

This review systematically classifies porphyrin‐based porous materials into four major categories based on the different types of bonding in porous materials: PMOFs, PCOFs, PHOFs, and APPPs. MOFs are a class of periodic network structures consisting of metal ions or metal clusters linked with organic ligands via coordination bonds [40]. The original MOFs typically have a large specific surface area and adjustable structure. Since the first MOF‐5 was synthesized by Professor Omar M. Yaghi in 1999, [41] many kinds of representative MOFs such as Zeolitic Imidazolate Frameworks (ZIF), [42] University of Oslo (UiO), [43] Material Institute Lavoisier (MIL), [44] and Hong Kong University of Science and Technology (HKUST) [45] have been reported. PMOFs typically use 5,10,15,20‐tetrakis(4‐carboxyphenyl)porphyrin (TCPP) as a rigid core ligand to maintain a stable, porous framework. Various functional small molecules are introduced as auxiliary ligands to participate in the coordination assembly process. This regulates the pore structure and physicochemical properties of the PMOFs, achieving function‐directed modification.

COFs are a class of crystalline, porous polymeric materials constructed from organic monomers linked by covalent bonds. They feature highly ordered pore structures and tunable functional properties. In 2011, professor Omar M. Yaghi's team successfully synthesized two types of PCOFs for the first time: COF‐366 and COF‐66, featuring –C–N– and –B–O– linkages, respectively [46]. Since then, various functionalized PCOFs have been developed and applied in many different research fields. In the construction of PCOFs, porphyrin functional monomers typically serve as core skeletal units, and the porphyrin ring surface can be modified with various covalently reactive groups such as amino, aldehyde, and boronic acid groups. At the same time, different types of reversible covalent condensation reactions can be accommodated by introducing diverse functional groups into the bridging copolymer monomers, thereby enabling the directed synthesis of PCOFs with distinct topological structures and physicochemical properties.

Hydrogen‐bonded organic frameworks (HOFs) are three‐dimensional (3D), porous structures formed by the self‐assembly of organic or metal–organic building blocks via intermolecular hydrogen bonds (H‐bonds). Unlike MOFs and COFs, HOFs are formed through weak interactions rather than metal coordination or strong covalent bonds. Consequently, HOFs are less stable and more reversible than the other two types of framework materials. H‐bonds are usually formed between a hydrogen atom and a more electronegative atom X*^δ^* ^−^⋯H*^δ^* ^+^–Y*^δ^* ^−^ (X and Y being primarily N, O, and F), which are generally weakly binding, flexible, and reversible. Therefore, the hydrogen bond energy in most HOFs is intermediate between the van der Waals forces (< 10 kJ mol^−1^) and chemical bonds, typically 10–40 kJ mol^−1^ [47]. In addition to H‐bonds, other intermolecular forces such as electrostatic interactions, π‐π interactions, and van der Waals forces also play crucial roles in the structure of HOFs. The earliest study on HOFs was published in 1969 by David J. Duchamp, who prepared hexagonal hydrogen‐bonding networks using 1,3,5‐benzenetricarboxylic acid as ligands [48]. From then on, the development of HOFs fell into a dormant period until 2011, when Chen et al. applied them to the selective separation of C_2_H_4_/C_2_H_6_ [49]. Chen et al. also prepared the microporous HOF‐7 using ZnTDPP for the first time in 2015, with highly selective separation for CO_2_/N_2_, which also laid a strong basis for the study of PHOFs [50].

In comparison to the three crystalline porous materials mentioned above, APPPs are characterized by disordered architectures, irregular pore networks, and relatively low specific surface areas. Their X‐ray diffraction patterns exhibit only broad diffraction halos, directly verifying their amorphous nature. Despite these structural and pore‐performance limitations, this class of polymers possesses prominent advantages: facile synthetic procedures, easy scalability, excellent chemical and thermal stability, high loading capacity for functional porphyrin units, and versatile post‐synthesis modification potential. In summary, APPPs are structurally distinct from crystalline PMOFs, PCOFs, and PHOFs while complementing their functionalities, and thus have been extensively explored for bio‐related applications.

Here, we primarily summarize the construction strategies of porphyrin‐based porous materials and corresponding blocks, synthetic conditions, and antimicrobial mechanisms, aiming to support theoretical guidance and design ideas for synthesizing customized porous materials.

### 2.1 PMOFs

#### 2.1.1 Pristine PMOFs

PMOFs are widely explored and investigated as a kind of porous material in the biological field. In the past decades, diverse reasonable design strategies were used to build PMOFs. The construction strategies of PMOFs can be divided into pristine PMOFs, metalation of PMOFs, PMOFs as drug carriers, PMOFs/metal nanoparticles (MNPs) hybrids, and PMOF composites (Figure 1). The skeleton components, synthetic conditions, antibacterial mechanisms, and antibacterial categories of PMOF‐based antibacterial agents discussed in this review are summarized (Table 1). In general, PMOFs can be prepared directly via a simple one‐pot solvothermal method. Using this method, both metal salts and porphyrin ligands are mixed in an organic solvent, and crystals can be obtained after growing for a specific time. Various metals can be used in the synthesis of PMOFs. All metals show distinctive advantages, such as traditional Zr‐based PMOFs, which have high stability due to their high coordination numbers and strong coordination ability [51]. Cu/Fe‐based PMOFs usually exhibit peroxidase (POD)‐like activity, which can produce toxic hydroxyl radicals (·OH) [52, 53]. Ag‐based PMOFs have an inherent antibacterial ability through releasing Ag^+^ and improving photocatalytic activity [54]. Zn/Ti‐based PMOFs can also achieve enhanced photocatalysis [55, 56]. Sc‐based PMOFs can be used to improve the yield of ROS [57]. Bi‐based PMOFs can disrupt the redox homeostasis of bacteria via strong interactions between Bi^3+^ and H_2_S, thus synergistically boosting the efficacy of sonodynamic therapy (SDT) [58].

**FIGURE 1:**
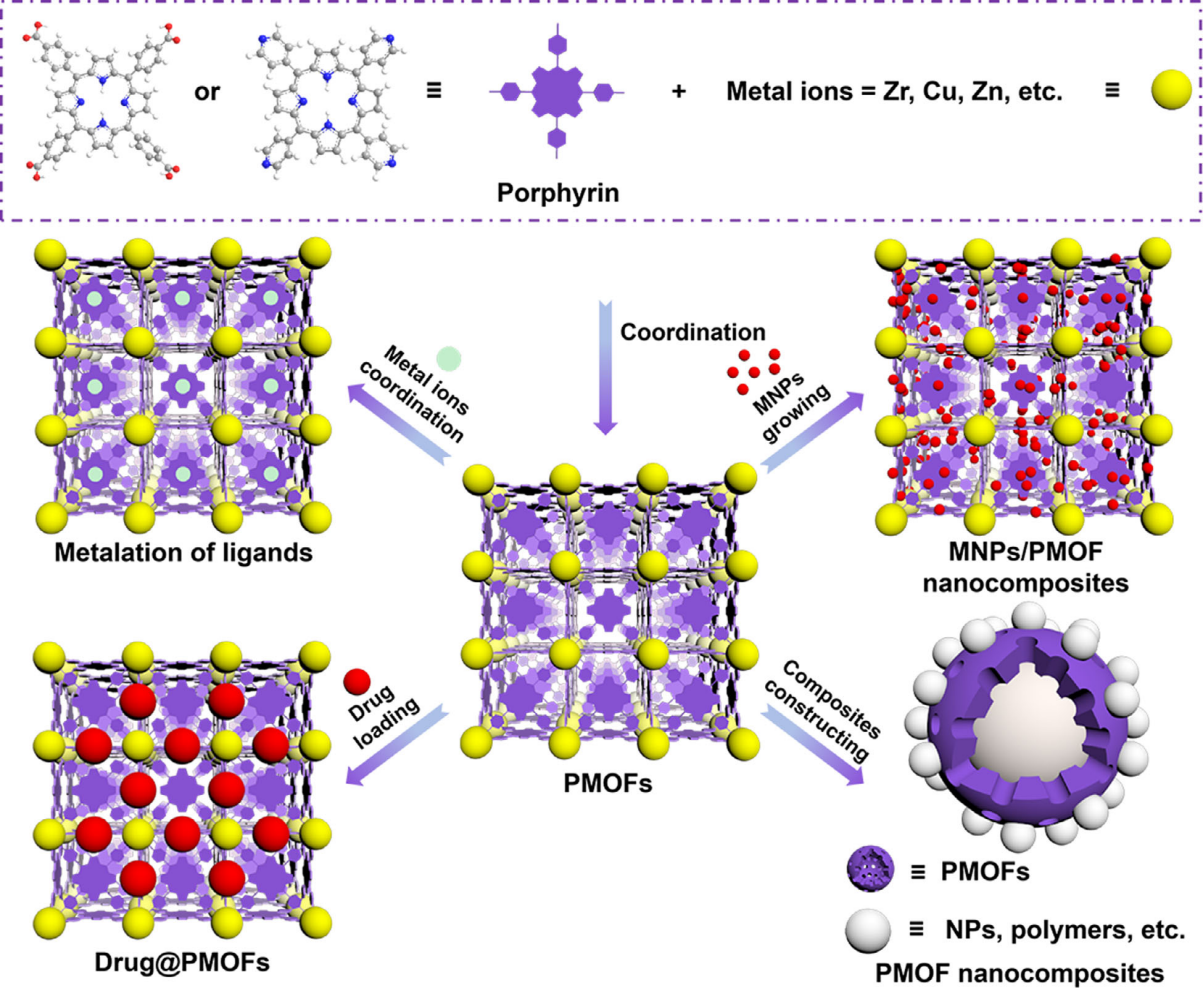
The construction strategies of PMOF‐based antibacterial agents. Created with Autodesk 3ds Max and Microsoft PowerPoint.

**TABLE 1:** Typical examples of PMOF‐based antimicrobial agents in this review.

PMOF‐Based Antibacterial Agents	PMOFs Skeleton Components	Reaction Conditions of PMOFs (v/v)	Bacteria	Antibacterial Mechanisms	Ref.
2D ZnTCPP	Zn^2+^, TCPP	DMF/EtOH (12/5), 80°C, 16 h	*S. aureus* and *P. aeruginosa*	PDT	[63]
3DNDITcpp	Zn^2+^, TCPP, NDI	DEF/H_2_O (3/1), 85°C, 48 h	*S. aureus* and *E. coli*	PDT	[62]
Zn‐TCPP	Zn^2+^, TCPP	DMF (27), 150°C, 1 h	*S. aureus*	PDT	[56]
Nano‐PCN‐222(Fe)	Zr^4+^, FeTCPP	DMF/H_2_O/FA (6/0.1/0.48), 120°C, 24 h	*S. aureus* and *E. coli*	Nanozyme activity	[53]
Bi NPs/PCN‐224	Zr^4+^, TCPP, BA	DMF (50), 120°C, 1 h	*S. aureus*, *E. coli*, and MRSA	PDT/Bi^3+^ release	[85]
M‐TG	Zr^4+^, TCPP, AG	DMF/HOAc (18/1.6), 90°C, 18 h	*S. aureus* and *E. coli*	PDT/AG release	[61]
UiO‐66(SH)_2_@TCPP@AgNPs	Zr^4+^, TCPP, TPA	DMF (2), 130°C, 12 h	*S. aureus*, *E. coli*, and MRSA	PDT/Ag NPs enhanced ROS	[82]
ZPM@Ag	Zr^4+^, TCPP	DMF/H_2_O (2/0.2), 120°C, 24 h	*S. aureus* and *E. coli*	PDT/Ag NPs enhanced ROS	[83]
Ag‐CuTCPP MOFs	Cu^2+^, TCPP	DMF/EtOH/H_2_O (3/3/3), 85°C, 48 h	*S. aureus*, *E. coli*, and *B. subtilis*	Ag^+^ release	[86]
Au NCs@PCN	Zr^4+^, TCPP	DMF (5), 100°C, 12 h	*S. aureus*, *E. coli*, MRSA, and *AmpR E. coli*	CDT/PDT/PTT	[87]
CuTCPP@AgNPs@MBA	Cu^2+^, TCPP	DMF/EtOH/TFA (60/20/0.05), 80°C, 3 h	*S. aureus*	PDT/Ag^+^ release/Target	[81]
PCMX	Zr^4+^, TCPP	DMF (24), 120°C, 24 h	*S. aureus*	SDT	[84]
UCNPs@ZrMOF‐Pt	Zr^4+^, TCPP	DMF/EtOH (12/4), 120°C, 5 h	*S. aureus* and *E. coli*	PDT/Nanozyme activity	[88]
PB@MOF	Zr^4+^, TCPP, TPA	DMF (9), 90°C, 2.5 h	*S. aureus* and *E. coli*	PDT/PTT	[89]
PCN‐224@CeO_2_	Zr^4+^, TCPP	DMF (100), 90°C, 5 h	*S. aureus*	PDT	[79]
Zr‐TCPP@PDA@FP	Zr^4+^, TCPP, NH_2_‐BDC	DMF (15), 130°C, 12 h	*S. aureus* and *E. coli*	PTT	[72]
SZN‐MOFs	Cu^2+^, Zn‐TCPP	DMF/EtOH/TFA (12/4/0.04), 80°C, 4 h	*S. aureus*	CDT	[52]
Ag_9_‐AgTPyP	Ag, TPyP	DMF/CHCl_3_ (6/1), 25°C	*S. aureus*, *E. coli*, MRSA, and *P. aeruginosa*	Photocatalysis	[90]
S‐PCN‐134	Zr^4+^, TCPP, HOAc, BTB	DMF (7), 120°C	*S. aureus*	PDT	[91]
D‐PCN‐2	Zr^4+^, TCPP	DMF/H_2_O (8/0.8), 120°C, 24 h	MRSA	SDT	[92]
TCPPCu‐BBDC	Zr^4+^, TCPPCu, BBDC	DMF/FA (10/0.05), 130°C, 12 h	*S. aureus*, *E. coli*, MRSA, and MDR *E. coli*	PDT/Target	[93]
M/DP MN patch	Zr^4+^, TCPP	DMF (90), 90°C, 5 h	*S. aureus*, *E. coli*, and *P. aeruginosa*	CDT/PDT/DMOG and MEM release	[94]
HN25	Zr^4+^, TCPP, ALN	DMF/H_2_O (2/0.2), 120°C, 24 h	MRSA	SDT/ALN release	[95]
THY@PCN/PUL/PVA	Zr^4+^, TCPP	DMF (250), 90°C, 5 h	*S. aureus*, *E. coli*, and *B. cinerea*	PDT/THY release	[96]
PC‐GR@MOF‐545	Zr^4+^, TCPP	DMF (250), 90°C, 5 h	*S. aureus*, *E. coli*, and MRSA	PDT/GR release	[35]
PCN‐CO@PVP	Zr^4+^, TCPP	DMF/CHCl_2_COOH (260/5), 120°C, 24 h	*S. aureus*, *E. coli*, and MRSA	PDT/GT	[97]
Cu_10_MOFs	Zr^4+^, Cu^2+^, TCPP	DMF (8), 120°C, 24 h	*S. aureus*, *E. coli*, and MRSA	PDT/PTT	[98]
PCN‐224(Zr/Ti)	Zr^4+^, TCPP	DMF (10), 120°C, 1 h	*E. coli*, MDR *E. coli, A. baumannii*, MDR *A. baumannii, S. aureus*, MRSA, and *S. epidermidis*	PDT	[55]
PCN‐222‐Pt	Zr^4+^, TCPP, BA	DMF/H_2_O (8/0.8), 120°C, 24 h	*S. aureus* and *E. coli*	Nanozyme activity	[38]
LL‐37‐HA‐Au‐Pt@PCN‐224	Zr^4+^, TCPP	DMF/HOAc (8/0.5), 120°C, 24 h	*S. aureus* and *E. coli*	PDT/Nanozyme activity	[99]
Ag@UT	Zr^4+^, TCPP, TPA	DMF (10), 130°C, 12 h	*S. aureus*	SDT/Ag^+^ release	[100]
Pt@PCN‐222	Zr^4+^, TCPP	DMF (50), 90°C, 5 h	*P. gingivalis*, *A. actinomycetemcomitans*, and *S. mutans*	PDT/Nanozyme activity	[101]
UCNP@PCN@LA‐PVDF	Zr^4+^, TCPP	DMF (50), 90°C, 5 h	*S. aureus* and *P. aeruginosa*	PDT/GT	[102]
CuTCPP@MOF@Mannosamine	Cu^2+^, TCPP	DMF (15), 90°C, 24 h	*S. aureus* and *E. coli*	SDT/CDT/Target	[103]
PB‐PCN‐224	Zr^4+^, TCPP	DMF (10), 90°C, 5 h	*S. aureus*	PDT/PTT	[104]
Ga/Cu‐TCPP@PAMAM	Cu^2+^, TCPP	DMF(20), 80°C, 24 h	*S. aureus* and *E. coli*	PDT/PTT/INIT	[105]
Cu‐TCPP	Cu^2+^, TCPP	DMF/EtOH/TFA (60/20/0.08), 80°C, 4 h	*S. aureus* and *E. coli*	CDT	[106]
MOF‐PDA	Zr^4+^, TCPP	DMF 120°C, 24 h	*S. aureus* and *E. coli*	PDT/PTT	[51]
PCN@PDA	Zr^4+^, TCPP	DMF (2), 120°C, 24 h	*S. aureus* and *E. coli*	PDT/PTT	[107]
MMNPs	Hf^4+^, TCPP	DMF/HOAc (10/0.4), 80°C, 24 h	*S. aureus* and *E. coli*	PDT/Cascade catalysis/Target	[108]
UMOF(Pt)/IR808	Hf^4+^, Pt‐TCPP	DMF/EtOH (0.75/0.75), 100°C, 7 h	MRSA	PDT/Cascade catalysis/Target	[109]
MCGC hydrogel	Zr^4+^, TCPP	DMF (100), 90°C, 5 h	*S. aureus* and *E. coli*	CGA/Nanozyme activity	[110]
MSN@MOF/SNP NPs	Ti^4+^, TCPP	DMF/HOAc (1/0.0005), 80°C, 24 h	MRSA	PDT/GT	[111]
2DMOF‐TiO_2_	Zn^2+^, TCPP	DMF/EtOH (48/16), 80°C, 24 h	MRSA	SDT	[112]
MOF@EV	Zr^4+^, TCPP	DMF/H_2_O (15/1), 120°C, 8 h	*S. aureus* and MRSA	EDT	[113]
B‐HA@Zn‐TCPP	Zn^2+^, TCPP	DMF (50), 150°C, 80 min	*S. aureus*, *E. coli*, and MRSA	SDT/Target	[114]
Cu‐TCPP@MnO_2_	Cu^2+^, TCPP	DMF/EtOH (15/5), 80°C, 24 h	*S. aureus* and *E. coli*	CDT/PDT/PTT/Cascade catalysis	[115]
PCN‐222‐BTO	Zr^4+^, TCPP	H_2_O/DMF (1.2/12), 120°C, 24 h	*E. coli* and MRSA	SDT	[116]
RBC‐HNTM‐Pt@Au	Zr^4+^, TCPP	H_2_O/DMF (0.2/2), 120°C, 24 h	MRSA	SDT/Pt SAs and Au NRs enhanced ROS	[36]
Cur/CuPP‐PELA	Cu^2+^, TCPP	DMF/H_2_O (20/1), 130°C, 12 h	*P. aeruginosa*	PTT/Cur release	[117]
Cu‐TCPP@DIN@MPN	Cu^2+^, TCPP	DMF (‐)	*F. oxysporum*	DIN/Cu^2+^ release	[118]
PCN@Ir‐QCS	Zr^4+^, TCPP, BA	DMF (100), 90°C, 5 h	*S. aureus* and *E. coli*	PDT/CDT/Nanozyme activity	[119]
Mn@PCN‐224	Zr^4+^, TCPP, BA	DMF (100), 90°C, 5 h	*S. aureus* and *E. coli*	PTT/Nanozyme activity	[120]
Ag@MOF‐545	Zr^4+^, TCPP	DMF/H_2_O (‐), 120°C, 8 h	*S. aureus*, *E. coli*, and MRSA	PDT/Ag^+^ release/Target	[121]
PCN‐222(Mn)‐Cur	Zr^4+^, TCPP, BA	DMF (40), 90°C, 5 h	MRSA	SDT/Nanozyme activity/Cur release	[122]
Mn_0.1_PCC	Zr^4+^, Mn^2+^, TCPP	DMF (24), 120°C, 24 h	MRSA	CDT/Cascade catalysis	[77]
SAGD‐ScMPFs	Sc^3+^, TCPP	DMF/H_2_O (17/3), 60°C, 2 h, 100°C, 2 h	*S. aureus*, *E. coli*, and *Salmonella*	Sc^3+^ enhanced ROS	[57]
Bi_2_S_3_/Cu‐TCPP	Cu^2+^, TCPP	DMF (20), 80°C, 24 h	*P. gingivalis*	PDT/PTT/CDT	[123]

The (v/v) represents the volume ratio of different solvents. Abbreviations: Tetrakis(4‐carboxyphenyl)porphyrin, TCPP; 2,7‐di(pyridin‐4‐yl)benzo[lmn] [3, 8] phenanthroline‐1,3,6,8(2H,7H)‐tetraone, NDI; Benzoic acid, BA; 1,4‐dicarboxybenzene, TPA; 2‐aminobenzene‐1,4‐dicarboxylic acid, NH_2_‐BDC; 5,10,15,20‐tetraphenylporphyrin, TPyP; 1,3,5‐tris(4‐carboxyphenyl)benzene, BTB; 2‐borono‐1,4‐benzenedicarboxylic acid, BBDC; Alendronate, ALN N,N‐dimethylformamide, DMF; Ethyl alcohol, EtOH; N,N‐diethylformamide, DEF; Formic acid, FA; Acetic acid, HOAc; Trifluoroacetic acid, TFA; Trichloromethane, CHCl_3_; *Staphylococcus aureus, S. aureus*; *Pseudomonas aeruginosa, P. aeruginosa*; *Escherichia coli, E. coli*; Methicillin‐resistant *Staphylococcus aureus*, MRSA; *Bacillus subtilis, B. subtilis*; *Ampicillin‐resistant Escherichia coli*, AmpR *E. coli*; Multidrug‐resistant *Escherichia coli*, MDR *E. coli*; *Botrytis cinerea, B. cinerea*; *Acinetobacter baumannii, A. baumannii*; Multidrug‐resistant *Acinetobacter baumannii*, MDR *A. baumannii*; *Staphylococcus epidermidis, S. epidermidis*; *Porphyromonas gingivalis, P. gingivalis*; *Aggregatibacter actinomycetemcomitans, A. actinomycetemcomitans*; *Streptococcus mutans, S. mutans*; *Fusarium oxysporum, F. oxysporum*; Dimethyloxalylglycine, DMOG; Meropenem, MEM; Thymol, THY; Geraniol, GR; Chlorogenic acid, CGA; Single atoms, SAs; Nanorods, NRs; Curcumin, Cur; Diniconazole, DIN.

Pristine PMOFs are characterized by simple synthetic methods, high yields, and easy scale‐up synthesis, which are reasons why they are widely developed in the antimicrobial field. Moreover, different linking groups, such as pyridine and carboxyl groups, can be used to construct PMOFs. For example, in collaboration with Tian et al. in 2021, we reported a hollow structure of Zr(IV) MOF (H‐PMOF) through a one‐pot method using TCPP and ZrCl_4_. Due to its unique hollow structure, H‐PMOF can be a carrier to load indocyanine green and achieve enhanced photodynamic effects [59]. Young et al. prepared a 3D Cu‐TMPP using meso‐tetrakis(6‐methylpyridin‐3‐yl)porphyrin and a [Cu_8_Cl_6_]^10+^ cluster. To apply it to biological applications, Cu‐TMPP was modified with polyvinylpyrrolidone (PVP), which exhibited high POD‐like activity and photodynamic effects [60].

In addition to synthesizing PMOFs using single porphyrin ligands, strategies employing dual ligands have also been applied. In fact, when one ligand is a porphyrin, additional ligands can be introduced as functional groups, such as targeting moieties, antibacterial drugs, and electron donors/acceptors. This not only enriches the structural blueprints of PMOFs but also improves their antibacterial properties. For example, based on ammonium glycyrrhizinate (AG), which exhibits antibacterial and antioxidant properties, Zr clusters show abundant coordination sites. Xie et al. introduced AG as a ligand into Zr‐based PMOFs (M‐TG). This approach not only regulated the structures of PMOFs but also achieved a long‐term antibacterial effect of AG and increased ^1^O_2_ production [61]. Moreover, Zhu et al. introduced electron acceptor 2,7‐di(pyridin‐4‐yl)benzo[lmn] [3,8]phenanthroline‐1,3,6,8(2H,7H)‐tetraone (NDI) into PMOFs, realizing the transformation of layered 2DTcpp to pillar‐layered 3DNDITcpp. Remarkably, these D‐A PMOFs exhibited effective photogenerated electron transfer interactions and materialized superior photocatalytic ROS generation [62].

Some surfactants, such as PVP, are also added to the PMOFs to modulate the morphology and characteristics of the materials. In the preparation of PMOFs, surfactants can selectively attach to the surface of PMOFs to modify their anisotropic growth. For example, Boyer et al. synthesized 2D ZnTCPP nanosheets by the selective adsorption of PVP on crystalline surfaces, where PVP played the role of tuning topography, improving stability, and enhancing photocatalytic performance. Owing to its excellent heterogeneous photocatalysis and mechanical properties, ZnTCPP is used for high‐precision 3D printing and antibacterial applications [63].

#### 2.1.2 Metalation of PMOFs

The metalation strategies for PMOFs mainly include coordination of metal ions, which can be divided into pre‐ and post‐synthetic metalation of ligands. Pre‐synthetic metalation of ligands introduces metal ion coordination into porphyrin ligands and synthesizes PMOFs using them as ligands. Post‐synthetic metalation of ligands mainly depends on the high surface area of PMOFs and porphyrin macrocycles complexed with the metal. They can endow multi‐functional properties such as enzyme‐like activity, biocompatibility, and photocatalytic activity, and acquire materials that pre‐metalation failed to synthesize.

Metalation of the porphyrin cavity is a key strategy for modulating type I/type II photodynamic pathways. These two pathways generally operate concurrently, and their relative contributions depend on the electronic structure of the porphyrin, the nature of the triplet state, substrate and O_2_ concentrations, aggregation state, and the local microenvironment [64]. For example, filled d^10^ electron configuration of Zn(II) causes little paramagnetic quenching and generally favors type II process [65]. However, metalation effects cannot be predicted solely from electronic configuration. The heavy‐atom effect of Pd(II) enhances intersystem crossing (ISC) and may promote either type I electron transfer or type II ^1^O_2_ generation [65]. In some frameworks, Fe(III)/Fe(II) and Mn(III)/Mn(II) redox cycling can promote type I electron transfer and the formation of radical ROS, whereas the paramagnetic Fe(III) and Mn(III) centers may accelerate nonradiative deactivation and thereby suppress type II ^1^O_2_ generation [66, 67]. Some Cu‐containing porous photocatalysts generate both ^1^O_2_ and $O_{2}^{\cdot -}$, further demonstrating the coexistence of type I and type II pathways [68].

#### 2.1.3 PMOFs Combination With Other Materials

PMOFs are effective strategies that can achieve multi‐functionality and high stability through incorporating other functional materials into PMOFs. Currently, various materials such as polymers, nanozymes, MNPs, metal oxides, and magnetic nanoparticles (NPs) are used for the design and construction of PMOFs. Compared with pristine PMOFs, their preparation is more complex because they involve the hybridization of two or more materials. Their synthetic strategies are mainly classified into core‐shell assembly, template growth, pore infusion/loading, and chemical bonding.

In the construction of the core‐shell structure, the core structure is the basis, and the heterogeneous materials are uniformly coated on its surface through chemical bonds and other interactions. PMOFs can be used as both “yolk” (core) and “egg white” (shell). However, they are often used as shells because PMOFs effectively protect the core materials while providing porous transport channels that can promote the stability and functionality of the composites. For instance, Fang et al. prepared nanocomposites with dopacid‐modified scintillator NPs as the core and Hf‐TCPP MOF as the shell, both of which are driven by coordination interactions between Hf^4+^ and carboxyl groups. The core exhibits strong green light emission, which can activate porphyrin to produce an amount of ROS. The core‐shell structure shows effective energy transfer, and the porous structure facilitates the dispersion of ROS. The Gd, Lu, and Hf of the scintillator also promote the production of ·OH [69].

The template growth strategy of PMOF composites involves a mixing solution of template and precursor solution of PMOFs, and growing PMOFs in the template by self‐assembly or the solvothermal method. Specifically, the topography and size of PMOFs depend on the template, so that we can acquire the specific topography. Depending on this method, various composite membranes are prepared for antibacterial applications [70, 71]. In this year, Duan et al. fabricated a cellulose paper‐based membrane, further modified it using polydopamine (PDA) and Zr‐TCPP MOF (Zr‐TCPP@PDA@FP), which could achieve a good photothermal antibacterial effect [72].

Connecting strategy involving PMOFs combining with other materials (including hydrogels, [73] electrostatically spun fibers, [74, 75] targeted structures, [76] and inorganic materials modification [77]) through chemical bonds (such as coordination bonds, covalent bonds) or physical mixing has emerged as an effective way to improve stability, biocompatibility, and functionality. Zhang et al. synthesized PCN@AgI heterojunction via electrostatic adsorption and in situ deposition to improve photocatalytic ability [78]. Ren et al. designed a PCN‐224@CeO_2_ through electrostatic adsorption and in situ reduction to limit extracellular adenosine triphosphate to disrupt the adhesion of bacteria [79]. Furthermore, MNPs‐modified PMOFs involve metal ions filling their pores or adsorbing on their surfaces through various interactions, followed by reduction to form MNPs, which also belongs to the connecting strategy. It can be divided into MNPs coated on PMOFs and MNPs encapsulated in PMOFs. The methods of MNPs coated on PMOFs mainly include chemical reduction, photoreduction, and thermal reduction. First, the chemical reduction method involves the reduction of metal ions through reduction agents (such as NaBH_4_, sodium citrate). It generally consists of three main steps: stages of nucleation of metal atoms, gradual polymerization to MNPs, and growth in situ on the surface or inside of PMOFs [80]. This method is usually performed at a low temperature, which is the most widely used. Ag NPs, due to the release of Ag^+^, produce ROS, and the targeted bacterial ability is applied for antibacterial applications. In 2022, Yang et al. grew Ag NPs in situ on the surface of CuTCPP MOF using NaBH_4_ reduction, exhibiting the integration and synergism of near‐infrared (NIR)‐induced Ag^+^ release and generation of ROS (CuTCPP@AgNPs@MBA) [81]. Photoreduction usually irradiates PSs with light, and metal ions are reduced by generating active substances, such as free radicals. For example, Lin et al. grew Ag NPs on a TCPP‐doped UiO‐66 composite through UV light irradiation to notably improve photocatalytic antibacterial ability [82]. Wu et al. reduced Pt NPs on the PCN‐222 using UV (365 nm), which could achieve POD‐like, oxidase‐like activities, and ROS production [38]. Remarkably, they also used thermal reduction to generate Pt single‐atom (SA)‐doped PCN‐222. In the thermal reduction method, metal ions are reduced through heating and homogeneously distributed on the surface and inside of PMOFs. Qiu et al. used thermal reduction to prepare Ag NPs‐doped Zr‐TCPP MOFs (ZPM@Ag), which exhibited synergistic, effective, and stable photocatalytic properties [83]. The perfusion/dispersion strategy is to homogeneously disperse PMOFs into other materials or disperse them into the pores of PMOFs to realize the construction of heterogeneous structures. To illustrate, Song et al. perfused the ultrasound‐activated catalyst Ti_3_C_2_T_x_ into PCN‐224 to devise composites (PCMX), which could be used for treating bacterial infectious osteomyelitis. Under activating by ultrasound, Ti_3_C_2_T_x_ induces electrons to the surface of the composites to contact O_2_, which promotes the generation of ROS [84].

### 2.2 PCOFs

#### 2.2.1 Pristine PCOFs

According to the different building blocks of linkages, the design of PCOFs is mainly based on two covalent bonding types: –C–N– and –B–O–, which can be attributed to their inherent reversible condensation properties. PCOFs based on the above two linkages were all fabricated by Omar M. Yaghi in 2011, which inspired the interest of researchers in the synthesis of PCOFs [46]. Generally, –C–N– linked PCOFs, a class of classic Schiff base products, feature favorable biocompatibility and strong resistance to water and acid. Compared with them, PCOFs based on –B–O– linkages show poor stability in water, acid, and nucleophilic reagents because of their intrinsically electron‐deficient boron, while they exhibit excellent chemical/thermal stability [24]. During the condensation process of PCOFs, those with the lowest energy, highest stability, and crystallinity are formed by constantly combining and reorganizing between porphyrins and blocks, which also highlights their structural adaptability and self‐healing properties. The synthetic conditions of PCOFs are also influenced by various factors, including the reaction time or temperature, solvent conditions, amount of acid, and the creation of a degassing environment.

The construction strategies of PCOFs can be divided into pristine PCOFs, PCOFs as drug carriers, and PCOF composites. In the antimicrobial field, PCOFs are primarily designed via diverse ligands condensing with porphyrins. Currently, many functional blocks, such as benzene, anthracene, pyrene, and their derivatives, are used for the construction of PCOFs [20]. Since the properties of PCOFs can be optimized using different building blocks with porphyrin condensation, we summarize the structures of various porphyrin ligands (Figure 2a) and organic blocks (Figure 2b) for the construction of PCOFs. We also summarize the building units, synthesis conditions, antibacterial mechanisms, and types of PCOF‐based antibacterial agents mentioned in this review (Table 2). The different construction strategies of PCOF‐based antimicrobial agents are presented in Figure 3. Therefore, we hope to provide design insights for researchers, and more novel and customized PCOFs are reported by outlining the different blocks.

**FIGURE 2:**
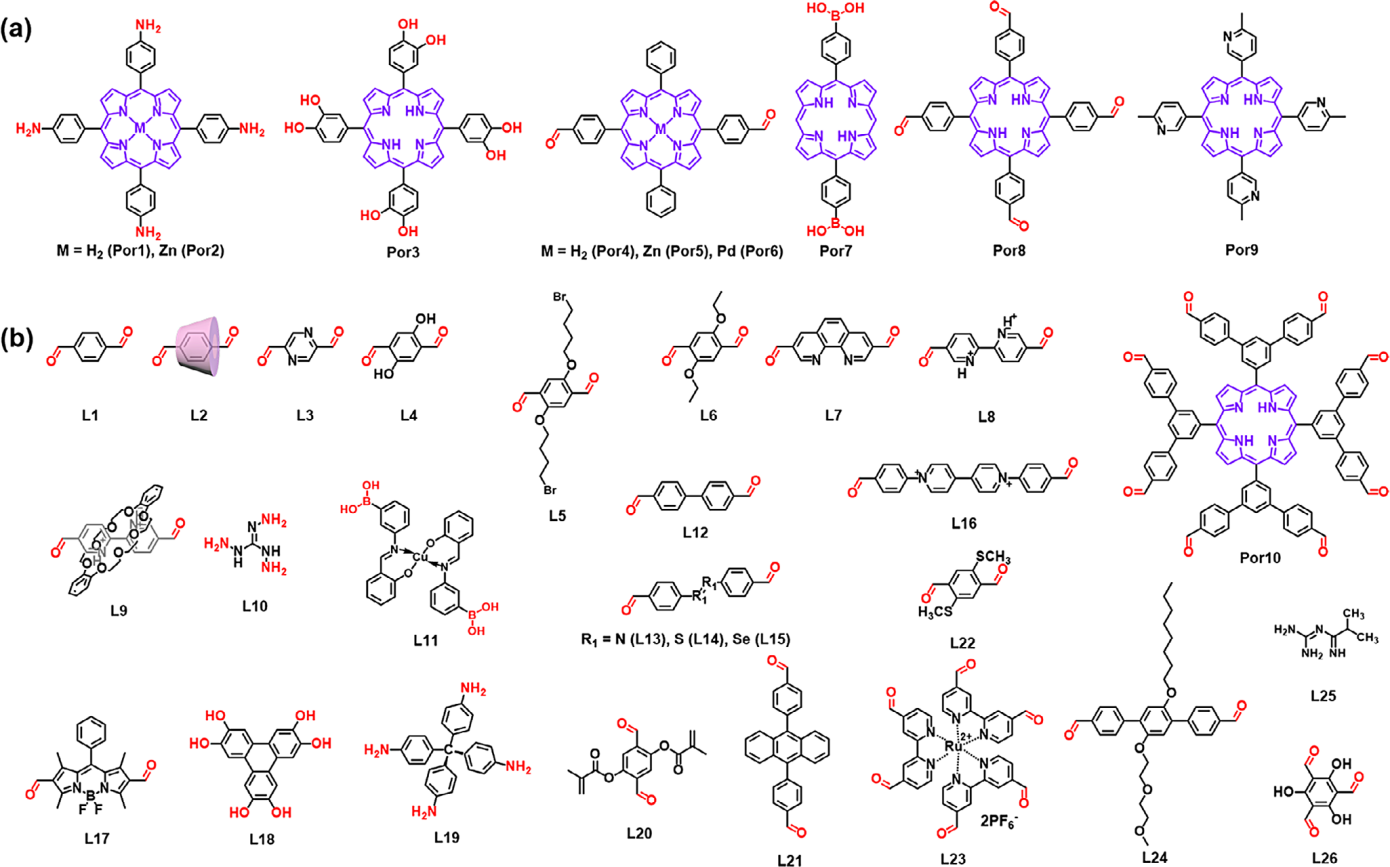
The construction of PCOFs. (a) Typical structures of various porphyrin ligands for the construction of PCOFs. (b) Typical examples of organic blocks for the synthesis of PCOFs. Chemical structures were drawn using ChemDraw.

**TABLE 2:** All discussed PCOFs are summarized in this review, with all monomer structures shown in Figure 2.

PCOF‐Based Antibacterial Agents	Building Units of PCOFs	Reaction Conditions of PCOFs (v/v)	Bacteria	Antibacterial Mechanisms	Ref.
3D‐TPP (M = H_2_, Pd)	Por4 + L19 Por6 + L19	1,4‐Dioxane/3 M HOAc (3.5/0.2), 120°C, 72 h	*P. aeruginosa* and *E. faecalis*	PDT	[124]
2D‐TPP	Por1 + L19	EtOH/Mesitylene (2/2/0.4), 120°C, 72 h			
COF‐366 DhaTph JNU‐2	Por1 + L1 Por1 + L4 Por1 + L6	*n*‐BuOH/Mesitylene/6 M HOAc (1/1/0.1), 120°C, 72 h *o*‐DCB/*n*‐BuOH/6 M HOAc (1/1/0.1), 120°C, 72 h Mesitylene/6 M HOAc (2/0.2), 120°C, 72 h	*S. aureus* and *E. coli*	PDT	[125]
Por‐DPA COF	Por1 + L21	Mesitylene/*n*‐BuOH/6 M HOAc (1.8/0.2/0.2), 120°C, 72 h	*S. aureus* and *E. coli*	PDT/PTT	[126]
Crown‐COPF‐Zn Por‐POP	Por1 + L8 + L9 Por1 + L8	1,4‐Dioxane/Mesitylene/6 M HOAc (3/3/0.1), 60°C, 72 h	*S. aureus* and *E. coli*	PDT/PTT/Zn^2+^ release	[127]
BPTG_Cl_ COF	Por5 + L10	DMF/H_2_O (2/0.25), 110°C, 72 h	*S. aureus* and *E. faecalis*	PDT/Target	[128]
HRCOFs	Por1 + L13	THF/HF (9/0.2), 25°C, 12 h	*S. aureus* and *E. coli*	PDT/PTT	[129]
Tph‐BDP‐COF	Por1 + L17	*n*‐BuOH/Mesitylene/6 M HOAc (1/1/0.25), 120°C, 72 h	*E. coli*	PDT/PTT	[130]
Por‐COF Por‐CD‐COF	Por1 + L1 Por1 + L2	1,4‐Dioxane/Mesitylene/6 M HOAc (3/3/1), 120°C, 72 h	*S. aureus* and *E. coli*	PDT/PTT	[131]
TP‐Por CON@BNN6	Por7 + L18	CH_3_CN/Mesitylene (0.7/0.3)	*S. aureus* and *E. coli*	PDT/PTT/GT	[132]
IBU@DhaTph	Por1 + L1	DCM/H_2_O/HOAc (30/48/1), 25°C, 168 h	*S. aureus* and *E. coli*	PDT/IBU release	[133]
DEG‐HEP‐COF@Lev&Van	Por1 + L24	EtOH/6 M HOAc (1.0/0.2), 120°C, 72 h	MRSA	Lev and Van release	[134]
Ag@ZnPor‐COFs	Por2 + L4	*o*‐DCB/*n*‐BuOH/3 M HOAc (1/1/0.2), 120°C, 72 h	*S. aureus* and *E. coli*	PDT/PTT	[135]
Ag@DhaTph‐COOH	Por1 + L4 + Pyr	*o*‐DCB/*n*‐BuOH/6 M HOAc (2/2/0.2), 120°C, 72 h	*S. aureus* and *E. coli*	PDT/PTT/Ag NPs enhanced ROS	[136]
HA‐Fe‐COF	Por1 + L3	1,4‐Dioxane/Mesitylene/6 M HOAc (1/1), 120°C, 72 h	*S. aureus*	PDT/PTT/CDT	[137]
DAC/Por‐COPs	Por2 + L26	*o*‐DCB/*n*‐BuOH/15 M HOAc (1/1/0.2), 120°C, 72 h	*S. aureus* and *E. coli*	PDT	[138]
Por‐COF‐Gel	Por1 + L20	*o*‐DCB/*n*‐BuOH/15 M HOAc (0.2/1.8/0.2), 120°C, 72 h	*S. aureus* and *E. coli*	PDT/PTT/Target	[139]
COF_Zn_@HPTA	Por1 + L14 Por1 + L15	1,4‐Dioxane/Mesitylene/6 M HOAc (1/1/0.6), 120°C, 72 h	*S. aureus* and *E. coli*	PTT/Zn^2+^ release/GOx‐like activity	[140]
Polypor (Os)	Por1 + Por8	*o*‐DCB/*n*‐BuOH/6 M HOAc (0.5/0.5/0.1), 120°C, 72 h	MRSA	SDT/Nanozyme activity	[141]
TAPP‐VIO	Por1 + L16	*n*‐BuOH/Mesitylene/6 M HOAc (1/1/0.2), 120°C, 144 h	*S. aureus* and *E. coli*	PDT/PTT/Target	[142]
CuSA‐COF	Por1 + L7	1,4‐Dioxane/CH_3_CN/6 M HOAc (0.5/0.5/0.1), 120°C, 72 h	*S. aureus, E. coli*, and MRSA	PDT/Ferroptosis/ Nanozyme activity	[143]
DmtaTph	Por1 + L22	Mesitylene/6 M HOAc (2/0.2), 120°C, 72 h	*S. aureus* and *E. coli*	PDT	[39]
CRuP‐COF	Por9 + L23 + BA	EtBr (2/0.2), 180°C, 180 h	*S. aureus* and *E. coli*	PDT/PTT	[144]
Cu‐B‐COF	Por3 + L11	CH_3_CN/1,4‐Dioxane/Mesitylene (60/12/3), 90°C, 18 h	*S. aureus* and *E. coli*	PDT/PTT/Nanozyme activity	[145]
MT‐LYZ	TCPP + L25	H_2_O, 180°C, 11 h	MRSA	PDT/PTT	[146]
Zn‐COF‐NHG	Por2 + L4 + L5	*o*‐DCB/*n*‐BuOH/3 M HOAc (0.5/0.5/0.1), 120°C, 72 h	*S. aureus* and *E. coli*	PDT/Nanozyme activity	[147]
P‐COF	Por9 + Por10	1,4‐Dioxane/Mesitylene/TFA (2.5/2.5/1), 140°C, 120 h	*S. aureus* and *E. coli*	PDT/PTT/Nanozyme activity	[148]

The (v/v) represents the volume ratio of different solvents. Abbreviations: Pyruvic acid, Pyr; Benzoic anhydride, BA; *n*‐butanol, *n*‐BuOH; 1,2‐dichlorobenzene, *o*‐DCB; Tetrahydrofuran, THF; Hydrogen fluoride, HF; Acetonitrile, CH_3_CN; Dichloromethane, DCM; Ethidium bromide, EtBr; *Pseudomonas aeruginosa, P. aeruginosa; Enterococcus faecalis, E. faecalis; Staphylococcus aureus, S. aureus; Escherichia coli, E. coli; Methicillin‐resistant Staphylococcus aureus*, MRSA; Ibuprofen, IBU; Levofloxacin, Lev; Vancomycin, Van; Glucose oxidase, GOx.

**FIGURE 3:**
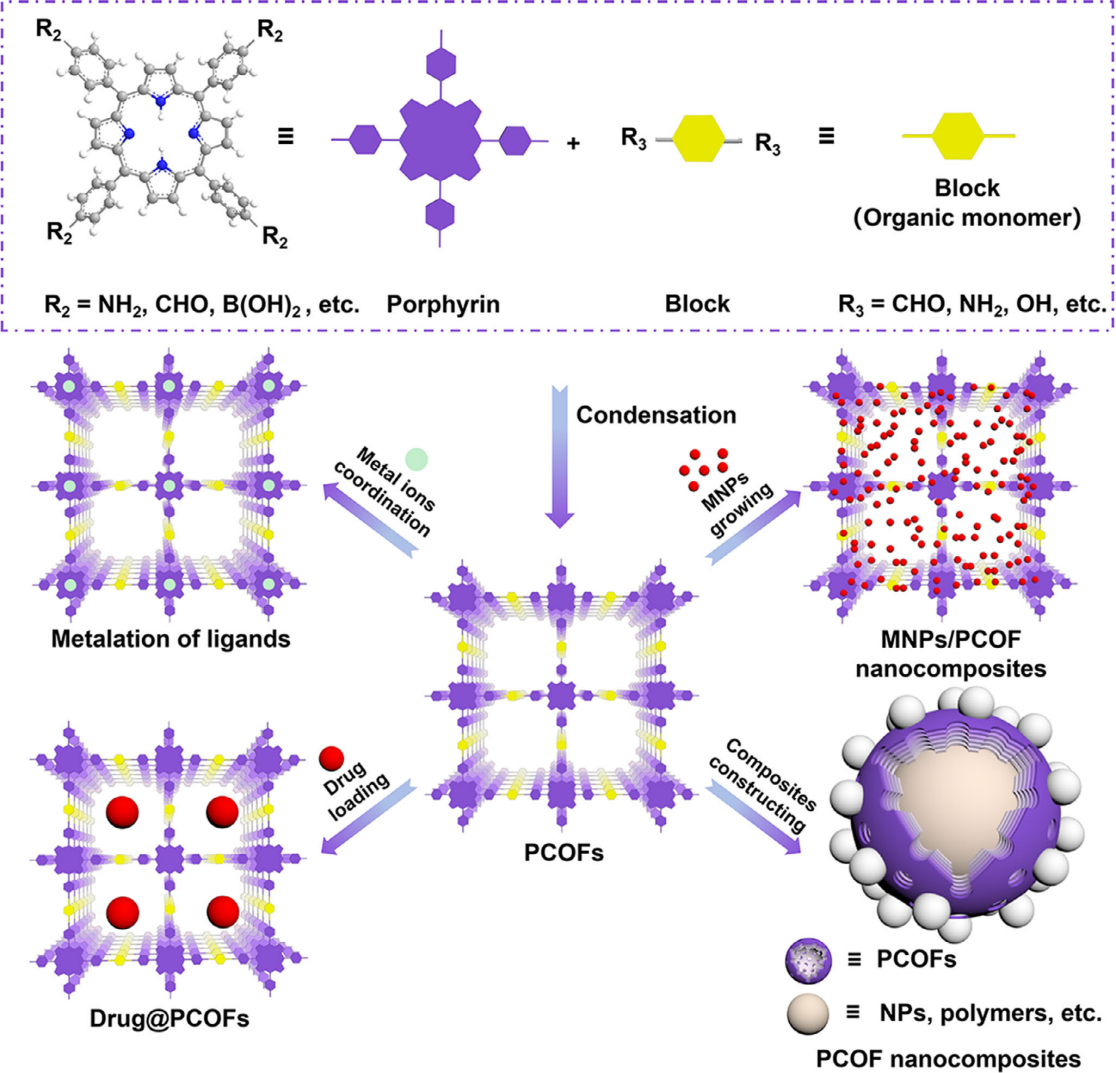
The construction strategies of PCOFs and PCOF‐based antimicrobial agents. Created with ChemDraw, Autodesk 3ds Max, and Microsoft PowerPoint.

#### 2.2.2 Metalation of PCOFs

Although PCOFs are usually characterized by high biocompatibility because of without metal component, metalation of PCOFs is an effective way of achieving synergistic antibacterial activity. The primary antimicrobial features of various metal ions are mentioned in “2.1.1.” and will not be repeated here. Metalation strategies of PCOFs can be achieved by ligands coordinating with metal ions. For example, Chen et al. loaded Cu^2+^ with Fenton‐like activity into black phosphorus‐doped Ga@COF to generate more heat and ROS [149]. Xiong et al. also used Fe^2+^ to synthesize HA‐Fe‐COF [137]. The two PCOFs achieved tri‐modal synergistic antibacterial effects, including photothermal, photodynamic, and chemodynamic therapy (CDT).

#### 2.2.3 PCOFs Combination With Other Materials

At present, PCOF composites mainly focus on pristine PCOFs, metalation of PCOFs, and PCOFs as carriers, but some articles report PCOF hydrogels [147], electrospinning [150], and other materials. The specific design strategy is mentioned in 2.1.3. and will not be repeated here.

### 2.3 PHOFs

Although the PHOFs have made much progress, their development is still in its infancy. The synthesis of both post‐modified and composite materials is limited, so we primarily elaborate on the construction of stabilized PHOFs. Table 3 summarizes some PHOFs mentioned in this review. The classifications of synthetic strategies for stable HOFs are as follows: (1) π‐π stacking, (2) highly interpenetrated networks, (3) chemically cross‐linked HOFs, and (4) charge‐assisted H‐bonds (Figure 4).

**TABLE 3:** Comprehensive overview of PHOFs for antibacterial applications.

PHOF‐based Antibacterial Agents	Building Units of PHOFs	Reaction Conditions of PHOFs (v/v)	Bacteria	Antibacterial Mechanisms	Ref.
JZS‐1	H_6_TZPP	DMF/H_2_O/CH_3_CN (4/2/2), 110°C, 72 h	*S. aureus*	PDT	[151]
PFC‐33	TCPP	MeOH/DMA/2 M TPA (5/0.5/0.07), 25°C, 48 h	*S. aureus* and *E. coli*	PDT/QA release	[152]
PFC‐73	Ni‐TCPP	DMF/TCB (4/8), 100°C, 24 h	*S. aureus* and *E. coli*	PDT/Ni^2+^ release	[153]
PFC‐73‐Ni	Ni‐TCPP	DMF (5), 100°C, 24 h	*E. coli*	PDT/PTT	[154]

The (v/v) represents the volume ratio of different solvents. Abbreviations: 5,10,15,20‐tetrakis[4‐(2,3,4,5tetrazolylphenyl)]porphyrin, H_6_TZPP; Methanol, MeOH; N,N‐dimethylacetamide, DMA; 1,2,4‐trichlorobenzene, TCB; *Staphylococcus aureus, S. aureus; Escherichia coli, E. coli;* Quaternary ammonium, QA.

**FIGURE 4:**
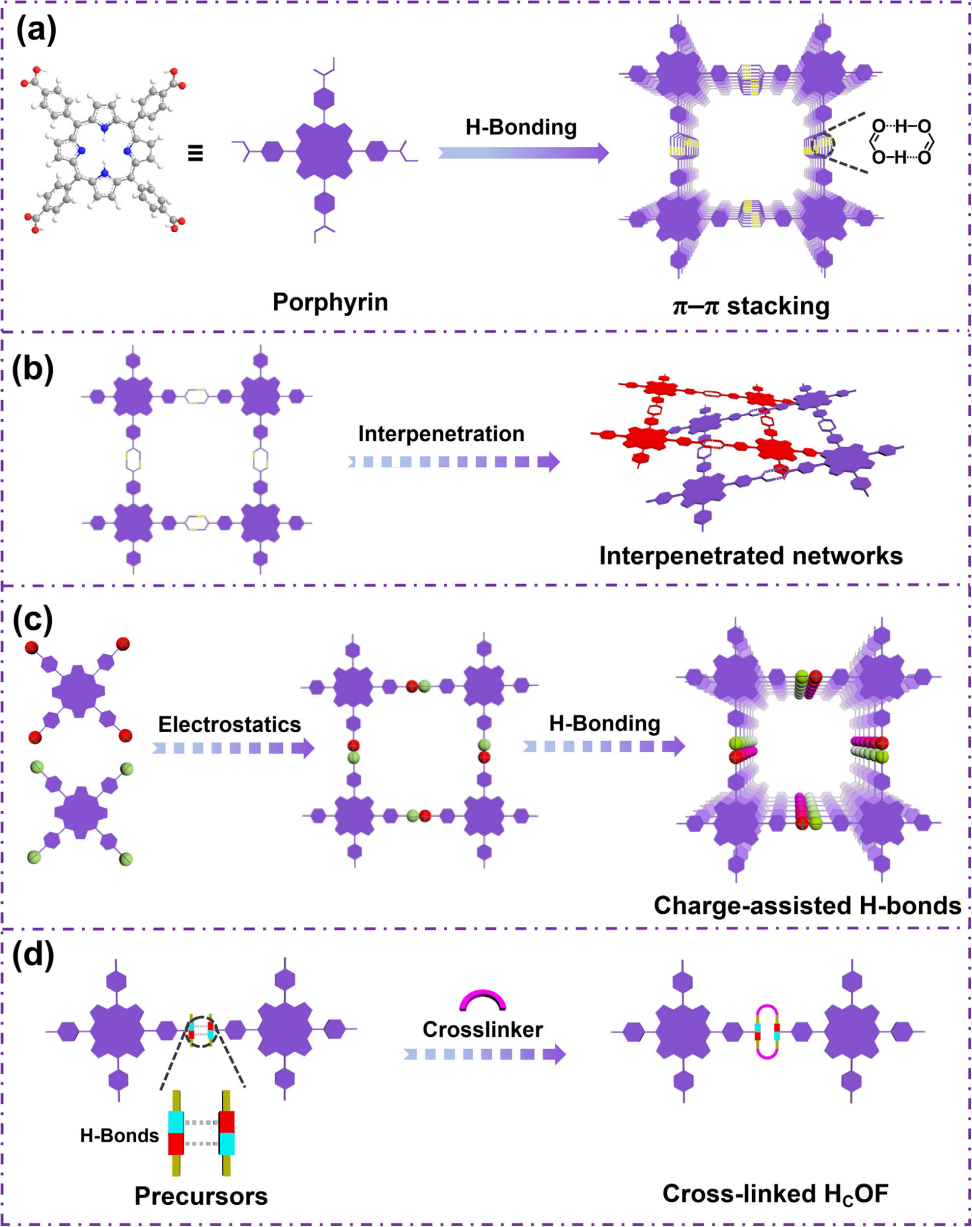
The construction strategies of PHOFs [158]. (a) The π‐π stacking interaction between porphyrin molecules forms a conjugated system with enhanced stability (resistant to organic solvents, acids, and bases). (b) By adjusting the reactant concentration and using template or epitaxial strategies, interpenetrating HOFs with adjustable pore environments and flexible structures can be constructed. (c) Two porphyrin molecules with opposite charges attract each other through electrostatic interaction and further form H‐bonds between layers. This strategy also helps to improve the stability of HOFs. (d) Two porphyrin molecules form preorganized and chemically cross‐linked molecular precursors through H‐bonding interactions, and further form hydrogen‐bonded cross‐linked organic frameworks (H_C_OFs) through crosslinker coupling. Created with ChemDraw, Autodesk 3ds Max, and Microsoft PowerPoint.

The π‐π stacking is a weak non‐covalent bond interaction that arises between aromatic rings (Figure 4a). In general, appropriate π‐π stacking can improve the stability of HOFs. Porphyrins, as planar conjugated macrocyclic molecules with 18 π electrons and multiple aromatic rings, can be used as organic building blocks for HOFs to form structures with stronger π‐π interactions. Highly interpenetrated networks also confer excellent stability to HOFs (Figure 4b). The cross‐adaptation of ligand structures can modulate the pore structure of HOFs to produce more interesting and novel structures. Hisaki et al. built a highly interpenetrated HOF using hexaazatriphenylene as a ligand, which possessed a high surface area of 1288 m^2^ g^−1^, heat resistance of 305°C, and strong acid stability of HCl [155]. However, PHOFs with highly interpenetrated networks have not been reported. In addition, charge‐assisted H‐bonds promote the formation of stabilized HOFs depending on charge interaction between two ionic building blocks with opposite electrical properties (Figure 4c) [156]. Wang et al. devised a nanoscale HOF through the charge‐assisted H‐bonding interaction of azobenzenedicarboxylate and tetrakis(4‐amidiniumphenyl)methane. Thanks to their opposite charges, the strength of H‐bonds is promoted, which maintains a stable structure at 300°C [155]. At last, Ke et al. fabricated a hydrogen‐bonded cross‐linked organic framework, in which the precursor molecules crystallized by H‐bonds, further achieving enhanced stability (Figure 4d). Remarkably, this method does not influence structural crystallization [157].

### 2.4 APPPs

Unlike PMOFs, PCOFs, and PHOFs, which possess crystalline and periodic frameworks, APPPs lack long‐range structural order. Although some synthetic approaches, such as condensation reactions, may resemble those used to prepare crystalline frameworks, the resulting materials are non‐crystalline and lack a defined topology. The construction strategies of APPPs can be categorized into in situ and condensation synthesis. In situ synthesis (Figure 5a). In general, APPPs can be directly polymerized by pyrrole (Py) and aldehyde‐modified monomers. This method is usually easy, but it cannot afford well‐crystallized and structurally ordered materials. The reason may be that Py polymerizes easily under light, so it produces some by‐products that will influence structural order. Currently, there are many works preparing APPPs using this method and apply in biological fields. Various functional monomers are used as the blocks, such as benzene, [159] ferrocene, [160] crown ether, [161] and its derivatives [162]. In 2019, we also synthesized two conjugated macrocyclic polymers using Py and dibenzaldehyde‐functionalized pillar [5]arene. After further loading, small‐sized Pd NPs can be used for the reduction of nitrophenol and catalyze Suzuki‐Miyaura coupling reactions [163]. Condensation synthesis (Figure 5b) [164]. APPPs can be constructed by condensation between porphyrin and the second ligand. The detailed construction strategies are mentioned in 2.2.1. and will not be repeated here. Some literature has also reported APPPs synthesized via terminal alkyne coupling reactions, which similarly exhibit potent antibacterial effects [165, 166].

**FIGURE 5:**
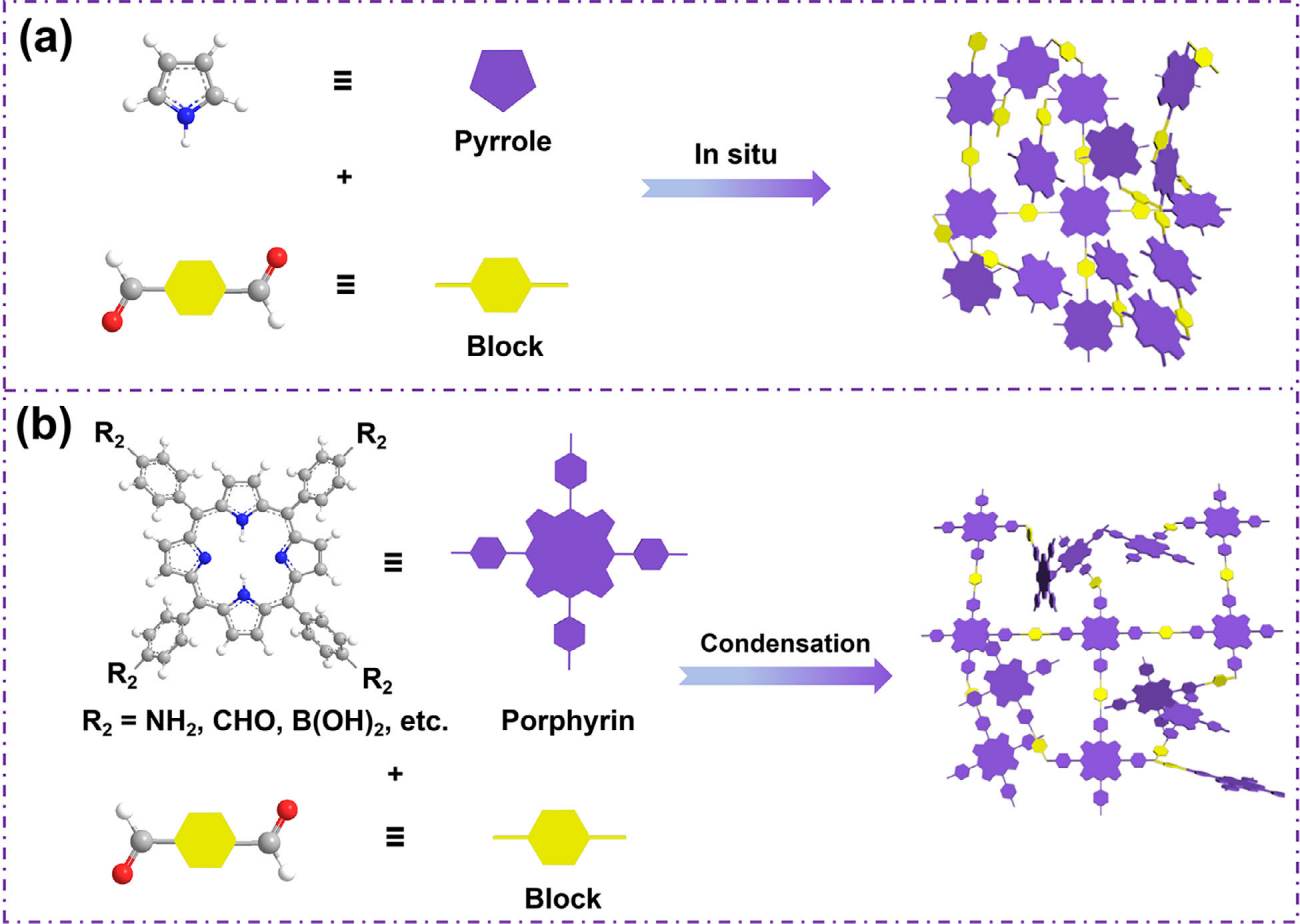
The construction strategies of APPPs. (a) Py and block are directly used to construct polymers in situ. (b) Porphyrin and block form polymers through aldimine condensation. Created with ChemDraw, Autodesk 3ds Max, and Microsoft PowerPoint.

## 3 The Antibacterial Applications of Porphyrin‐Based Porous Materials

Porphyrins, as macrocyclic molecules with strong light‐absorption abilities, have unique advantages in the antibacterial field [86]. Currently, porphyrin‐based porous materials focus on photodynamic, photothermal, and sonodynamic antibacterial strategies [167]. Moreover, based on their porous structure, they can be integrated with other materials (drugs, nanozymes, metal oxides, NPs, and polymers) to achieve synergistic or cascade antibacterial effects. In summary, these four material classes possess distinct and complementary structural and functional advantages (Table 4). PMOFs possess well‐ordered porous frameworks through metal–ligand coordination, which can prevent photosensitizer aggregation, facilitate mass transport, and promote charge transfer. However, their stability highly depends on the metal‐ligand combination. PCOFs immobilize porphyrins within robust covalent networks, exhibiting ordered channels and high structural stability [168]. However, their photodynamic properties are sensitive to linkage chemistry and interlayer stacking [24]. PHOFs are assembled mainly through reversible hydrogen bonds and other non‐covalent interactions under mild conditions, generally exhibiting lower stability in complex aqueous or biological environments. APPPs lack long‐range order and well‐defined pore channels, but provide advantages such as simple synthesis, high porphyrin loading, good chemical stability, and flexible functionalization. Although their bonding modes establish the structural basis for antibacterial performance, their overall antibacterial activity also depends on particle size, pore topology, metalation state, surface chemistry, and irradiation conditions. Therefore, a unified quantitative comparison among the four material categories is not appropriate.

**TABLE 4:** Comparative structural characteristics, advantages, and limitations of four major classes of porphyrin‐based porous materials.

Material Class	Bonding Mode	Framework Composition	Structural Order	Framework Stability	Advantages	Limitations	Ref.
PMOFs	Metal–ligand coordination bonds	▪ Metal ions/clusters + porphyrinic ligands ▪ Metal nodes are integral components of the framework	Highly ordered and crystalline	▪ Moderate to high ▪ Strongly metal–ligand dependent	▪ High structural tunability ▪ Intrinsic metal functionality ▪ Well‐defined porosity ▪ Broad modification space	▪ Possible hydrolytic instability ▪ Metal‐ion‐related biosafety	[35, 36, 53, 54, 84, 87, 88, 91, 92, 93, 94, 98, 99, 105, 108, 109, 110, 113, 117, 119, 120, 171, 172].
PCOFs	Covalent bonds	▪ Organic blocks ▪ No metal nodes in framework	Highly ordered and crystalline	▪ High ▪ Depends on linkage chemistry	▪ High chemical stability ▪ Ordered pore structure ▪ Metal‐free framework design ▪ High structural tunability	▪ Relatively complex and time‐consuming synthesis ▪ Potentially limited biodegradability	[125, 126, 127, 128, 129, 131, 133, 134, 137, 139, 140, 141, 142, 143, 144, 147, 180].
PHOFs	H‐bonds as primary interaction	▪ Organic blocks ▪ No metal nodes in framework	Ordered and crystalline	▪ Moderate ▪ More reversible and sensitive to solvent, pH, ionic strength	▪ Mild assembly conditions, reversibility ▪ Good solution processability	▪ Comparatively weak structural stability ▪ Susceptibility to disassembly during functionalization or in complex media	[151, 152, 153, 154]
APPPs	Covalent amorphous networks	▪ Organic blocks ▪ No metal nodes in framework	Amorphous	▪ Generally high	▪ Simple synthesis ▪ Mild preparation conditions ▪ Easy scale‐up ▪ High porphyrin loading ▪ Flexible functionalization	▪ Low structural order and irregular porosity ▪ Uncertain long‐term biodegradation	[161, 181, 182]

In fact, research on porphyrin‐based porous materials in the antimicrobial field is currently at a critical stage. Their applications span numerous sectors, including healthcare, food and agriculture, and wastewater treatment‒all of which are closely intertwined with our production activities and daily lives. This chapter aims to focus on the representative advances of porphyrin‐based porous materials in the antimicrobial field, and to motivate researchers to expand the biological antibacterial properties of porphyrins.

### 3.1 Antimicrobial Applications in Healthcare

In the field of healthcare, porphyrin‐based porous materials have been extensively applied in therapeutic research for various diseases due to their unique porous structures. From a structural design perspective, the porous characteristics of these materials not only confer high specific surface areas, significantly enhancing contact efficiency between the material and diseased tissues, but also serve as excellent carriers for drug loading and controlled release, thereby substantially improving therapeutic outcomes. Analyzing their chemical composition, the inherent building blocks of these porous materials also play a pivotal role in antimicrobial processes. For instance, metal ions, functional organic ligands, and photosensitive porphyrin groups can synergistically or independently exert antimicrobial efficacy through their respective mechanisms of action.

#### 3.1.1 Wound Healing

Diabetes is a highly prevalent metabolic disorder worldwide, characterized by persistent hyperglycemia [169]. In diabetic patients, wound healing is severely impaired by the complex pathological microenvironment, including hypoxia, high glucose levels, excessive oxidative stress, and abnormal local pH [170]. As a result, diabetic wounds readily deteriorate into chronic non‐healing wounds, significantly increasing the risk of amputation and mortality. Chronic wounds are commonly defined as wounds that fail to achieve obvious healing progress after 1 month of standard treatment, featuring complicated etiology, long inflammatory duration, and substantial clinical challenges. Notably, pathogenic bacteria tend to aggregate and form biofilms in most chronic wounds. Such biofilms exhibit strong resistance to conventional antibiotics and frequently cause persistent bacterial infections, eventually trapping wounds in a vicious cycle of “inflammation, infection, and non‐healing.” Therefore, the development of novel antimicrobial platforms with precise targeting, high bactericidal efficacy, and low propensity for drug resistance has become an urgent demand at the interface of materials science and medicine. Recently, porphyrin‐based porous materials have gained extensive attention in antimicrobial research owing to their tunable light absorption, large specific surface area, and highly functionalizable pore structures. Their adjustable photo‐response enables compatibility with diverse noninvasive therapeutic modalities, such as PDT, PTT, and SDT. Meanwhile, their high surface area and well‐defined pore architecture allow efficient loading and sustained release of antimicrobial agents, as well as the construction of multi‐site synergistic systems. Furthermore, chemical modification can introduce targeting moieties and nanozyme active centers, which further improve the targeting accuracy and synergistic antimicrobial efficacy. Accordingly, porphyrin‐based porous materials provide a promising strategy for the treatment of infectious wounds, biofilm‐associated infections, and diabetic chronic wounds.

*PMOFs Promote Wound Healing*: Pristine PMOFs feature simple synthesis, higher biocompatibility, and easy degradation, compared with PMOF nanocomposites. Various functional ligands and metal centers have currently been incorporated into the structural design of PMOFs, giving the materials diverse functional properties [114]. In 2021, Yan et al. reported the implementation of enhanced photodynamic abilities by changing the topology of the PMOFs. In this study, four PMOFs, including MOF‐525, MOF‐545, PCN‐223, and PCN‐224 with **ftw**, **csq**, **shp**, and **she** topologies, were synthesized, respectively (Figure 6a). By comparing their topologies, the researchers attribute the generation of ^1^O_2_ to the size of the pores and distances between the TCPP active sites. In contrast, for the two similar PMOFs mentioned above, it is mainly determined by the amount of TCPP molecules in each Zr_6_O_8_ cluster. Furthermore, the researchers also verify this conclusion through antibacterial experiments. This work lays the foundation for the design of new PMOFs with photodynamic properties [171].

**FIGURE 6:**
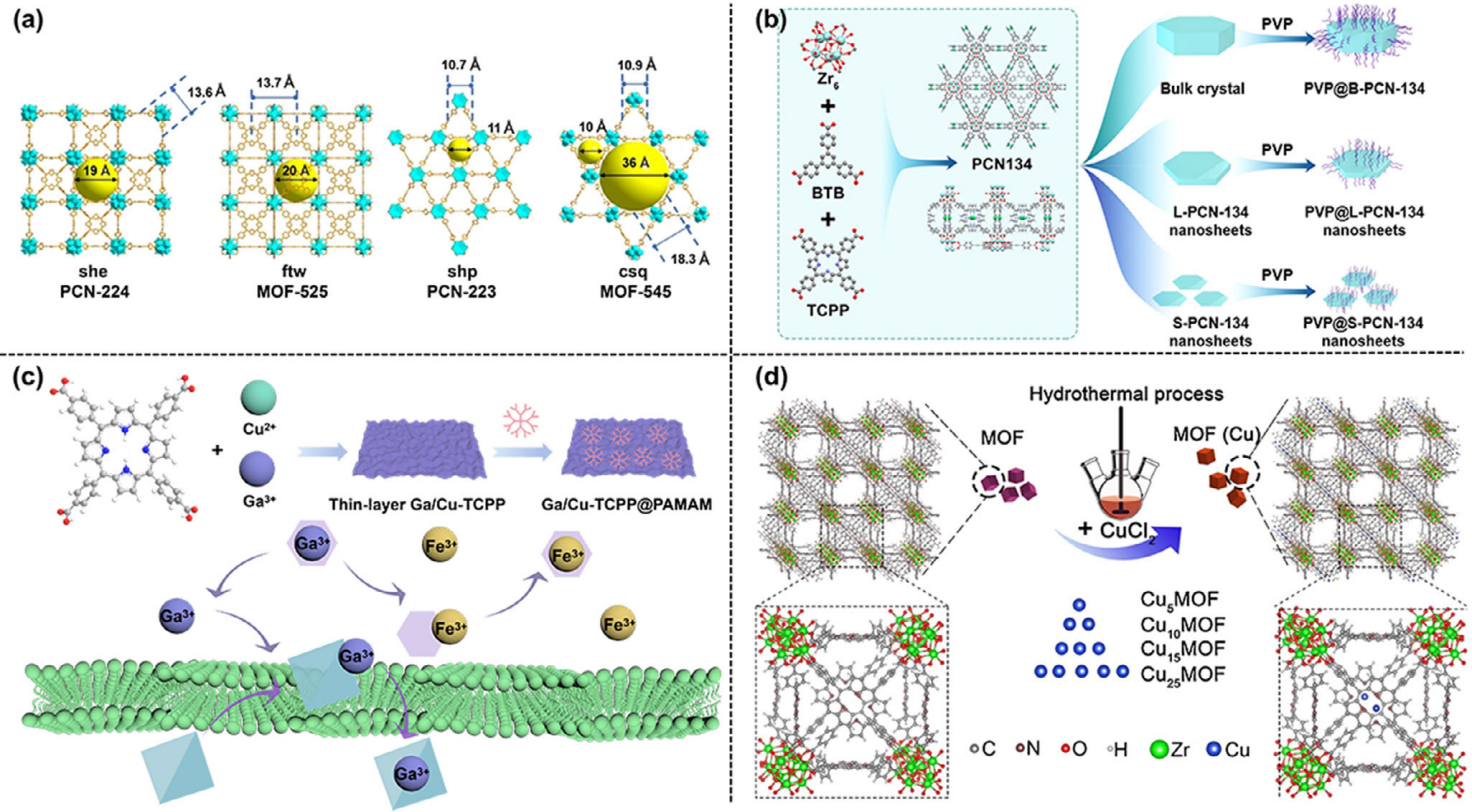
Illustration of PMOFs for accelerated wound healing. (a) The different topological structures of the PMOFs (photodynamic performance from strong to weak: MOF‐545, MOF‐525, PCN‐224, PCN‐223). Reproduced with permission [171]. Copyright 2021, Wiley‐VCH. (b) The schematic illustration of the synthesis and structures of three PCN‐134 nanosheets. Reproduced with permission [91]. Copyright 2023, Elsevier. (c) Schematic illustration of the fabrication and synergistic PDT/PTT/INIT antibacterial mechanism of Ga/Cu‐TCPP@PAMAM. Reproduced with permission [105]. Copyright 2024, Wiley‐VCH. (d) Schematic illustrating the preparation methods, crystal structure, and network topology of MOF and Cu_10_MOF. Reproduced with permission [98]. Copyright 2020, Elsevier. Created with ChemDraw, Autodesk 3ds Max, and Microsoft PowerPoint.

To investigate the effect of PMOF size on photocatalytic activity, Zhou et al. synthesized three PMOFs, including the small nanosheets (S‐PCN‐134), large PCN‐134, and the bulk PCN‐134 crystal in 2023 (Figure 6b). By controlling the reaction temperature to regulate morphology, they investigated the influence of size on PDT properties. By comparing the ROS yield, S‐PCN‐134, with a lateral size of 160–180 nm and a thickness of 9.1–9.7 nm, shows much higher photodynamic and antimicrobial properties [91]. This indirectly demonstrates that small‐sized antimicrobial agents possess both large specific surface areas and high ROS production, offering a novel direction for the development of new antimicrobial agents. Cu‐based MOFs have also been extensively studied. Wang et al. synthesized a single Zn site nanozyme MOF (SZN‐MOFs) with ZnTCPP as ligands and Cu_2_(COO)_4_ clusters as the metallic vertices. Especially, the researchers explain the POD‐like activity and catalytic mechanism of SZN‐MOFs by density functional theory (DFT) calculations [52]. Under low‐dose hydrogen peroxide (H_2_O_2_) assistance, SZN‐MOFs exhibit significant anti‐biofilm activity, with biofilm survival rates below 10% in vitro experiments. Furthermore, in vivo biofilm infection studies confirm that SZN‐MOFs effectively promote wound infection healing. In 2024, Wang et al. proposed a new strategy of “give‐and‐take” to deceive bacteria (Figure 6c). In bacterial infections, Fe^3+^ is essential for bacterial growth. In response to the strategy of Fe restriction by the host, bacteria develop an adaptive method of “utilizing heme to take Fe^3+^” to maintain their growth. The researchers designed a Ga/Cu‐TCPP MOF using Ga‐TCPP and Cu^2+^. On the one hand, the ionic radii of Ga^3+^ and Fe^3+^ are similar, and the porphyrin and heme structures are similar; the porphyrin ring binds more strongly to Fe^3+^ than to Ga^3+^. Therefore, when Ga/Cu‐TCPP contacts bacteria, Ga^3+^ is released, and Fe^3+^ is ingested, achieving iron nutritional immunity therapy (INIT) effectively. Moreover, polyamine amine polymer dots (PAMAM) serve as monitors, further combined with Ga/Cu‐TCPP MOFs to synthesize Ga/Cu‐TCPP@PAMAM, which can realize “three‐in‐one” therapy. This strategy of disrupting bacterial nutritional homeostasis also supports the new concept for antibacterial applications [105]. Zhou et al. proposed a defect engineering strategy by introducing the competitive ligand carvacrol into the Al‐TCPP MOF constructed from TCPP and Al^3+^ [172]. Carvacrol and Al^3+^ compete for coordination sites, partially displacing TCPP while simultaneously creating oxygen vacancies. These vacancies generate defects that modulate excited‐state energy distribution, promote intersystem crossing, and facilitate triplet exciton formation. Consequently, ROS production increases, enabling highly efficient PDT antibacterial activity. To impart targeting properties to PMOFs, Jiang et al. introduced the CuTCPP and boric acid ligands into the MOF and built a multivariate TCPPCu‐BBDC MOF. The boric acid can specifically combine with sugars on the surface of bacteria, overcoming the limitation of the diffusion distance of ROS. Cu^2+^ utilizes the generation of ROS by PDT and promises adaptive biocompatibility. Compared with the control group without boric acid ligands and Cu^2+^, the antimicrobial efficiencies of TCPPCu‐BBDC MOFs are nearly 10–20 times [93].

Introducing different metal ions into the structure of PMOFs for post‐coordination is an effective way of enhancing their photocatalytic and antibacterial properties. Han et al. introduced Cu^2+^ into the porphyrin rings by coordination and realized the incremental PTT and PDT (Figure 6d), which can be attributed to the effective electron capture and the enhanced light absorbance by the d‐d transition. Notably, the researchers found that the light absorbance ability is strongest for Cu_10_MOF, which is the result of the balance between “the light absorption effect of Cu^2+^” and “the high symmetry of the metallized porphyrin rings” by adjusting the different doping amounts. Antibacterial experiments also indicate that Cu_10_MOF can effectively kill *Escherichia coli* (*E. coli*) as well as *Staphylococcus aureus* (*S. aureus*) after 20 min under 660 nm light irradiation [98]. Additionally, Tong et al. incorporated Mn^2+^ into the PCN‐224 to construct Mn@PCN‐224 [120]. This material exhibits both outstanding NIR photothermal conversion capabilities and superoxide dismutase (SOD)‐ and catalase (CAT)‐like enzymatic activities. Following photothermal bacterial inactivation, it effectively scavenges ROS and alleviates oxidative stress, thereby achieving synergistic antibacterial and anti‐inflammatory treatment. Experiments combined with theoretical calculations confirmed that Mn doping endows the material with NIR‐responsive photothermal effects by enhancing NIR absorption, suppressing electron‐hole recombination, and reducing the optical bandgap. This smart nanoplatform also demonstrates significant application potential for treating bacterial wound infections.

Although the therapeutic methods of single PMOFs nanoplatforms have made significant progress, they are also limited by the hypoxic microenvironment, a single therapeutic method, and poor stability. Therefore, researchers have developed multi‐functional PMOFs hybrids with cascade and synergistic effects by combining PMOFs and other materials, such as NPs, metal oxides, polymers, and some carriers [85, 173]. Cascade catalysis refers to an antimicrobial agent integrating two or more catalytically active sites simultaneously. Different active sites sequentially transform the substrate through multi‐step reactions, ultimately generating toxic bactericidal species to achieve highly efficient killing with a synergistic effect where “1 + 1 > 2” [148]. Consequently, numerous precious metal active sites with nanoenzyme activity have been incorporated into porphyrin‐based porous material system designs. Zhu et al. designed Au‐Pt dual‐metal nanozymes (LL‐37‐HA‐Au‐Pt@PCN‐224) to promote the healing of bacterial‐infected wounds using PCN‐224 as carriers (Figure 7a). Among them, Au NPs with POD‐like activity can facilitate H_2_O_2_ to produce ·OH. Pt NPs exhibiting CAT‐like activity can catalyze the conversion of H_2_O_2_ into O_2_, which can alleviate the microenvironment of an infectious wound. Then, generated O_2_ can be used as a reactant to produce the ^1^O_2_ by the photodynamic properties of PCN‐224. Therefore, this cascade catalysis can achieve effective antibacterial activity [99]. Precious metal Ir NPs with multiple enzymatic activities were applied for antibacterial and therapeutic purposes in diabetic wounds. Li et al. utilized PCN‐224 as a carrier, growing Ir NPs in situ on the surface and further modifying it with quaternized chitosan to fabricate PCN@Ir‐QCS (PIQS) [119]. It has glucose oxidase (GOx) activity and photodynamic performance. Additionally, Ir NPs exhibit dual enzyme activity, including CAT and POD, catalyzing locally high concentrations of H_2_O_2_ to produce O_2_ and ·OH at diabetic wounds. This cascade catalytic system can achieve effective therapy for diabetic wounds. Notably, PIQS also show SOD activity during therapy, which plays a crucial role in ROS scavenging and hypoxia alleviation. Chen et al. grew Au nanoclusters (NCs) with Fenton‐like activity on the surface of PCN‐224 to devise a nanoplatform for diabetic wound healing (Au NCs@PCN). Au NCs are responsive to endogenous H_2_O_2_ to produce ·OH and O_2_; the latter provides raw materials for the PDT of PCN‐224, and they also show more potential for synergistic PTT, fully showing the activities of metal NCs [87]. Ag NPs are also widely researched due to their physicochemical and antimicrobial properties [121]. Dong et al. constructed multivariate PMOFs (Ag@UT) by introducing TCPP into UiO‐66 and further modifying Ag NPs on its surface (Figure 7b). Ag@UT has a narrower band gap and enhanced SDT effect because of the effective separation of electrons and holes under ultrasound. Therefore, Ag@UT can significantly accelerate the healing of wounds in *S. aureus* infection [100]. Prussian blue (PB) with a photothermal effect is applied in the construction of PMOFs hybrid materials [104]. Wu et al. designed a dual MOF structure using PB MOF with photothermal effect as the core and porphyrin‐doped UiO‐66 with photodynamic effect as the shell (PB@MOF). Benefiting from the unique heterogeneous interface between the two, the porphyrin structure of the shell can produce more ROS under 660 laser irradiation due to it effectively promotes the transfer of photo‐excited electrons from PB and photocatalytic activity [89]. In the treatment of rat wounds, PB‐PCN‐224 achieved complete wound healing after 14 days. Furthermore, Dong et al. designed Cu‐TCPP@MnO_2_ nanocomposites to effectively overcome hypoxia limitations in PDT [115]. MnO_2_ can respond to the weakly acidic environment of infected wounds and react with overexpressed H_2_O_2_ to generate O_2_, H_2_O, and Mn^2+^. Both Mn^2+^ and Cu^2+^ exhibit Fenton‐like activity and can catalyze the production of ·OH from H_2_O_2_. Additionally, the endogenous glutathione (GSH) can be consumed by these reactive species, thereby reducing GSH‐mediated ROS scavenging and enhancing the overall antibacterial effect. Under dual laser treatment using 660 nm and 808 nm, Cu‐TCPP@MnO_2_ can achieve a triple integrated antibacterial effect combining PTT/PDT/CDT. Although porphyrin‐based porous materials have broad absorption in the visible region, their penetration into biological tissues is limited, which also prevents their further applications. Lanthanide‐doped upconversion nanoparticles (UCNPs) have become good nanomaterials for coupling with PMOFs due to their tunable luminescence properties, large Stokes shift, and high biocompatibility [102, 174]. Wang et al. fabricated a UCNPs@Zr‐MOF core‐shell structure, modified it using Pt NPs, and further loaded it into hydrogels (UCNPs@ZrMOF‐Pt). Pt NPs can respond to overexpressed H_2_O_2_ in infected wounds to generate O_2_, which serves as a reactant to achieve PMOFs cascade photodynamic antibacterial activity under NIR [88].

**FIGURE 7:**
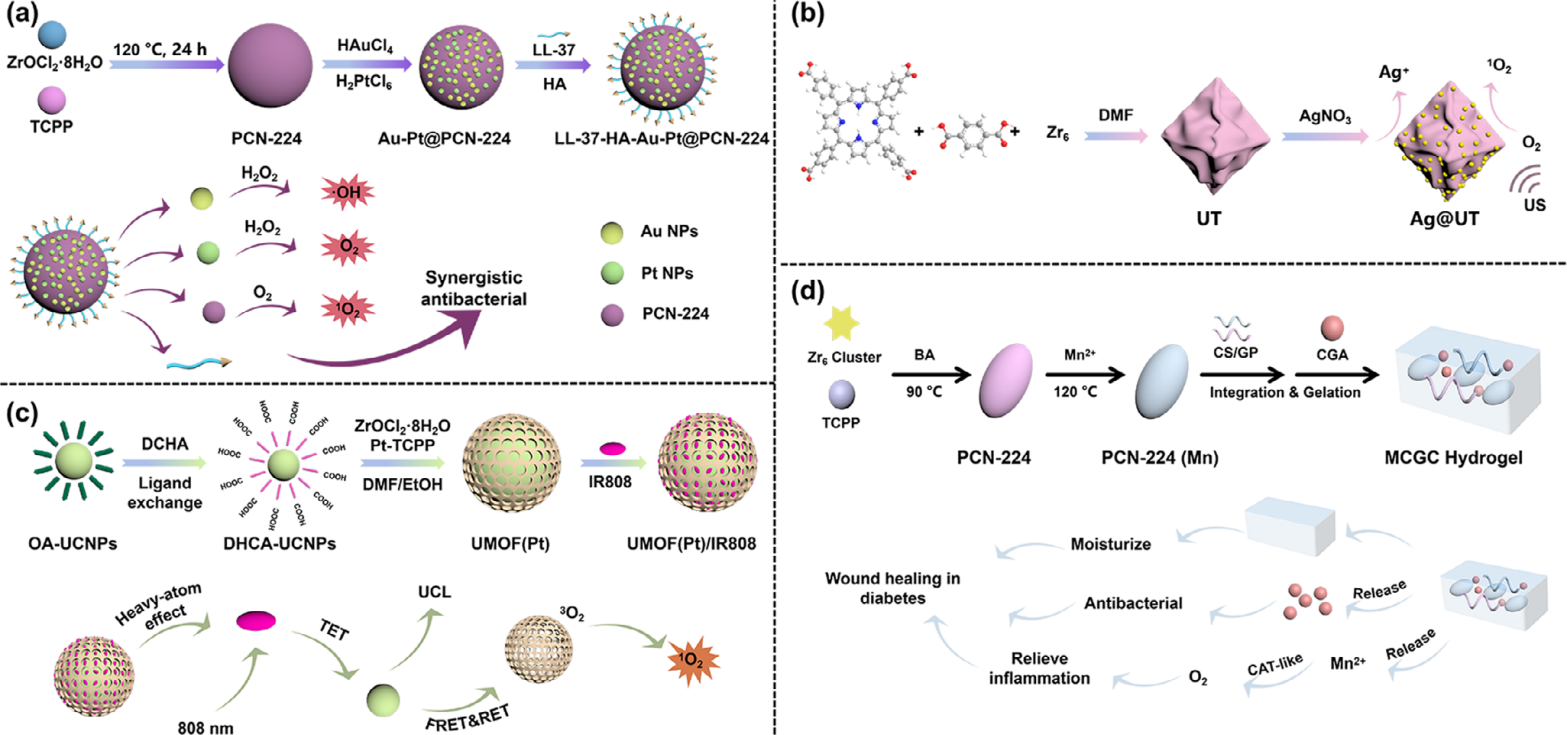
Illustration of PMOF‐based hybrid antibacterial agents for wound healing. (a) Schematic illustration of the preparation and cascade catalytic antibacterial mechanisms of LL‐37‐HA‐Au‐Pt@PCN‐224. Reproduced with permission [99]. Copyright 2024, Elsevier. (b) Schematic illustration of the fabrication of Ag@UT and its antibacterial mechanisms via enhanced SDT. Reproduced with permission [100]. Copyright 2024, Elsevier. (c) Schematic illustration of the structural design and cascade energy transfer of UMOF(Pt)/IR808. Reproduced with permission [109]. Copyright 2024, Elsevier. (d) The synthetic routes of MCGC hydrogel for accelerating wound healing. Reproduced with permission [110]. Copyright 2024, Wiley‐VCH. Created with ChemDraw, Autodesk 3ds Max, and Microsoft PowerPoint.

Building upon this foundation, Chen et al. integrated UCNPs, Pt atoms, and PMOFs, and further loaded a NIR heptamethine cyanine dye (IR808) to generate the cascade energy conversion nanoplatform (UMOF(Pt)/IR808) (Figure 7c). Under the irradiation of an 808 laser, ^1^O_2_ is generated using cascade energy transfer from IR808 to UCNPs and further to PMOFs. Remarkably, UMOF(Pt)/IR808 can achieve effective inactivation of Methicillin‐resistant *Staphylococcus aureus* (MRSA) even at a very low power density (0.4 W cm^−2^) [109].

In the treatment of certain chronic wounds caused by drug‐resistant bacterial infections, carriers such as hydrogels, electrospun fibers, and various dressings are widely used to deliver antimicrobial agents [175]. This is because they can create a stable physiological microenvironment, enhance biocompatibility, optimize drug delivery systems, and reduce the risk of infection [176]. As a biomaterial with high water content, good biocompatibility, and excellent porosity, hydrogels are used as antibacterial agent carriers. Hydrogels are characterized by non‐adherence to the wound, which avoids secondary damage to the wound during removal [177, 178]. Consequently, there are many advantages to hydrogels as antibacterial carriers. Liu et al. co‐loaded the PCN‐224 (Mn) MOF and chlorogenic acid into the hydrogel composed of genipin and chitosan (MCGC hydrogel) (Figure 7d). Among them, PCN‐224 (Mn) MOF nanozymes possess CAT‐like activity in response to excess H_2_O_2_ in diabetic wounds, further alleviating oxidative stress and hypoxia microenvironment. Subsequently, chlorogenic acid acts as an antimicrobial inhibitor that promotes skin regeneration and wound healing.

The hydrogels also provide a moist microenvironment and absorb excess exudate [110]. Shen et al. loaded curcumin (Cur) into CuPP MOF to construct a hybrid hydrogel dressing with poly(ethylene glycol)‐poly(ε‐caprolactone‐*co*‐lactide) (Cur/CuPP‐PELA) (Figure 8a) [117]. The hydrogel exhibits a reversible sol‐gel transition behavior; it remains in a liquid state at room temperature (22°C) and rapidly gels at body temperature (37°C). Utilizing the photothermal ability of CuPP, the antibacterial and anti‐inflammatory activities of Cur, as well as the thermo‐responsive, hemostatic, and biocompatible properties of the hydrogel, this composite enables treatment of triclosan‐resistant *Pseudomonas aeruginosa* (*P. aeruginosa*) bacteria within 14 days. In addition, to solve the difficult issue of chronic wound healing, Li et al. synthesized a multifunctional microneedle patch (M/DP MN patch) in which dimethyloxalylglycine (DMOG) was loaded into PCN‐224 and combined with hyaluronic acid (HA) and meropenem (MEM) (Figure 8b). Notably, this MN patch can non‐invasively penetrate the skin barrier and dissolve rapidly within 10 min. Upon contact with the wound, the MN patch dissolves and releases PCN‐224 and MEM after contact with the wound automatically, completing the synergistic antibacterial properties of PDT and drug therapy. Among these, DMOG acts as a promoter capable of continuously stimulating angiogenesis in circular wounds of mice. This not only reduces the dose of the drug used but also offers a new idea for chronic wound healing [94]. Certain electrospun fibers are also widely used as carriers for antimicrobial agents. Jiang et al. introduce Ti into PCN‐224 MOFs to form dual‐metal PCN‐224 (Zr/Ti) using a simple ion exchange strategy, based on the highly oxidized states. Simultaneously, by reducing the bandgap and red‐shifting light absorption, ROS generation was synergistically amplified without requiring additional PSs. Based on the excellent breathability and superior biocompatibility of electrospun fibers, researchers prepared PCN‐224 (Zr/Ti) as a dressing via electrospinning, significantly accelerating the healing of chronic wounds infected with multidrug‐resistant bacteria [55]. Wu et al. prepared Cu‐TCPP using three Cu sources and TCPP, respectively, and loaded them into nanofibers through electrostatic spinning. Cu‐TCPP is characterized by POD‐like activity in response to H_2_O_2_ to generate ·OH. Notably, even at alkaline conditions, Cu‐TCPP can keep a strong deprotonation effect, create a weakly acidic environment, and improve enzyme activity. In vivo experiments, Cu‐TCPP can accelerate the repair of tissues [106].

**FIGURE 8:**
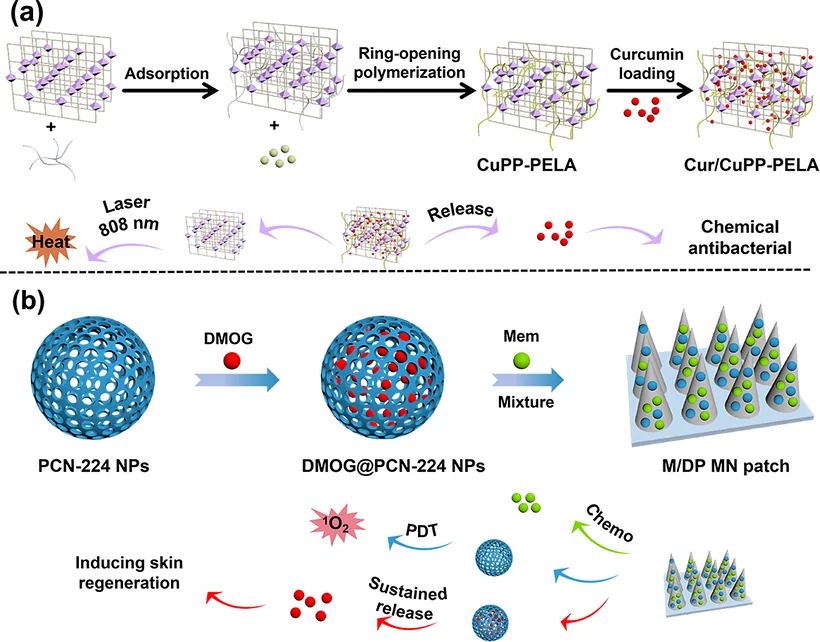
Illustration of PMOF‐based antibacterial materials for wound healing. (a) Schematic diagram of preparation of Cur/CuPP‐PELA thermosensitive hydrogel that can effectively treat chronic wounds. Reproduced with permission [117]. Copyright 2022, Elsevier. (b) Schematic illustration of multifunctional MOF‐based M/DP MN patch for improving chronic wound healing. Reproduced with permission [94]. Copyright 2023, Wiley‐VCH. Created with Autodesk 3ds Max and Microsoft PowerPoint.

Electrodynamic therapy (EDT) has also exhibited great potential in antibacterial treatment, mainly owing to its ROS‐generating capability without the need for H_2_O_2_ and O_2_, only under the effect of an electric field. Xu et al. constructed a biomimetic nanosponge by encapsulating PMOF with extracellular vesicles (EVs) derived from ginger (MOF@EV) [113]. Under the effect of an external alternating electric field, PMOF acts as an “electric sensitizer” to catalyze the production of ROS, achieving EDT. At the same time, EVs serve as “bait” by simulating the host cell membrane structure to adsorb *S. aureus* toxins, reducing tissue damage. In a subcutaneous abscess model, MOF@EV combined with electrokinetic therapy demonstrated outstanding biofilm elimination capability and detoxification efficiency, significantly clearing bacteria and promoting abscess healing within 6 days. Zhu et al. developed a new approach to target macrophages by modifying D‐mannosamine on the surface of CuTCPP@MOF via an amide coupling reaction (CuTCPP@MOF@Mannosamine). Driven by ultrasound, they improve oxidative stress, disrupt biofilms, and achieve the effect against implant‐associated infections [103].

*PCOFs Promote Wound Healing*: Porphyrins, typical PSs with tetrapyrrole macrocycles, are used as rigid structural units to build COFs. Compared with chemical bonds such as borate, triazine, and amide bonds, imine bonds are widely used in antimicrobial research as a classical way of connecting PCOFs [124]. Pristine PCOFs can be condensed by porphyrins with different units to obtain customized structures and functions [130]. By modifying the structure of PCOFs, they can be endowed with diverse functionalities, more active sites, and excellent optical properties. Yan et al. designed a porphyrin‐anthracene COF (Por‐DPA COF) that could be used for sustainable photosterilization. Anthracene, as a great source of ^1^O_2_, is chosen as the capture for ^1^O_2_ and releases it sustainably via conversion of endoperoxide to L21 upon irradiation. Notably, this is the first report of sustained therapeutic effect in phototherapy without continuous light irradiation, enabling effective photodissolution and healing of infected wounds. It also highlights the significant potential of PCOFs in generating sustainable ROS [126]. Qian et al. explored the effect on the structure and antimicrobial properties by modifying different linking groups in the terephthalic acid ligand of COF‐366. They designed three highly photosensitive Por‐COFs, including COF‐366, DhaTph, and JNU‐2, through a conjugation‐regulating strategy by Por1 condensing with L1, L4, and L6, respectively. The last COF (JNU‐2) exhibits the highest degree of conjugation and photosensitizing activity due to p‐π conjugation and the enhancement of intersystem crossing. The antimicrobial results also indicate better photodynamic inactivation by it. This work provides new inspiration for conjugation‐regulating strategies of PCOFs [125]. To avoid the aggregation of porphyrin molecules themselves, researchers have developed a variety of approaches, including many macrocycles in PCOFs. Based on COF‐366, Zhou et al. first prepared β‐cyclodextrin (β‐CD) macrocycle‐based rotaxane COFs (Por‐CD‐COF) through a supramolecular binding strategy (Figure 9a). Por‐CD‐COF was built by both L2 and Por1. Compared with Por‐COF, the introduction of β‐CD avoids quenching of photoactivity and enhances the effective use of PSs by increasing the distance between layers. It also significantly reduces the amount of porphyrin used owing to its inherent antibacterial properties and biocompatibility. Remarkably, Por‐CD‐COF promotes the healing of infected wounds through the β‐CD‐enhanced synergistic antimicrobial properties of PDT and PTT [131]. Similarly, they also prepared a crown‐ether threaded covalent organic polyrotaxane framework (Crown‐COPF‐Zn), where the crown ether acts as a mechanical bond in the molecular machinery and binds to the lipid portion of the bacterial cell membrane, which also has a certain enhancing effect on antibacterial properties [127].

**FIGURE 9:**
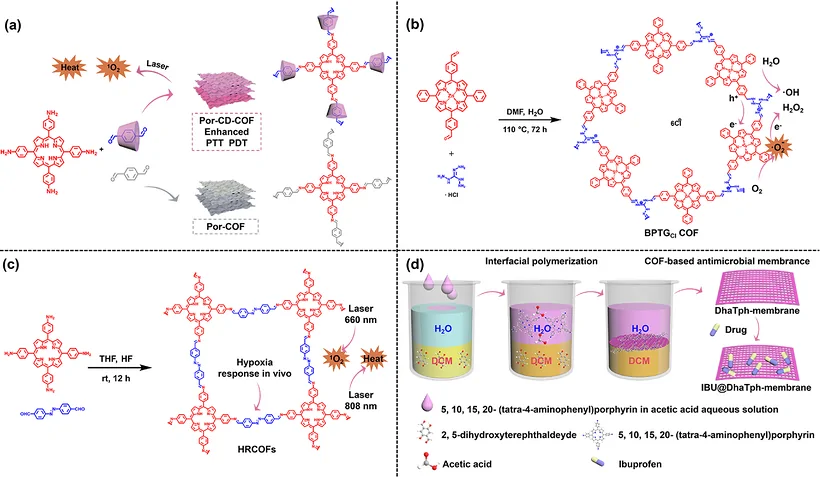
Illustration of multifunctional PCOF‐based antibacterial materials for wound healing. (a) Schematic illustration of the preparation and β‐CD‐enhanced PDT and PTT of Por‐CD‐COF. Reproduced with permission [131]. Copyright 2024, Elsevier. (b) Preparation of BPTG_Cl_ COF with multiple antimicrobial mechanisms. Reproduced with permission [128]. Copyright 2024, American Chemical Society. (c) Schematic diagram of the synthesis route of hypoxia‐activated biodegradable HRCOFs for promoting wound repair. Reproduced with permission [129]. Copyright 2024, American Chemical Society. (d) Schematic illustration of the preparation of diverse antimicrobial IBU@DhaTph band aid via an in situ polymerization at the interface and impregnation strategy. Reproduced with permission [133]. Copyright 2021, Wiley‐VCH. Created with ChemDraw, Autodesk 3ds Max, and Microsoft PowerPoint.

As bacterial surfaces are negatively charged, the researchers introduced various cationic ligands to give the PCOFs targeting capabilities. For example, Feng et al. developed a cationic COF (BPTG_Cl_ COF) that could target bacteria (Figure 9b). Due to the electron transfer interactions between the unique guanidinium and Zn‐porphyrin moieties, BPTG_Cl_ COF exhibits a nucleophilic/electrophilic region that further increases the yield of ROS under light irradiation. The interaction between BPTG_Cl_ COF and bacteria enables selective identification and imaging, and shows high antibacterial activity against Gram‐positive bacteria even at a minimal concentration (2.00 ppm) [128]. Liu et al. utilized a solvent‐mediated dual reaction synthesis strategy to synthesize the cationic metallocovalent organic framework (CRuP‐COF) using L23 and Por9 as raw materials. Its cationic components can more effectively target bacterial surfaces through electrostatic interactions [144]. Due to the unique pace‐twisted structure of L23, the interlocked structure centered on Ru as the metal core effectively suppresses stacking after forming a topological network structure, thereby preventing the attenuation of porphyrin activity. Phototherapy results indicate that CRuP‐COF is characterized by superior self‐amplified synergistic PTT and PDT effects. It achieves an eradication rate of over 98% against both *S. aureus* and *E. coli*, thereby effectively alleviating wound infections. In 2025, Yan et al. developed a new cationic PCOF nanoplatform (TAPP‐VIO) condensed from Por1 and L16 [142]. The two building units synergistically fulfill different roles; the cationic 1,1’‐ benzaldehyde 4,4’‐bipyridinium (VIO) ligands enable specific targeting of negatively charged bacterial membranes and inhibit bacterial motility, while Por1 promotes heat and ^1^O_2_ generation under light irradiation. Moreover, the interlayer electrostatic repulsion introduced by VIO effectively suppresses π‐π stacking, enhancing photodynamic efficiency. Compared with non‐ionic COF, TAPP‐VIO exhibits a 179% increase in ^1^O_2_ yield and a 15% reduction in photothermal ability, demonstrating superior phototherapeutic efficacy with minimized thermal damage. In a bacterial infection mouse model, TAPP‐VIO showed excellent wound‐healing promotion without significant side effects.

A good antimicrobial agent must not only address the issue of specific targeting but also consider its irritation response and in vivo metabolism [145]. Although many COFs are applied to biomedicine, the metabolism and long‐term retention of PCOFs are major problems in vivo, which have been cleverly solved by Zhang et al. through the design of an azophenyl PCOF. They reported a new hypoxia‐activated biodegradable COF (HRCOFs) for synergistic PDT/PTT, and azobenzene groups for degradation (Figure 9c). In the microenvironment of wound infection, HRCOFs not only produce ^1^O_2_ and heat under laser irradiation but also are easily degraded, cleared, and more biocompatible, which lays the foundation for further exploration of the clinical translation of PCOFs [129].

PCOFs as drug carriers can realize the combined phototherapy and drug therapy. In 2021, Dong et al. first prepared a membrane of PCOF that is large‐area, self‐standing, and homogeneous via in situ interfacial polymerization and impregnation strategies (Figure 9d). After loading ibuprofen (IBU), a dressing similar to a “band‐aid” is developed based on the IBU@DhaTph membrane, which exhibits synergistic drug therapy and phototherapy. Remarkably, the effective antibacterial rate can reach 94.2% for *E. coli* and 95.9% for *S. aureus* even under sunlight irradiation [133]. IBU@DhaTph achieves effective relief for chronic wounds infected with *S. aureus* through its self‐adhesive and durable bandage‐like design. In 2025, Jiang et al. prepared water‐soluble MET‐TCPP nanorods (MT‐LYZ) by a condensation reaction using TCPP and L25 as reactants, and further loaded lysozyme (LYZ) [146]. The surface charge of LYZ can change with pH; it transitions from negative to positive in the weakly acidic microenvironment of MRSA biofilm, driving its penetration through the biofilm. Under 660 nm laser irradiation, MT‐LYZ triggers mild PTT and efficient PDT synergistic sterilization, which can achieve tissue repair of MRSA biofilm‐infected wounds. Recently, gaseous therapy (GT) has attracted attention as an emerging therapy, and progress has been made in combination with phototherapy [97, 111, 179]. Compared with directly using antibiotics, many PCOFs are used as carriers to contain stimulus‐responsive gas donors. Yao et al. designed a COF composed of L18 and Por7 linked by a bridge. After loading N,N’‐di‐sec‐butyl‐N,N’‐dinitroso‐1,4‐phenylenediamine (BNN6), they prepared TP‐Por CON@BNN6 for simultaneous induction of PTT, PDT, and GT under red light irradiation. It is also characterized by anti‐inflammatory properties, high biocompatibility, and chronic wound repair ability [132]. Among them, BNN6 is loaded as a NO donor molecule, which is shown to reduce inflammation, kill pathogens, and promote wound contraction. Notably, TP‐Por CON@BNN6 spontaneously metabolizes into small molecules in vivo, demonstrating excellent biodegradability. This property also lays a crucial foundation for developing combined phototherapy and GT treatment regimens.

PCOFs, as periodic topologies, have made great progress in the field of drug delivery, multiple pore structures of PCOFs are still rarely reported. To fill the gap in this field, Han et al. innovatively designed a DEG‐HEP‐COF (with both hydrophilic alkoxy chain and hydrophobic alkyl chain) with hetero‐environment pores using Por1 and L24 (Figure 10a). The researchers further calculated the intra‐layer chain orientation of DEG‐HEP‐COF and showed that the total energy is minimized when the same chains are arranged in the same pore. Owing to its unique and ideal pores, hydrophobic levofloxacin (Lev) and hydrophilic vancomycin (Van) were co‐loaded (DEG‐HEP‐COF@Lev&Van). Upon contact with the wound, the hydrophilic drug is rapidly released, while the hydrophobic drug provides sustained release, synergistically promoting healing of MRSA‐infected wounds. This not only enriches the diversity of pore structure but also provides insights into the functional expansion of PCOFs [134].

**FIGURE 10:**
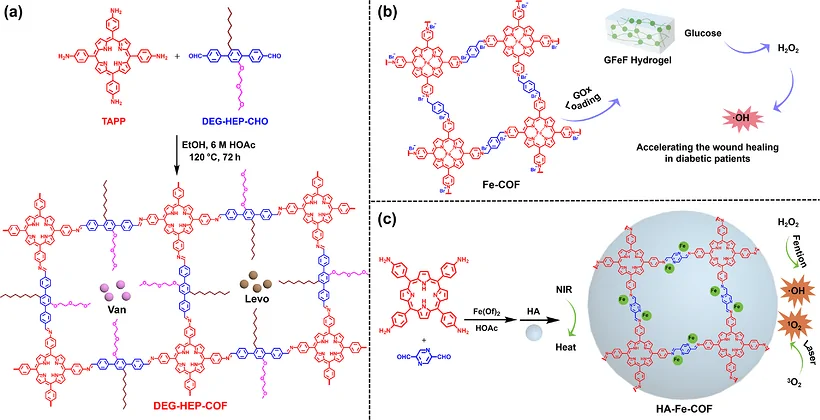
Illustration of PCOF‐based hybrid platforms via pore design and metal modification. (a) Schematic illustration of the synthesis of DEG‐HEP‐COF@Lev&Van with hetero‐environmental pores. Reproduced with permission [134]. Copyright 2023, Springer Nature. (b) Schematic representation of the preparation and cascade catalytic diabetic wound healing of GOx‐on‐Fe‐iCOF (GFeF) hydrogel. Reproduced with permission [180]. Copyright 2021, Wiley‐VCH. (c) Schematic illustration of the preparation process of HA‐Fe‐COF. Reproduced with permission [137]. Copyright 2024, American Chemical Society. Created with ChemDraw, Autodesk 3ds Max, and Microsoft PowerPoint.

In addition to the rational design of porous structures in PCOFs for drug delivery, developing PCOF‐based hybrid materials represents another effective strategy to enhance material performance. By integrating PCOFs with functional components such as natural enzymes, metal ions, and hydrogels, the resulting hybrid composites can combine multiple therapeutic modalities, including cascade catalysis, phototherapy, CDT, and responsive drug delivery. These features significantly improve antibacterial efficacy and facilitate the repair and healing of chronically infected wounds. For example, Xie et al. reported a GOx‐on‐Fe‐iCOF (GFeF) containing porphyrin‐based ionic COF (iCOF) chelated with Fe^2+^, which was further loaded with GOx via electrostatic interactions (Figure 10b).

GOx can catalyze excess glucose into gluconic acid and H_2_O_2_, the latter of which can be converted to toxic ·OH by Fe^2+^. To promote the difficult wound healing of diabetic patients, the researchers loaded it into a hydrogel for the therapy of bacterial wound infections in vivo. In vivo experiments demonstrate that this glucose‐triggered cascade catalytic strategy can effectively accelerate wound healing in diabetic patients [180]. GOx exhibits better catalytic activity, but it is expensive and rare. Nanozymes, as emerging nanomaterials with enzyme‐like activity, fill the gap of natural enzymes. Not only can they catalyze all kinds of chemical reactions, but they are also characterized by low cost, excellent stability, and great catalytic activity. For instance, Zhang et al. prepared two Zn^2+^ coordination PCOFs, including Se–Se COF_Zn_ and S–S COF_Zn_, further decorating tannic acid and aminophenylboronic acid‐modified HA to form a boronate‐bonded COF_Zn_@HPTA hydrogel. COF_Zn_@HPTA shows high GOx‐like activity and photothermal transformation capacity, achieving a decrease in blood glucose levels and enhanced antimicrobial activity. Furthermore, the S–S/Se–Se bonds can respond to ROS to release Zn^2+^ with tissue‐repairing properties. Combined with a hydrogel, COF_Zn_@HPTA effectively promotes wound healing in diabetic wounds [140]. Additionally, Xiong et al. synthesized HA‐Fe‐COF via Schiff base condensation between Por1 and L3, which provided an active site for anchoring Fe atoms (Figure 10c). The introduction of Por1 increases the degree of backbone conjugation and enhances light‐harvesting ability. HA‐Fe‐COF not only possesses POD‐like activity but also exhibits excellent photothermal effect and ROS generation efficiency when exposed to dual light sources [137]. Utilizing triple‐modal synergy, HA‐Fe‐COF exhibits high antibacterial performance against *S. aureus*, even in a high‐sugar environment.

In 2025, Qiu et al. prepared poly (osmium porphyrin) (Polypor (Os)) by doping trace amounts of Os (0.46 at%) based on the multi‐porphyrin substrate COF (Por1 + Por8) (Figure 11a) [141]. Polypor (Os) possesses both POD‐like and CAT‐like dual enzyme activities. The former catalyzes the production of ·OH from H_2_O_2_, while the latter catalyzes the production of O_2_ from H_2_O_2_, relieving the hypoxic microenvironment of infected sites. Based on this, the O_2_ dependence of SDT based on Polypor (Os) has been successfully overcome. It is worth noting that in cell and animal experiments, Polypor (Os) showed good biocompatibility and no pathological changes in the organization. This also marks a breakthrough for Os in the biological field. Moreover, SAs can also be introduced as nanoenzymes into PCOF systems, achieving enhanced photocatalytic activity and synergistic effects. In 2025, Yue et al. constructed PCOFs by Schiff base condensation of porphyrin and phenanthroline, and further encapsulated them with mPEG‐NH_2_ (CuSA‐COF) [143]. Introducing CuSA active sites through coordination between ortho‐phenanthroline N and Cu. The metal protonation mechanism of CuSA can create an acidic microenvironment at physiological pH, significantly enhancing POD‐like activity. Additionally, 635 nm laser irradiation can trigger the synergistic action of type II PDT and the Fenton‐like reaction, rapidly generating a large amount of ROS, while depleting GSH and inhibiting glutathione peroxidase 4, jointly driving lipid peroxidation accumulation and inducing bacterial ferroptosis. To compensate for the limited light absorption capacity of PCOFs, Ag NPs were incorporated into their structure to modulate the bandgap energy and facilitate charge transfer. Ai et al. designed an Ag@ZnPor‐COF by modifying the Ag NPs on the ZnPor‐COF surface (Figure 11b). Ag NPs present a high localized surface plasmon resonance effect, which can realize the effective isolation of photogenerated electrons and holes and significantly enhance the ROS generation and photothermal conversion of PCOFs. Subsequently, Ag NPs can also interact with bacterial cell membranes, synergistically enhancing antibacterial activity. Under visible light exposure, the effective antibacterial rate of Ag@ZnPor‐COF can reach 99.96% and 99.75% for *S. aureus* and *E. coli* for 20 min, respectively [135].

**FIGURE 11:**
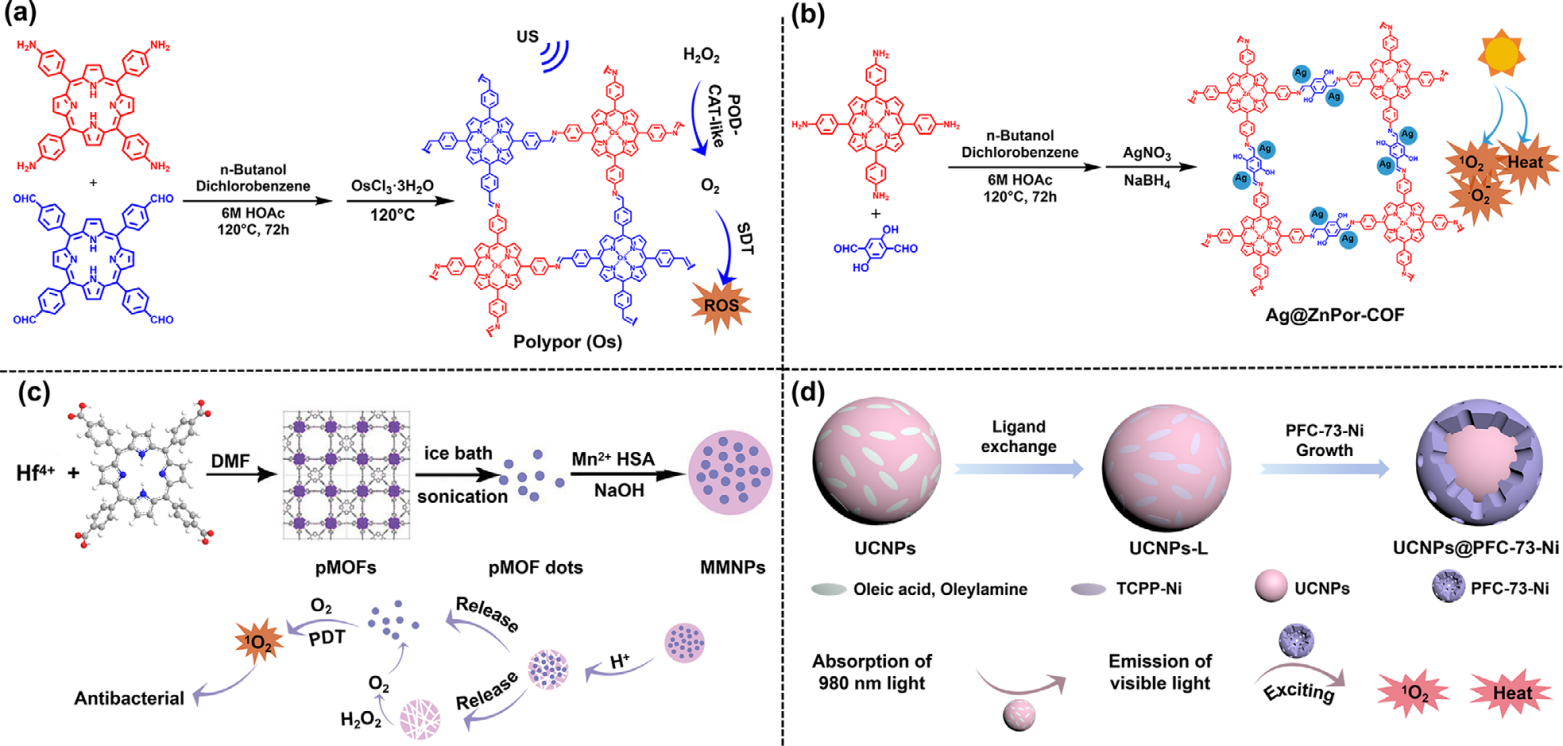
Illustration of metalation and nanohybrid modified porphyrin‐based porous materials for antibacterial therapy. (a) Schematic of Polypor (Os) preparation and its metalation. Reproduced with permission [141]. Copyright 2025, Wiley‐VCH. (b) Schematic diagram of the ROS generation and photothermal effect by Ag@ZnPor‐COF under visible light. Reproduced with permission [135]. Copyright 2024, Elsevier. (c) Scheme illustration of the preparation process of MMNPs for photodynamic eradication of bacterial biofilm. Reproduced with permission [108]. Copyright 2019, Wiley‐VCH. (d) Schematic illustration of core‐shell UCNPs@PFC‐73‐Ni and its antibacterial principle. Reproduced with permission [154]. Copyright 2024, Wiley‐VCH. Created with ChemDraw, Autodesk 3ds Max, and Microsoft PowerPoint.

To improve the penetration and diffusion of the porphyrin in biofilms and increase the utilization efficiency, researchers make great efforts to mitigate the hypoxic microenvironment. Ren et al. first designed and synthesized PMOF dots and encapsulated them via human serum albumin‐coated MnO_2_ to develop cascade‐enhanced photodynamic nanoplatforms (MMNPs) (Figure 11c). In the slightly acidic environment of biofilms, the diffusion distance to PMOF dots is shorter, and MnO_2_ is degraded by endogenous H_2_O_2_ to produce O_2_, achieving enhanced PDT antibacterial effects. At the same time, the negative charges are released to improve biofilm accumulation and the yield of ROS. The researchers also used MMNPs to treat *S. aureus* infections in subcutaneous abscesses and showed that they do not damage healthy tissue [108]. In addition to targeting negatively charged bacteria through electrostatic interactions, certain covalent binding methods can also achieve targeting functionality. Zhao et al. reported an acrylate‐modified PCOF nanoplatform (Por‐COF‐Gel) that covalently binds to the bacterial surface and prevents bacterial colonization and spread. The researchers introduce acrylate groups into L20, which not only immobilize the bacteria by Michael addition to the surface of PCOFs, but also enhance the bactericidal effect of ROS. The survival rate of bacteria was almost “0” after 30 min under white light irradiation, depending on synergistic PTT/PDT and drug therapy [139]. In vivo experiments demonstrate that Por‐COF‐Gel can achieve complete healing of *S. aureus*‐infected wounds within 10 days.

Most traditional PCOF antimicrobial agents are water‐insoluble and can only catalyze reactions heterogeneously, leading to low catalytic efficiency. To address this issue, Zhang et al. developed a water‐soluble Zn‐based PCOF nanohydrogel (Zn‐COF‐NHG) [147]. They achieved uniform dispersion and homogeneous catalysis of PCOFs in aqueous solution by grafting the thermosensitive polymer poly(N‐isopropylacrylamide) onto brominated PCOFs via in situ polymerization. Zn‐COF‐NHG exhibits excellent oxidase‐like activity and efficiently generates ^1^O_2_ under visible light irradiation. It shows potent broad‐spectrum antibacterial activity at low concentrations and effectively promotes wound disinfection and healing. Moreover, its temperature‐responsive property enables intelligent regulation of catalytic activity, providing a new strategy for safe and efficient antibacterial therapy and wound repair.

*PHOFs and APPPs Promote Wound Healing*: Although there have been relatively few reports on the use of PHOFs and amorphous APPPs in biology, both can be used to prepare antimicrobial agents [153]. Cao et al. also reported a core‐shell structure (UCNPs@PFC‐73‐Ni) using UCNPs and PFC‐73 HOF (Figure 11d). UCNPs can be effectively converted into visible emission under NIR irradiation to excite porphyrin for PDT and PTT. UCNPs@PFC‐73‐Ni realizes the effective fluorescence resonance energy transfer pathway and the strategy of “1 + 1 > 2.” This also demonstrates the promising application prospects of HOF functionalization in achieving enhanced antibacterial properties [154]. Liu et al. constructed anionic PHOF (PFC‐33) for the first time using TCPP to solve the problem of introducing functional species into conventional PHOFs without changing the original topology. Subsequently, the researchers further filled the pores with quaternary ammonium as a counterion via electrostatic interactions, thus realizing the integration of PDT and drug therapy. In addition, the researchers also modified the residual carboxyl groups on the surface of the PHOF particles and further copolymerized them with poly(ethylene glycol) to form a flexible, free‐standing PHOF membrane. Remarkably, this anionic PHOF membrane exhibits high flexibility and stability, and serves as inspiration for the structural design and the fabrication of PHOF membranes [152].

Zhou et al. used Py and diformyl‐dibenzo‐18‐cry‐6 to synthesize a nanorod‐like polymer, further modified it with silver nitroprusside NPs (CEP‐POP‐AgNNP), featuring intrinsic antibacterial activity. The crown ether moiety is introduced as an anchor for Ag^+^ localization and as an intrinsic antimicrobial structure. CEP‐POP‐AgNNP is characterized by high stability, good compatibility, and effective sterilization, so that it can achieve a synergistic strong inhibition effect against *S. aureus* and *E. coli* even at a low concentration of 5 µg mL^−1^ [161]. Zhou et al. devised a multifunctional polymer (Fc‐PP‐POP) containing porphyrin and ferrocene units, with synergistically superior photothermal and POD‐like activity. Fc‐PP‐POP can effectively respond to H_2_O_2_ to produce ·OH and achieve 46.54% photothermal conversion efficiency under 635 nm irradiation. Furthermore, Fc‐PP‐POP serves as a broad‐spectrum bactericide, demonstrating significant antimicrobial efficacy against *S. aureus* (98.83%) and *E. coli* (86.89%) [181]. Additionally, Ju et al. designed a cationic polymer (BDPP) with high PTT effects to improve the healing of an infectious wound [182].

#### 3.1.2 Treatment of Osteomyelitis

Osteomyelitis is an inflammatory bone tissue disease caused by bacterial infection, potentially leading to lifelong disability or fatal sepsis [122]. Current treatment approaches for osteomyelitis are diverse, primarily involving surgical intervention and antibiotic therapy. However, due to complex clinical presentations and antibiotic resistance issues, it has become one of the most challenging conditions in orthopedics. Consequently, developing novel materials to enable antibiotic‐free and non‐invasive treatment approaches has emerged as a key research focus in osteomyelitis therapy in recent years. Porphyrin‐based porous materials, which combine deep penetration capabilities with high ROS production, have become a cutting‐edge platform for osteomyelitis treatment. Due to their non‐invasive therapeutic nature, these materials have also been applied in recent years to address diseases related to osteomyelitis. Compared to PDT and PTT, SDT is more widely used in deep osteomyelitis [112]. This may be because it can penetrate 5–10 cm of dense cortical bone and achieve precise targeting, making it the preferred antimicrobial approach for bone infections. For example, Wu et al. constructed four PMOFs by introducing defects through modulators, benzoic acid (BA) and acetic acid (HOAc), during the synthesis of PMOFs (Figure 12a). The structure of PMOFs changes from PCN‐222 to PCN‐224 as the amount of modulator increases. Notably, PCN‐222/PCN‐224 (D‐PCN‐2) achieves a two‐phase homojunction structure, which exhibits the best sonocatalytic antibacterial performance due to the minimum band gap and maximum production of ultrasound electrons. Based on its excellent biocompatibility and sonodynamic properties, researchers applied it to treat a model of MRSA‐infected osteomyelitis characterized by hypoxia and biofilm encapsulation. In vivo experiments demonstrated that D‐PCN‐2 can effectively treat MRSA‐infected osteomyelitis with SDT to prevent bone destruction [92].

**FIGURE 12:**
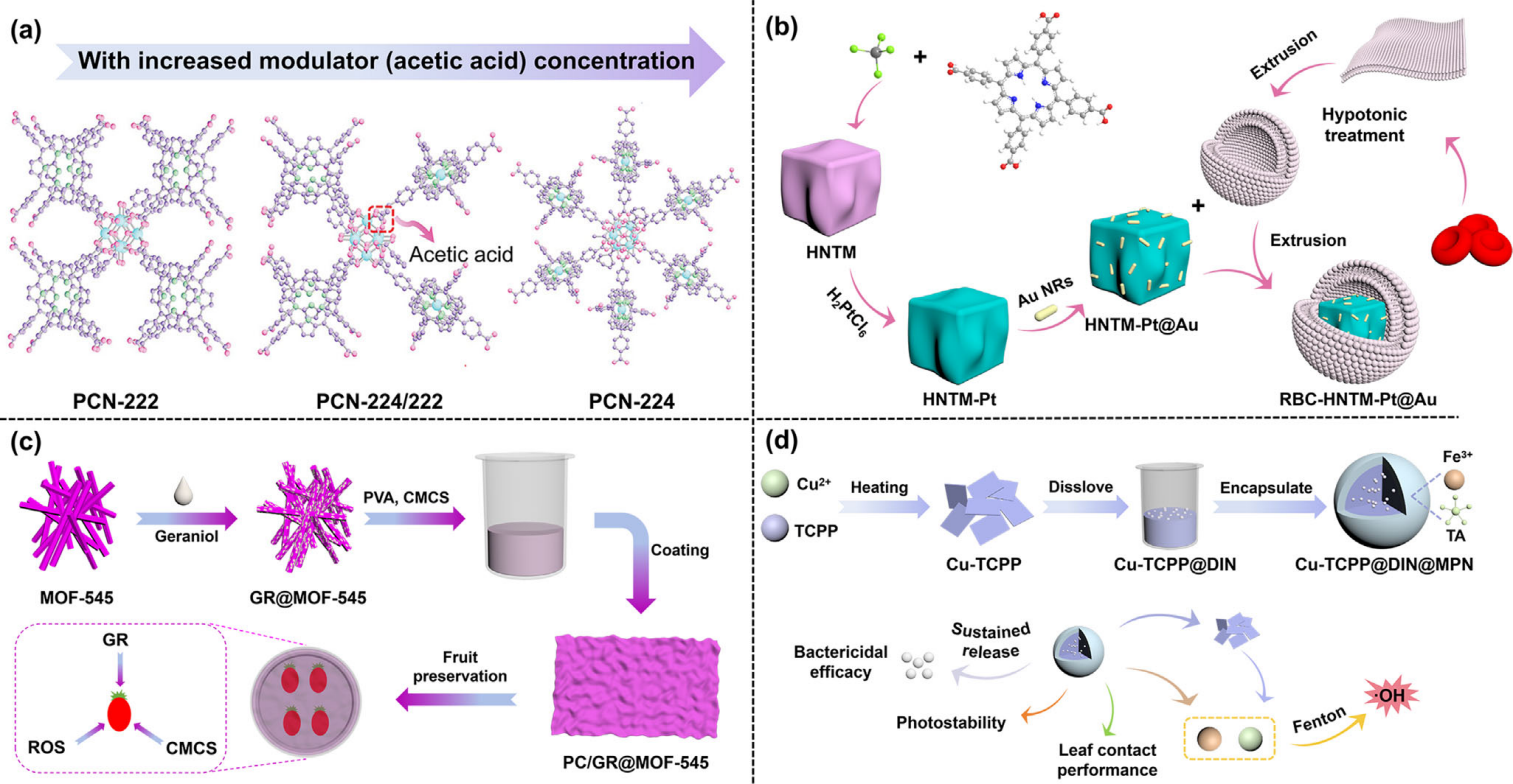
Illustration of porphyrin‐based porous materials for the treatment of osteomyelitis, food preservation, and agricultural disease control. (a) The schematic illustration of MOF crystal structures as the HOAc concentration increases. Reproduced with permission [92]. Copyright 2023, Wiley‐VCH. (b) Schematic illustration of RBC‐HNTM‐Pt@Au synthesis with sonocatalytic activity. Reproduced with permission [36]. Copyright 2021, American Chemical Society. (c) The fabrication process of PC/GR@MOF‐545 and the preservation mechanism of cherry tomatoes. Reproduced with permission [35]. Copyright 2024, Elsevier. (d) Schematic diagram of Cu‐TCPP@DIN@MPN nanopesticides. Reproduced with permission [118]. Copyright 2024, American Chemical Society. Created with Autodesk 3ds Max and Microsoft PowerPoint.

Porphyrin‐based porous materials with piezoelectric response have also been applied to alleviate osteomyelitis. For example, Tan et al. constructed a piezoelectric‐responsive heterostructure (PCN‐222‐BTO) by anchoring barium titanate (BTO) onto the surface of PCN‐222 via electrostatic interactions [116]. Osteomyelitis caused by MRSA is a severe bone infection that remains a significant clinical challenge to treat. Therefore, researchers have applied PCN‐222‐BTO to the treatment of osteomyelitis. PCN‐222‐BTO exists at an abiotic/abiotic interface between PCN‐222 and BTO, and upon bacterial contact, an abiotic/biotic interface forms between BTO and the bacterial membrane. Under ultrasound irradiation, the piezoelectric polarization of BTO generates an internal electric field that drives electrons from the excited PCN‐222 toward the BTO interface, significantly enhancing the bactericidal effect. Researchers have discovered that high concentrations of ROS can destroy cells, and low concentrations of ROS can act as signaling factors to regulate cell growth. Based on this, Tan et al. take advantage of this feature and innovatively introduce the Food and Drug Administration (FDA)‐approved drug alendronate (ALN) into PMOFs to synthesize a defect‐engineered PMOF sonosensitizer (HN25) with potent antibacterial properties and bone‐repairing capabilities. Remarkably, the addition of ALN not only exerts antimicrobial properties but also promotes bone targeting and inhibits osteoclast activity, while significantly enhancing sonodynamic capabilities. Osteogenic differentiation and repair can be achieved by different conditions of ultrasonic irradiation [95]. In three animal models: paravertebral infection, infected fractures, and osteomyelitis, HN25 demonstrated potent repair of infected bone tissue by rapidly eliminating MRSA, reducing osteoclast activity, and accelerating bone regeneration.

Wu et al. constructed Au NRs/Pt SAs co‐doped PMOF coated with red blood cell (RBC) membrane (RBC‐HNTM‐Pt@Au) (Figure 12b). Under ultrasound excitation, porphyrin structures generate electrons, and Pt SAs act as electron traps, restraining electron‐hole recombination and catalyzing the production of ^1^O_2_. Au NRs enhance the cavitation effect and increase ROS production synergistically. Experimental results indicate that after 15 min of ultrasonic irradiation, the system achieves 99.9% antibacterial efficacy against MRSA. The RBC coating endows the system with good biocompatibility and dynamic toxin neutralization ability, ultimately achieving efficient SDT of MRSA‐infected osteomyelitis [36]. This year, Zhang et al. synthesized porous PMOFs with varying Mn^2+^ doping levels. These PMOFs were physically adsorbed with CaO_2_ NPs to produce Mn_0.1_PCC composites [77]. Leveraging its exceptional microwave absorption properties, this material promotes CaO_2_ hydrolysis to produce H_2_O_2_ while mediating Mn^2+^ participation in the Fenton reaction to generate abundant ROS in situ. Concurrently, O_2_ release improves hypoxic conditions, establishing a self‐catalyzed O_2_‐producing microwave‐responsive system independent of external O_2_. Owing to its multifunctional characteristics, Mn_0.1_PCC demonstrates promising therapeutic efficacy against MRSA‐induced osteomyelitis.

#### 3.1.3 Treatment of Periodontitis

Periodontitis is a chronic inflammatory disease caused by bacterial infection, ranking as the sixth most common disease globally [183]. Currently, clinical treatment primarily relies on manual debridement and scaling combined with antibiotic therapy. However, traditional subgingival scaling is limited by the anatomical structure of deep periodontal pockets, making thorough debridement difficult [184]. Simultaneously, the increasing prevalence of microbial antibiotic resistance further complicates periodontitis treatment. Therefore, developing novel therapeutic strategies that precisely modulate the local dental microenvironment and enhance antimicrobial efficacy is urgently needed [185, 186, 187]. Notably, the local microenvironment at periodontal lesion sites exhibits characteristics such as high glucose expression, low pH, hypoxic conditions, and ROS enrichment. These features provide novel entry points and application opportunities for designing targeted antimicrobial treatment regimens.

Peng et al. developed a sequential therapeutic platform for periodontitis using a Zn‐based PMOF (PCN‐224) [188]. Upon 660 nm red light irradiation, PCN‐224 efficiently generates ^1^O_2_ and eradicates *Porphyromonas gingivalis* (*P. gingivalis*) with up to 99.9% efficiency within 5 min. Moreover, after being phagocytosed by macrophages, PCN‐224 exerts significant anti‐inflammatory and antioxidant effects, thereby effectively regulating immune responses. This work represents the first application of PCN‐224 in periodontitis treatment, establishing a sequential synergistic strategy of “antibacterial first, anti‐inflammatory later” and providing a novel, safe, and minimally invasive therapeutic approach for periodontal diseases. Wang et al. designed a Z‐scheme heterojunction composite (Bi_2_S_3_/Cu‐TCPP), which effectively broadens the light absorption range, significantly accelerates electron‐hole pair separation, and greatly improves ROS generation efficiency [123]. In this heterostructure, Bi_2_S_3_ provides outstanding PTT performance, which promotes the release of Cu^2+^ and initiates a Fenton‐like reaction. This design finally achieves triple‐modal synergistic therapy (PDT/PTT/CDT). In vivo experiments confirmed that Bi_2_S_3_/Cu‐TCPP can efficiently eradicate periodontal biofilms, reduce alveolar bone resorption, and accelerate periodontal tissue regeneration. Pt, as a noble metal, is used for doping PMOFs owing to its enzymatic activity [101]. In 2022, Tan et al. introduced Pt NPs into PMOFs to prepare PCN‐222‐Pt. Notably, the researchers pre‐simulated the adsorption, activation, and decomposition of PCN‐222‐M (Pt, Au, Cu, and Ru) using DFT calculations. Compared to PCN‐222, PCN‐222‐Pt has a larger O–O distance and more negative O_2_ adsorption energy, both of which suggest a weakened O–O bond. This indicates the highest oxidase and POD‐like activities of PCN‐222‐Pt. Leveraging endogenous O_2_ and overexpressed H_2_O_2_ within the microenvironment of periodontitis lesions, the dual‐enzyme‐active PCN‐222‐Pt serves as a ROS generator, enabling sustained ROS production without external light exposure. In vitro and in vivo experiments show that PCN‐222‐Pt exhibits anti‐biofilm properties against *E. coli* and *S. aureus*, and is utilized in periodontitis therapy when formulated with polyethylene glycol into an ointment. This not only provides the possibility of non‐invasive periodontitis treatment, but also offers a new idea for further research on MOF‐based SAs’ catalytic systems [38].

### 3.2 Antimicrobial Applications in Food and Agriculture

#### 3.2.1 Food Preservation

Porphyrin‐based porous materials combined with a drug can be applied not only for wound healing but also for food preservation [75, 138, 189]. Food safety is intrinsically linked to human health and well‐being, and food preservation serves as a critical component in ensuring food safety and extending shelf life. Bacterial and microbial contamination pose the foremost threat in food preservation, causing substantial economic losses while triggering severe food safety incidents and public health risks. Current mainstream preservation techniques include cold storage, modified atmosphere packaging, and chemical preservatives. However, these traditional methods often suffer from high costs, compromise inherent flavor, and leave chemical residues. Notably, photodynamic technology has gained widespread application in biomedicine due to its non‐invasive nature, high biocompatibility, and low toxicity. Porphyrin‐based porous materials, as representative carriers for this technology, have demonstrated promising prospects in preserving fruits, vegetables, meat, and other foods, attracting significant attention.

Liu et al. fabricated a novel donor‐acceptor structured Por‐Bp‐Cu COF via the solvothermal and in situ growth method [190]. Combining the CDT of Cu^2+^ in ligands with the PDT of Por‐Bp‐Cu COF, the researchers developed a high‐performance biosensor against *S. aureus*. Featuring a broad detection range, an ultra‐low limit of detection (12 CFU mL^−1^), and outstanding long‐term durability, this work significantly promotes the research and application of PCOFs in the field of food safety monitoring. Dong et al. designed a PC/GR‐MOF‐545 composite film by encapsulating geraniol (GR) into MOF‐545, further incorporated into a composite substrate composed of carboxymethyl chitosan and polyvinyl alcohol (Figure 12c). PC/GR‐MOF‐545 possesses excellent homogeneity and elongation at break, and can effectively mitigate food oxidation and alleviate photo oxidative damage to food. This membrane can be used to craft a biocompatible preservative film, distinguished by its impressive ability to preserve cherry tomatoes and prevent food oxidation. After 12 days of storage, moisture, firmness, and flavor retention were virtually identical to those of fresh cherry tomatoes. This study innovatively combines photodynamic and chemical antimicrobials to provide a novel approach for fruit and food preservation [35]. Moreover, Zhu et al. loaded the thymol (THY) into the pores of PCN‐224, then embedded it into the nanofiber membrane for use as an antimicrobial food packaging material (THY@PCN/PUL/PVA). Under light irradiation, this nanofiber membrane not only shows excellent photodynamic bactericidal effects, but also achieves sustained release of THY. Researchers also applied the preservation experiment with grapes and strawberries using this film; the results show that the fruits have no signs of spoilage, demonstrating its long‐lasting preservation effect [96]. Li et al. encapsulated oregano essential oil within porphyrin‐grafted quaternized chitosan to achieve sustained release of the oil [191]. Leveraging the acid‐producing nature of food spoilage processes, they blended pH‐responsive carbon quantum dots with this material to develop a smart packaging film that combines sustained release of antimicrobial agents with real‐time monitoring of food spoilage. Experimental results demonstrated that this packaging film extended the sustained release duration of oregano essential oil to 140 h while prolonging strawberry shelf life to 12 days.

Additionally, due to microbial growth, meat is prone to spoilage and deterioration. To address this problem, Wu et al. fabricated a PCN@PDA hybrid material with PTT and PDT, which was further embedded in a gelatin/carboxymethyl cellulose matrix to prepare an antimicrobial packaging film. Notably, this film can effectively inhibit microbial growth in pork by reducing the formation of amine compounds, thereby achieving efficient preservation and storage [107]. Moreover, researchers employed PDA as an intermediate layer to obtain CuBTC‑PDA‑TCPP, which was incorporated into a polyvinyl alcohol/silk‑fibroin matrix to create a light‑activated smart antibacterial PVA/SF‐MOF film suitable for preserving pork under refrigerated conditions [192]. Compared with MOF‐free films, PVA/SF‐MOF films can effectively block water‐vapor penetration pathways and improve water‐resistance performance. Owing to the excellent antibacterial activity of the film and an appropriate water‐vapor transmission rate, pork maintained favorable appearance and meat quality even after 8 days of storage. Notably, no Cu^2+^ migration was detected in pork samples packaged with PVA/SF‐MOF films, a result that also complied with relevant FDA specifications. In addition, cytotoxicity assays demonstrated that PVA/SF‐MOF films possessed low cytotoxicity toward mouse embryonic fibroblasts, laying a foundation for the safe application of PMOF‐based antibacterial materials in food preservation. Zhou et al. incorporated AgNPs‐modified PCN into the chitosan to prepare nanocomposite films (CS/PA) [193]. After systematic screening, when the PA loading is 0.6% *w*/w, the film shows a tensile strength of 48.75 MPa, an elongation at break of 23.12%, a decreased water‐vapor transmission rate, and preferable O_2_‐barrier properties. By harnessing the photocatalytic ROS generation of PCN, the constant Ag^+^ release, and the membrane‐disruptive capacity of CS, the CS/PA achieves efficient antibacterial properties. Given its lower Ag‐migration volume (33.02 µg L^−1^), the researchers further applied the film to fish fillets preservation, extending the shelf life to 12 days.

#### 3.2.2 Agricultural Disease Control

In the agricultural field, PMOFs also play a significant role [194]. Currently, the design of porphyrin‐based agro‐antimicrobials mainly relies on loading antimicrobial agents to achieve synergistic PDT and drug therapy. Xu et al. utilized the advantage of a two‐dimensional (2D) Cu‐TCPP layered structure to encapsulate diniconazole (DIN) on their surface with a metal‐phenolic network (MPN), constructing Cu‐TCPP@DIN@MPN nanopesticides (Figure 12d) [118]. The encapsulated DIN avoids premature loss and markedly improves bioavailability. Cu‐TCPP@DIN@MPN achieves efficient and long‐term control of plant pathogenic fungi by synergistically releasing DIN and Cu^2+^ while enhancing leaf adhesion with MPN. Cao et al. employed PCN‐224 as a carrier to load the fungicide tebuconazole (Tebuc), and fabricated pH/pectinase dual‐responsive microcapsules (Tebuc@PCN@P@C) via electrostatic layer‐by‐layer self‐assembly with pectin and chitosan. By combining the outstanding photodynamic antibacterial activity of PCN‐224 and the stimuli‐responsive release of Tebuc, this system exhibited excellent inhibitory efficacy against *Xanthomonas campestris pv. Campestris*, *Pseudomonas syringae pv. Tomato*, and *Alternaria alternata*. Meanwhile, the formulation showed favorable biosafety toward crops, providing a promising strategy for constructing eco‐friendly intelligent pesticide delivery systems [195]. Rice is a major global staple crop, but it is highly vulnerable to pathogen infection, causing rice sheath blight, which can lead to yield losses of up to 50%. To solve this problem, Wu et al. developed a smart pH/light dual‐responsive nanopesticide by loading the fungicide prochloraz (Pro) into Mg‐doped PMOFs and further coating it with PDA for targeted control of rice sheath blight (PCN‐222(Mg)@Pro@PDA) [196]. Mg doping significantly promoted charge separation and ROS generation under visible light irradiation, while the acidic microenvironment associated with pathogen infection triggered the rapid release of Pro. Antifungal assays demonstrated that the half‐maximal effective concentration of PCN‐222(Mg)@Pro@PDA against *Rhizoctonia solani* under light irradiation was 6.215 mg L^−1^, remarkably superior to the commercial formulation (9.33 mg L^−1^). Moreover, this nanoplatform exhibited enhanced rainfastness and improved biosafety, providing a promising strategy for the development of intelligent and eco‐friendly pesticide delivery systems.

### 3.3 Antimicrobial Applications in Functional Textiles and Wastewater Treatment

#### 3.3.1 The Fabrication of Sterilized Cotton Fabric

Recently, the development of antibacterial medical cotton fabrics has attracted increasing attention [121]. Zhao et al. synthesized Por‐COFs from Por 1 and L4 [197]. The resultant materials were subsequently blended with nylon 66 to fabricate Por‐COFs‐HP@PA66 interwoven textiles. Owing to ROS generated via type I and type II photodynamic mechanisms, the composite fabrics exhibited remarkable antibacterial activity against *S. aureus*, *E. coli*, *P. Aeruginosa*, and *S. epidermidis*. For sterilized cotton fabrics, recyclability after washing is a critical indicator for evaluating their stability. Por‐COFs‐HP@PA66 retained antibacterial rates over 80 % against *S. aureus* and *E. coli* after 16 washing cycles, which fully demonstrates its favorable reusability. Dong et al. synthesized an Ag@DhaTph‐COOH, which was characterized by chemo/PTT/PDT three‐modality synergistic therapy. Notably, it is mixed with polyurethane oligomers and sprayed onto the polyethylene terephthalate fabric surface to form self‐sanitizing face masks during the rapid spread of COVID‐19 respiratory infections. Furthermore, after five washing‐regeneration cycles, the mask maintains >95 % antibacterial and antiviral efficiencies, together with preserved structural integrity and crystallinity [136]. Wei et al. also in situ anchored PCN‐224 MOF and Ag NPs onto cotton fibers to construct KCT@PCN‐Ag, a visible‐light‐responsive PTT/PDT/Ag^+^ triple‐effect synergistic self‐disinfecting fabric. Notably, it achieves rapid and efficient sterilization against *S. aureus* and *E. coli*, maintaining a 6‐log bactericidal rate even after 10 washes, laying the foundation for a new generation of long‐lasting medical textiles [54]. In addition, Zang et al. constructed Ag_9_‐AgTPyP using a simple one‐pot method, which was characterized by a high surface area and enhanced light absorption ability. The Ag clusters of Ag_9_‐AgTPyP and the Ag atoms of the porphyrin rings show higher stability than the isolated Ag clusters. Notably, it can achieve almost 100% photocatalytic sterilization against MRSA and *P. aeruginosa*, even at a concentration of 50 µg mL^−1^ [90]. Notably, Ag_9_‐AgTPyP has also been used to fabricate microbial protective films for various applications, such as masks and biohazard suits. Even after three cycles of contamination‐light exposure‐cleaning, the antibacterial performance of the film showed no signs of decline.

#### 3.3.2 Wastewater Treatment and Purification

Bacterial contamination in wastewater poses persistent risks to environmental safety and public health. Porphyrin‐based porous materials combine porous structures, pollutant adsorption, and photoactive or catalytic antibacterial properties, making them promising materials for antibacterial wastewater treatment. Representative PMOFs, PCOFs, and PHOFs have demonstrated effective antibacterial performance in aqueous environments. For example, Dai et al. constructed a PDA coating on filter paper via dopamine self‐polymerization, followed by the in situ growth of Zr‐TCPP using a hydrothermal method to fabricate the Zr‐TCPP@PDA@FP composite membrane [72]. Porphyrin enhances visible‐light absorption, while PDA acts as an electron acceptor to facilitate the separation of photogenerated electron‐hole pairs in Zr‐TCPP; the two components synergistically boost photocatalytic activity. With favorable photothermal performance, the composite membrane achieves over 99% antibacterial efficiency against *E. coli* and *S. aureus* after 120  min of treatment. Meanwhile, it exhibits excellent photocatalytic reduction performance toward Cr (VI), attaining 93.9 % Cr (VI) removal within 20  min and possessing favorable cycling stability. This offers a new perspective for constructing highly efficient and sustainable photocatalytic materials for sewage purification. Sc^3+^ as a metal node to construct PMOFs, which is little reported. Specifically, Zheng et al. employed solution anode glow discharge (SAGD) to synthesize ScMPF with large particle sizes and superior crystal structure (SAGD‐ScMPFs) [57]. Notably, these methods provide an amount of electrons that not only accelerates the reaction rate but also promotes Sc^3+^ coordination with porphyrin rings. Abundant Sc^3+^ successfully suppresses electron‐hole recombination, laying the foundation for the production of ROS and reduced fluorescence background. Compared with PMOFs synthesized by solvothermal methods, photocurrent, and ROS yields, both achieve a several‐fold enhancement. Owing to its excellent oxidase‐like activity and photocatalytic performance, the material can efficiently inhibit *S. aureus*, *E. coli*, and *Salmonella* at ultra‐low concentrations, showing great potential for water disinfection.

PCOFs also have a significant effect on wastewater purification. Hu et al. designed a new COF (DmtaTph), which was designed by introducing methylthio units into pristine COF‐366 [39]. Focusing on wastewater treatment, methylthio units can adsorb Hg^2+^, and DmtaTph exhibits photo‐assisted antibacterial properties even under sunlight. This also demonstrates the potential and prospects in wastewater treatment. Dong et al. first synthesized α‐aminophosphonate‐linked PCOF (APCOF‐1) via the three‐component one‐pot in situ Kabachnik‐Fields reaction [198]. Accessible at room temperature under ambient air, this material supports gram‐scale synthesis. By integrating bioactive α‐aminophosphonate moieties and photosensitive porphyrin units in its framework, APCOF‐1 delivers excellent bactericidal activity against *E. coli* and *S. aureus* under natural sunlight irradiation. Its framework phosphonate groups serve as chelating sites to efficiently adsorb diverse heavy‐metal ions (Cd(II), Pb(II), Cu(II), Hg(II), Ni(II), Mn(II)). Furthermore, combined with chitosan, the resulting APCOF‐1@chitosan aerogel enables simultaneous bacterial inactivation (99.3%) and mixed heavy‐metal‐ion removal (99.9%) in sewage under sunlight, with recyclability for at least five cycles. This work also offers new insights into developing energy‐saving multifunctional PCOF materials for practical sewage‐purification scenarios. Wang et al. constructed a 3D HOF (JZS‐1) with one‐dimensional (1D) square channels based on a tetrazolyl‐functionalized porphyrin ligand. Upon loading methylene blue through host‐guest interactions, efficient fluorescence resonance energy transfer (FRET) occurs, which significantly boosts ^1^O_2_ generation and enables nearly 100% antibacterial efficiency under red‐light irradiation [151]. In summary, although porphyrin‐based porous materials demonstrate excellent antibacterial performance in wastewater treatment, the rational design of their structure and the optimization of their performance are still required [199]. Notably, most current studies still focus on model bacteria, while antibacterial selectivity, photostability, reusability, and long‐term performance in complex wastewater remain insufficiently investigated. In addition, natural organic matter, inorganic ions, and other ROS scavengers in wastewater may consume reactive species or reduce light transmission. Surface fouling and pore blocking may also decrease antibacterial performance. Therefore, future studies should focus on achieving stable and reproducible antibacterial activity under realistic wastewater conditions.

Overall, beyond direct porphyrin‐mediated photodynamic ROS generation, porous frameworks can also contribute to antibacterial activity through surface engineering for enhancing material‐bacteria interactions (e.g., electrostatic adsorption, boronic‐acid‐mediated targeting, and covalent bacterial capture), microenvironmental regulation (e.g., glucose consumption and hypoxia alleviation), gas therapy (e.g., NO), PTT, and the delivery of antimicrobial agents. These diverse functions make porphyrin‐based porous frameworks promising platforms for combining multiple antibacterial modalities to enhance antimicrobial effects.

## 4 Conclusion

In summary, this review summarizes the recent advances of porphyrin‐based porous materials (PMOFs, PCOFs, PHOFs, and APPPs) in the antibacterial fields of healthcare, food and agriculture, functional textiles, wastewater treatment, and at the laboratory stage. We also systematically summarize and discuss the construction strategies, different blocks, fabrication conditions, and antibacterial mechanisms. Porphyrins, as star molecules of PSs, have become ideal precursors for the construction of porphyrin‐based porous materials. First, pristine porphyrin‐based porous materials possess strong light absorption capacity. As promising PSs, they enable phototherapeutic antibacterial strategies to avoid the problem of bacterial drug resistance, which outperforms many conventional porous materials. Second, porphyrin‐based porous materials have good chemical stability, excellent surface areas, and pore structures that can effectively load drugs and improve stability and solubility, providing useful drug carriers. Third, porphyrin‐based porous material composites can realize the enhancement and integration of functions. After rational post‐synthetic modification, various materials such as MNPs, nanozymes, metal oxides, and polymers can all synthesize diverse hybrid materials by combining with porphyrin‐based porous materials. In conclusion, porphyrins as raw materials for the construction of porous materials to achieve antimicrobial properties have become a powerful strategy, and these materials have more advantages over conventional porous materials. Although porous antibacterial materials based on porphyrins have made much progress, there are many significant challenges for future practice.

- The current synthetic methods mainly include hydrothermal, solvent thermal, and microwave‐assisted synthesis. Among them, the most widely used solvent thermal method usually requires a high temperature for a long time, and the efficiency is low in the case of large‐volume preparation. Moreover, porphyrin‐based porous materials prepared by this method generally suffer from easy agglomeration, difficult dispersion, and a large size. Therefore, it is very necessary and important to design simpler, more efficient, greener, and milder synthetic methods. Therefore, it is very necessary and important to design simpler, more efficient, greener, milder, and scalable synthetic methods. In this regard, we can use the template method and the stripping method for the design of porous materials, which can not only facilitate the dispersion of materials and improve the controllability of morphology and size, but also effectively avoid aggregation. Moreover, current ROS studies mainly rely on solution‐phase probes and electron paramagnetic resonance, while pore‐scale and material‐bacteria interfacial imaging remains limited, which restricts the understanding of antimicrobial mechanisms and further improvement of antibacterial efficacy.
- Even though the compatibility of porphyrin‐based porous materials has been demonstrated in cellular experiments, their toxicity to organisms should be further investigated. Due to the diverse structure of porphyrin‐based porous materials and the complex microenvironment of organisms, it is urgent to study the long‐term safety and stability of porphyrin‐based porous materials, and the metabolic mechanism, products, and cycle of them in vivo should be specifically monitored. In this regard, we can consider the following three aspects. There are some known biocompatible metal ions, such as Zn^2+^ and Ca^2+^ can be employed as functional blocks to decrease the toxicity of porphyrin‐based porous materials. Some polymers can modify them to improve hydrophilicity, such as polyethylene glycol and chitosan. We can design more ligands that are biodegradable under physiological conditions and precisely control the structure of the material to achieve a controlled degradation rate.
- Currently, the antibacterial mechanisms of porphyrin‐based porous materials primarily focus on PDT, PTT, SDT, and synergistic antibacterial. However, photodynamic and photothermal conversion capabilities are limited; SDT also needs to be further explored. Therefore, we should also develop novel antibacterial mechanisms to enrich the antibacterial blueprint for porphyrin‐based porous materials. For the design of pristine materials, attention should be paid to the introduction of active sites and functional building blocks, which are important for the enhancement of antimicrobial capacity. For the construction of composite materials, there is an urgent need to further enrich the cascade catalysis, enhance light absorption, and advance the promotion of synergistic antimicrobial activity.
- The existing antibacterial experiments primarily focus on the inhibition of *S. aureus* and *E. coli*, while the actual bacterial microenvironment is more complex. Simultaneously, bacteria continue to evolve, leading to the emergence of increasingly resistant strains and challenging the antibacterial agents. Therefore, we can further broaden our ideas and horizons to study the inhibition effects of more resistant and clinically rare bacteria during the preliminary experiments. In addition, for specific applications such as antibacterial textiles, standardized washing and testing protocols are still lacking to systematically evaluate interfacial stability and long‐term antibacterial performance under practical conditions.
- Antibacterial selectivity remains an important challenge for porphyrin‐based porous materials. Although targeting units such as quaternary ammonium, boric acid, and mannosamine groups have been introduced to enhance bacterial binding, these strategies generally improve bacterial affinity rather than selectively recognize pathogens. Therefore, developing more specific recognition mechanisms based on pathogen‐associated markers or microenvironmental features is important for achieving selective pathogen elimination while minimizing effects on beneficial microorganisms.
- Finally, although many porphyrin‐based porous materials have been synthesized, the design and preparation of new porous materials are also highly emphasized by researchers. As the research fever of porphyrin‐based porous materials increases, it is very significant to explore more possibilities. Supramolecular chemistry has attracted a great deal of attention in recent years. The construction of porous materials through multiple non‐covalent interactions between porphyrins and supramolecules enables structural optimization and performance enhancement [200]. Additionally, our group has reported work that realizes the enhancement of photocatalytic and antimicrobial abilities in porous materials based on pillararenes. We can further employ computational methods, such as DFT, to perform simulations that assist in designing optimal structures and synthesis methods for these materials.

Overall, porphyrin‐based porous materials still face many challenges, and further advances are expected to provide some theoretical support and reference bases for further research. With the continuous updating of the structure of porphyrin‐based porous materials and in‐depth research, we have the belief that we will be able to provide a better choice for the treatment of bacterial infections. The development of porous materials supports the opportunity to achieve a great breakthrough, which will help us finish the mission of benefiting mankind.

## Author Contributions

**Xiao‐Xue Liang**: investigation, conceptualization, writing – original draft. **Xin Li**: writing – original draft. **Ying Xiong**: funding acquisition, resources, writing – review and editing. **Xin Wang**: conceptualization, supervision, funding acquisition, writing – original draft. **Ying‐Wei Yang**: conceptualization, investigation, supervision, funding acquisition, writing – review and editing, resources.

## Funding

Cultivation Project for Young and Middle‐aged Scientific and Technological Innovation Talents (Teams) of Jilin Province (20260601071RC), National Natural Science Foundation of China (Nos. 52403269, 51674131), Bethune Program of Jilin University (No. 2026B23), International industrial technology research and development project of Department of Science and Technology of Liaoning Province (2025JH2/101900019), Liaoning Revitalization Talents Program (XLYC2502057), and the Liaoning Young Elite Scientists Sponsorship Program, and Special Project for Scientific and Technological Talents of Shenyang, and Social Governance Technology Special Project of Shenyang (No. 24‐213‐3‐19).

## Conflicts of Interest

The authors declare no conflicts of interest.

## Data Availability

Data sharing not applicable to this article as no datasets were generated or analysed during the current study.

## References

[1] H. Hu , H. Wang , Y. Yang , J.‐F. Xu , and X. Zhang , “A Bacteria‐Responsive Porphyrin for Adaptable Photodynamic/Photothermal Therapy,” Angewandte Chemie International Edition 61, no. 23 (2022): 202200799, 10.1002/anie.202200799. 35332634

[2] X.‐R. Sun , J. Li , R.‐J. Chen , et al., “Engineering Porphyrin‐Derived Nanomaterials for Multimodal Antibacterial Therapy: from Rational Design to Practical Applications,” Coordination Chemistry Reviews 555 (2026): 217635, 10.1016/j.ccr.2026.217635.

[3] T.‐Y. Wang , X.‐Y. Zhu , and F.‐G. Wu , “Antibacterial Gas Therapy: Strategies, Advances, and Prospects,” Bioactive Materials 23 (2023): 129–155, 10.1016/j.bioactmat.2022.10.008. PMC9661653 36406249

[4] R. Li , T. Chen , and X. Pan , “Metal–Organic‐Framework‐Based Materials for Antimicrobial Applications,” ACS Nano 15, no. 3 (2021): 3808–3848, 10.1021/acsnano.0c09617. 33629585

[5] J. Wang , S. P. Teong , S. N. Riduan , et al., “Redox Active Zn@MOFs as Spontaneous Reactive Oxygen Species Releasing Antimicrobials,” Journal of the American Chemical Society 146, no. 1 (2024): 599–608, 10.1021/jacs.3c10411. 38109168

[6] A. Das , T. P. Mohammed , and M. Sankaralingam , “Biological Activity of Copper Porphyrins,” Coordination Chemistry Reviews 506 (2024): 215661, 10.1016/j.ccr.2024.215661.

[7] X. Li , M. Shen , J. Yang , L. Liu , and Y.‐W. Yang , “Pillararene‐Based Stimuli‐Responsive Supramolecular Delivery Systems for Cancer Therapy,” Advanced Materials 36, no. 16 (2024): 2313317, 10.1002/adma.202313317. 38206943

[8] J. Tian , B. Huang , L. Xia , Y. Zhu , and W. Zhang , “A H_2_S‐Generated Supramolecular Photosensitizer for Enhanced Photodynamic Antibacterial Infection and Relieving Inflammation,” Advanced Science 11, no. 9 (2024): 2305183, 10.1002/advs.202305183. PMC10916657 38095436

[9] H. Zhai , L. Lv , H. Ren , et al., “Multimodal Antibacterial Agents Based on Photodynamic Therapy and Photothermal Therapy: Principles, Design Strategies and Applications,” Chemical Society Reviews 55, no. 16 (2026): 8653–8700, 10.1039/d6cs00072j. 42453065

[10] T. Wu , Z. Xu , G. Yi , et al., “Microenvironment‐Responsive Nanozyme for all Stages of Infectious Chronic Wound Management through Macrophage Reprogramming,” Advanced Functional Materials 36, no. 44 (2026): 75099, 10.1002/adfm.75099.

[11] J. Yang , D. Dai , X. Lou , L. Ma , B. Wang , and Y.‐W. Yang , “Supramolecular Nanomaterials Based on Hollow Mesoporous Drug Carriers and Macrocycle‐Capped CuS Nanogates for Synergistic Chemo‐Photothermal Therapy,” Theranostics 10, no. 2 (2020): 615–629, 10.7150/thno.40066. PMC6929989 31903141

[12] X. Wang , X. Qin , Y. Liu , et al., “Plasmonic Supramolecular Nanozyme‐Based Bio‐Cockleburs for Synergistic Therapy of Infected Diabetic Wounds,” Advanced Materials 36, no. 49 (2024): 2411194, 10.1002/adma.202411194. 39444055

[13] J. M. Park , K.‐I. Hong , H. Lee , and W.‐D. Jang , “Bioinspired Applications of Porphyrin Derivatives,” Accounts of Chemical Research 54, no. 9 (2021): 2249–2260, 10.1021/acs.accounts.1c00114. 33891405

[14] J. Wang , X. Zhu , Y. Zhang , Z. Pang , C. Zhao , and F. Zhang , “Structural Design of Porous Organic Polymers to Mitigate π‐Stacking‐Induced Quenching in Porphyrin/Phthalocyanine Photosensitizers for Enhanced Antibacterial Activity,” RSC Advances 15, no. 56 (2025): 48604–48627, 10.1039/d5ra08102e. PMC12684879 41367684

[15] J. Yang , D. Dai , Z. Cai , et al., “MOF‐Based Multi‐Stimuli‐Responsive Supramolecular Nanoplatform Equipped with Macrocycle Nanovalves for Plant Growth Regulation,” Acta Biomaterialia 134 (2021): 664–673, 10.1016/j.actbio.2021.07.050. 34329784

[16] H. Zhao , G. Shu , J. Zhu , Y. Fu , Z. Gu , and D. Yang , “Persistent Luminescent Metal‐Organic Frameworks With Long‐Lasting Near Infrared Emission for Tumor Site Activated Imaging and Drug Delivery,” Biomaterials 217 (2019): 119332, 10.1016/j.biomaterials.2019.119332. 31284124

[17] Y. Tian , B. Wang , Z. Zhang , T. Kai , P. Wu , and P. Ding , “Recent Advances in Metal–organic Frameworks for Antibacterial Applications: Mechanisms and Emerging Strategies,” RSC Advances 15, no. 33 (2025): 26710–26727, 10.1039/d5ra02955d. PMC12302127 40727299

[18] X. Zhang , F. Peng , and D. Wang , “MOFs and MOF‐Derived Materials for Antibacterial Application,” Journal of Functional Biomaterials 13, no. 4 (2022): 215, 10.3390/jfb13040215. PMC9680240 36412856

[19] M. Shen , F. Forghani , X. Kong , et al., “Antibacterial Applications of Metal–Organic Frameworks and Their Composites,” Comprehensive Reviews in Food Science and Food Safety 19, no. 4 (2020): 1397–1419, 10.1111/1541-4337.12515. 33337086

[20] L. Tabrizi , D. F. Hughes , and M. T. Pryce , “Covalent Organic Frameworks: Advancing Antimicrobial Photodynamic Therapy for Next‐Generation Treatments,” Coordination Chemistry Reviews 528 (2025): 216424, 10.1016/j.ccr.2024.216424.

[21] A. Schlachter , P. Asselin , and P. D. Harvey , “Porphyrin‐Containing MOFs and COFs as Heterogeneous Photosensitizers for Singlet Oxygen‐Based Antimicrobial Nanodevices,” ACS Applied Materials & Interfaces 13, no. 23 (2021): 26651–26672, 10.1021/acsami.1c05234. 34086450

[22] N. Hassanzadeh Goji , M. Ramezani , A. S. Saljooghi , and M. Alibolandi , “Porphyrin‐Based Metal–Organic Frameworks: Focus on Diagnostic and Therapeutic Applications,” Journal of Nanostructure in Chemistry 14, no. 2 (2024): 167–208, 10.1007/s40097-022-00500-6.

[23] L.‐J. Chen , X. Zhao , and X.‐P. Yan , “Porphyrinic Metal‐Organic Frameworks for Biological Applications,” Advanced Sensor and Energy Materials 2, no. 1 (2023): 100045, 10.1016/j.asems.2022.100045.

[24] X.‐G. Li , J. Li , J. Chen , et al., “Porphyrin‐Based Covalent Organic Frameworks from Design, Synthesis to Biological Applications,” Biomaterials Science 12, no. 11 (2024): 2766–2785, 10.1039/d4bm00214h. 38717456

[25] T. Li , W. Sun , H. Pan , et al., “Advancing Biomedical Technology through Multifunctional Porphyrin‐Based MOFs: Design Principles, Applications, and Biosafety Evaluations,” Materials Today Bio 37 (2026): 103025, 10.1016/j.mtbio.2026.103025. PMC13018993 41907112

[26] X. Wang , X. Wang , S. Wang , et al., “Progress in Porphyrin‐Based Covalent Organic Frameworks for Enhanced Photodynamic Antimicrobial Therapy,” Chemical Communications 61, no. 80 (2025): 15537–15560, 10.1039/d5cc04127a. 40955550

[27] C. Qi , M. Chen , Z. Yuan , W. Wang , and X. Zheng , “Phototherapeutic Antibacterial Applications of Porphyrin‐Based Metal–Organic Frameworks,” Chemical Communications 61, no. 79 (2025): 15313–15328, 10.1039/d5cc04455c. 40928219

[28] Y. Gao , Y. Li , Z. Xu , S. Yu , J. Liu , and H. Sun , “Multiporphyrinic Architectures: Advances in Structural Design for Photodynamic Therapy,” Aggregate 5, no. 1 (2024): 420, 10.1002/agt2.420.

[29] D. Xu , Q. Duan , H. Yu , and W. Dong , “Photodynamic Therapy Based on Porphyrin‐based Metal–organic Frameworks,” Journal of Materials Chemistry B 11, no. 26 (2023): 5976–5989, 10.1039/d2tb02789e. 37310273

[30] C. Qi , J. Chen , Y. Qu , X. Luo , W. Wang , and X. Zheng , “Recent Advances in Porphyrin‐Based Covalent Organic Frameworks for Synergistic Photodynamic and Photothermal Therapy,” Pharmaceutics 16, no. 12 (2024): 1625, 10.3390/pharmaceutics16121625. PMC11678282 39771603

[31] I. O. Savelyeva , K. A. Zhdanova , M. A. Gradova , O. V. Gradov , and N. A. Bragina , “Cationic Porphyrins as Antimicrobial and Antiviral Agents in Photodynamic Therapy,” Current Issues in Molecular Biology 45, no. 12 (2023): 9793–9822, 10.3390/cimb45120612. PMC10741785 38132458

[32] S. Chen , B. Huang , J. Tian , and W. Zhang , “Advancements of Porphyrin‐Derived Nanomaterials for Antibacterial Photodynamic Therapy and Biofilm Eradication,” Advanced Healthcare Materials 13, no. 27 (2024): 2401211, 10.1002/adhm.202401211. 39073000

[33] M. C. S. Vallejo , N. M. M. Moura , A. T. P. C. Gomes , et al., “The Role of Porphyrinoid Photosensitizers for Skin Wound Healing,” International Journal of Molecular Sciences 22, no. 8 (2021): 4121, 10.3390/ijms22084121. PMC8072979 33923523

[34] Y. Li , Q. Zhang , B. Duan , et al., “Near‐Infrared Light Driven Photodynamic Therapy by Hyaluronic Acid Encapsulated Ionic Polymer Integrated with Oxygen Self‐Supply and High Acetate Supplement for Chronic Wound Healing,” International Journal of Biological Macromolecules 314 (2025): 144424, 10.1016/j.ijbiomac.2025.144424. 40403517

[35] H. Bai , L. Yang , L. Wu , D. Xiao , and A. Dong , “Enhanced Food Preservation Platform Integrating Photodynamic and Chemical Antibacterial Strategies via Geraniol‐Loaded Porphyrin‐Based MOFs for Cherry Tomato Storage,” Chemical Engineering Journal 498 (2024): 155503, 10.1016/j.cej.2024.155503.

[36] Y. Yu , L. Tan , Z. Li , et al., “Single‐Atom Catalysis for Efficient Sonodynamic Therapy of Methicillin‐Resistant Staphylococcus Aureus‐Infected Osteomyelitis,” ACS Nano 15, no. 6 (2021): 10628–10639, 10.1021/acsnano.1c03424. 34086433

[37] X. Feng , L. Ma , J. Lei , et al., “Piezo‐Augmented Sonosensitizer with Strong Ultrasound‐Propelling Ability for Efficient Treatment of Osteomyelitis,” ACS Nano 16, no. 2 (2022): 2546–2557, 10.1021/acsnano.1c09189. 35044741

[38] Y. Yu , Y. Cheng , L. Tan , et al., “Theory‐Screened MOF‐Based Single‐Atom Catalysts for Facile and Effective Therapy of Biofilm‐Induced Periodontitis,” Chemical Engineering Journal 431 (2022): 133279, 10.1016/j.cej.2021.133279.

[39] M. Zhao , F. Zhao , J. Ping , et al., “A Recyclable Covalent Organic Framework for Selective Removal of Hg(II) and Sunlight‐Driven Sterilization in Water,” Chinese Chemical Letters 36, no. 10 (2025): 110782, 10.1016/j.cclet.2024.110782.

[40] X. Li , C. Li , and Y.‐W. Yang , “Noncovalent Interaction‐Mediated Metal‐Organic Framework‐Based Theranostic Systems for Precision Medicine,” Chinese Journal of Chemistry 44, no. 15 (2026): 2606–2624, 10.1002/cjoc.70608.

[41] H. Li , M. Eddaoudi , M. O'Keeffe , and O. M. Yaghi , “Design and Synthesis of an Exceptionally Stable and Highly Porous Metal‐ Organic Framework,” Nature 402, no. 6759 (1999): 276–279, 10.1038/46248.

[42] K. S. Park , Z. Ni , A. P. Côté , et al., “Exceptional Chemical and Thermal Stability of Zeolitic Imidazolate Frameworks,” Proceedings of the National Academy of Sciences 103, no. 27 (2006): 10186–10191, 10.1073/pnas.0602439103. PMC1502432 16798880

[43] J. H. Cavka , S. Jakobsen , U. Olsbye , et al., “A New Zirconium Inorganic Building Brick Forming Metal Organic Frameworks with Exceptional Stability,” Journal of the American Chemical Society 130, no. 42 (2008): 13850–13851, 10.1021/ja8057953. 18817383

[44] G. Férey , C. Mellot‐Draznieks , C. Serre , et al., “A Chromium Terephthalate‐Based Solid with Unusually Large Pore Volumes and Surface Area,” Science 309, no. 5743 (2005): 2040–2042, 10.1126/science.1116275. 16179475

[45] S. S. Y. Chui , S. M. F. Lo , J. P. H. Charmant , A. G. Orpen , and I. D. Williams , “A Chemically Functionalizable Nanoporous Material [Cu_3_( TMA)_2_(H_2_O)_3_]_n_ ,” Science 283 (1999): 1148–1150, 10.1126/science.283.5405.1148. 10024237

[46] S. Wan , F. Gándara , A. Asano , et al., “Covalent Organic Frameworks With High Charge Carrier Mobility,” Chemistry of Materials 23, no. 18 (2011): 4094–4097, 10.1021/cm201140r.

[47] D. Yu , H. Zhang , J. Ren , and X. Qu , “Hydrogen‐Bonded Organic Frameworks: New Horizons in Biomedical Applications,” Chemical Society Reviews 52, no. 21 (2023): 7504–7523, 10.1039/d3cs00408b. 37814831

[48] D. J. Duchamp and R. E. Marsh , “The Crystal Structure of Trimesic Acid (Benzene‐1,3,5‐Tricarboxylic Acid)” Acta Crystallographica Section B B25, no. 1 (1969): 5–19, 10.1107/S0567740869001713.

[49] Y. He , S. Xiang , and B. Chen , “A Microporous Hydrogen‐Bonded Organic Framework for Highly Selective C_2_H_2_/C_2_H_4_ Separation at Ambient Temperature,” Journal of the American Chemical Society 133, no. 37 (2011): 14570–14573, 10.1021/ja2066016. 21863860

[50] W. Yang , B. Li , H. Wang , et al., “A Microporous Porphyrin‐Based Hydrogen‐Bonded Organic Framework for Gas Separation,” Crystal Growth & Design 15, no. 4 (2015): 2000–2004, 10.1021/acs.cgd.5b00147.

[51] D. Han , Y. Li , X. Liu , et al., “Photothermy‐Strengthened Photocatalytic Activity of Polydopamine‐Modified Metal‐Organic Frameworks for Rapid Therapy of Bacteria‐infected Wounds,” Journal of Materials Science & Technology 62 (2021): 83–95, 10.1016/j.jmst.2020.05.055.

[52] X. Wang , W. Hu , X.‐H. Xia , and C. Wang , “Implanting of Single Zinc Sites into 2D Metal–Organic Framework Nanozymes for Boosted Antibiofilm Therapy,” Advanced Functional Materials 33, no. 10 (2023): 2212798, 10.1002/adfm.202212798.

[53] H.‐X. Feng , Z. Zhou , J. Jiang , et al., “A Porphyrin‐Based Metal–Organic Framework Nanozyme with Superior Peroxidase‐like Activity for Combating Antibacterial Infections and Promoting Wound Healing,” Inorganic Chemistry 64, no. 7 (2025): 3541–3552, 10.1021/acs.inorgchem.4c05258. 39930626

[54] X. Nie , S. Wu , S. Liao , et al., “Light‐Driven Self‐Disinfecting Textiles Functionalized by PCN‐224 and Ag Nanoparticles,” Journal of Hazardous Materials 416 (2021): 125786, 10.1016/j.jhazmat.2021.125786. 33873032

[55] M. Chen , Z. Long , R. Dong , et al., “Titanium Incorporation into Zr‐Porphyrinic Metal–Organic Frameworks with Enhanced Antibacterial Activity against Multidrug‐Resistant Pathogens,” Small 16, no. 7 (2020): 1906240, 10.1002/smll.201906240. 31967726

[56] L. Zhang , M. Ouyang , Y. Zhang , et al., “The Fluorescence Imaging and Precise Suppression of Bacterial Infections in Chronic Wounds by Porphyrin‐based Metal–organic Framework Nanorods,” Journal of Materials Chemistry B 9, no. 38 (2021): 8048–8055, 10.1039/d1tb01649k. 34486642

[57] Z. Zhang , Z. Liu , X. Chen , et al., “Plasma–Liquid‐Induced Synthesis of Scandium–Metalloporphyrin Frameworks for Boosted Sensing and Photosensitization,” Advanced Materials 37, no. 8 (2025): 2412071, 10.1002/adma.202412071. 39757435

[58] F. Zhou , J. Xu , Y. Du , et al., “An Enhanced Sonodynamic Antibacterial Strategy Using Bismuth Porphyrin Metal‐organic Frameworks to Inhibit Bacterial Endogenous Antioxidant H_2_S Metabolism,” Journal of Nanobiotechnology 24, no. 1 (2026): 548, 10.1186/s12951-026-04437-8. PMC13255331 42035137

[59] X. Sun , G. He , C. Xiong , et al., “One‐Pot Fabrication of Hollow Porphyrinic MOF Nanoparticles With Ultrahigh Drug Loading Toward Controlled Delivery and Synergistic Cancer Therapy,” ACS Applied Materials & Interfaces 13, no. 3 (2021): 3679–3693, 10.1021/acsami.0c20617. 33464038

[60] C.‐M. Zeng , S.‐Y. Luo , X. Wang , et al., “A Porphyrin‐Based 3D Metal‐Organic Framework Featuring [Cu_8_Cl_6_]^10+^ Cluster Secondary Building Units: Synthesis, Structure Elucidation, Anion Exchange, and Peroxidase‐like Activity,” Chemistry – An Asian Journal 19, no. 10 (2024): 202400237, 10.1002/asia.202400237. 38563626

[61] J. Xu , Z. Ding , M. Wang , G. Wu , and J. Xie , “Enhanced Stable and Efficient of Dual‐Ligand Zirconium‐Based Metal‐Organic Frameworks for Synergistic Photodynamic Inactivation,” Small 20, no. 49 (2024): 2406171, 10.1002/smll.202406171. 39258347

[62] Z.‐H. Zhu , Y.‐L. Li , H.‐L. Wang , H.‐H. Zou , F.‐P. Liang , and L. Zhou , “Designing Pillar–Layered Metal–Organic Frameworks With Photo‐Induced Electron Transfer Interactions Between Ligands for Enhanced Photodynamic Sterilization and Photocatalytic Degradation of Dyes and Antibiotics,” Journal of Colloid and Interface Science 685 (2025): 458–467, 10.1016/j.jcis.2025.01.148. 39855091

[63] L. Zhang , G. Ng , N. Kapoor‐Kaushik , et al., “2D Porphyrinic Metal–Organic Framework Nanosheets as Multidimensional Photocatalysts for Functional Materials,” Angewandte Chemie International Edition 60, no. 42 (2021): 22664–22671, 10.1002/anie.202107457. 34322965

[64] J. Xu , J. Zhu , J. Li , G. Weng , and J. Zhao , “Porphyrin‐Noble Metal Nanomaterial Synergistic Dance: Orchestrating Biomedical Sensing Breakthroughs & Therapeutic Frontiers,” Coordination Chemistry Reviews 546 (2026): 217078, 10.1016/j.ccr.2025.217078.

[65] P. Mroz , J. Bhaumik , D. K. Dogutan , et al., “Imidazole Metalloporphyrins as Photosensitizers for Photodynamic Therapy: Role of Molecular Charge, Central Metal and Hydroxyl Radical Production,” Cancer Letters 282, no. 1 (2009): 63–76, 10.1016/j.canlet.2009.02.054. PMC2782654 19346065

[66] W. Zhen , D. W. Kang , Y. Fan , et al., “Simultaneous Protonation and Metalation of a Porphyrin Covalent Organic Framework Enhance Photodynamic Therapy,” Journal of the American Chemical Society 146, no. 24 (2024): 16609–16618, 10.1021/jacs.4c03519. 38837955

[67] B. Bora , N. Das , J. P. Sultana , M. K. Raza , and T. K. Goswami , “Mn(III) Porphyrins as Photosensitizers: Structural, Photophysical and Anticancer Studies,” Dalton Transactions 54, no. 30 (2025): 11743–11756, 10.1039/d4dt03432e. 40673971

[68] Z.‐W. Zhou , C.‐X. Liu , C.‐X. Cai , et al., “Multiple Strategy for Antibacterial Effect via Copper Porphyrin Doping Polyphenolic Aldehydes Based Covalent Organic Framework,” Journal of Environmental Chemical Engineering 12, no. 5 (2024): 113533, 10.1016/j.jece.2024.113533.

[69] L. Zhang , F. Gao , S. Liu , et al., “Synthesis of Lanthanide‐Based Scintillator@MOF Nanocomposites for X‐Ray‐Induced Photodynamic Therapy,” Inorganic Chemistry Frontiers 11, no. 5 (2024): 1607–1615, 10.1039/d3qi02301j.

[70] W. Liu , Y. Hou , P. Zhao , Y. Shen , Y. Zhang , and C. D'Agostino , “Multifunctional Self‐Cleaning Zr‐Porphyrin@PG Membrane for Wastewater Treatment,” Applied Surface Science 687 (2025): 162290, 10.1016/j.apsusc.2025.162290.

[71] Y. Shi , Y. Wang , J. Bi , et al., “Surface Engineering for Ultrapermeable Antibacterial Conjugated Microporous Polymer‐Polyamide Composite Membranes,” Science China Materials 66, no. 8 (2023): 3319–3326, 10.1007/s40843-023-2476-5.

[72] C. Duan , S. Che , G. Tian , Y. Che , X. Nie , and L. Dai , “Mussel‐Inspired Polydopamine and Zr‐Porphyrin MOF Co‐Decoration of a Cellulose Paper‐Based Composite Membrane for Efficient Photoreduction of Cr(VI) and Photothermal Antibacterial in Wastewater,” Separation and Purification Technology 353 (2025): 128255, 10.1016/j.seppur.2024.128255.

[73] L. Yang , Y. Li , M. Xu , et al., “Ultrasound‐Activated Nanoprobiotic Hydrogel Disrupts the Oral‐Brain Axis to Alleviate Diabetic Periodontitis and Cognitive Decline,” Advanced Functional Materials 36, no. 10 (2025): 11408, 10.1002/adfm.202511408.

[74] X. Nie , S. Wu , T. Hussain , and Q. Wei , “PCN‐224 Nanoparticle/Polyacrylonitrile Nanofiber Membrane for Light‐Driven Bacterial Inactivation,” Nanomaterials 11, no. 12 (2021): 3162, 10.3390/nano11123162. PMC8707920 34947511

[75] Z.‐A. Deng , M. Wu , C. Shen , et al., “Microfluidic‐blow‐spinning of Carvacrol‐loaded Porphyrin Metal − Organic Framework Nanofiber Films with Synergistic Antibacterial Capabilities for Food Packaging,” Food Chemistry 460 (2024): 140707, 10.1016/j.foodchem.2024.140707. 39111037

[76] L.‐J. Chen , Y.‐Y. Liu , X. Zhao , and X.‐P. Yan , “Vancomycin‐Functionalized Porphyrinic Metal‐Organic Framework PCN‐224 with Enhanced Antibacterial Activity against Staphylococcus Aureus,” Chemistry – An Asian Journal 16, no. 15 (2021): 2022–2026, 10.1002/asia.202100546. 34096181

[77] H. Cheng , Y. Yang , Y. Wang , et al., “Microwave‐Responsive Nanoplatform with Robust ROS Generation and Inhibiting Effects of Two‐Component System and Quorum Sensing for the Treatment of MRSA‐Induced Osteomyelitis,” Advanced Functional Materials 34, no. 40 (2024): 2405421, 10.1002/adfm.202405421.

[78] J. Shang , R. Zhao , X. Sun , et al., “Enhanced Bacterial Disinfection of Multi‐Drug Resistance Bacteria by PCN@AgI Heterojunction under Visible Light Irradiation,” Chemical Engineering Journal 499 (2024): 155791, 10.1016/j.cej.2024.155791.

[79] H. Qiu , F. Pu , Z. Liu , et al., “Depriving Bacterial Adhesion‐Related Molecule to Inhibit Biofilm Formation Using CeO_2_ ‐Decorated Metal‐Organic Frameworks,” Small 15, no. 36 (2019): 1902522, 10.1002/smll.201902522. 31328358

[80] N. G. Bastús , F. Merkoçi , J. Piella , and V. Puntes , “Synthesis of Highly Monodisperse Citrate‐Stabilized Silver Nanoparticles of up to 200 Nm: Kinetic Control and Catalytic Properties,” Chemistry of Materials 26, no. 9 (2014): 2836–2846, 10.1021/cm500316k.

[81] X. Tan , S. Chen , D. Ming , G. Lv , B. Shen , and Y. Yang , “Near‐Infrared Light Triggered Photodynamic Therapy and Release of Silver Ion from CuTCPP Nanosheet for Synergistic Gram‐Positive Bacteria Elimination,” Journal of Solid State Chemistry 313 (2022): 123311, 10.1016/j.jssc.2022.123311.

[82] B.‐X. Xie , H.‐S. Wang , H.‐Q. Zheng , et al., “Boosting Antibacterial Photodynamic Therapy in a Nanosized Zr MOF by the Combination of Ag NP Encapsulation and Porphyrin Doping,” Inorganic Chemistry 62, no. 34 (2023): 13892–13901, 10.1021/acs.inorgchem.3c01785. 37587720

[83] K. Mao , Y. Zhu , J. Rong , et al., “Rugby‐Ball like Ag Modified Zirconium Porphyrin Metal–Organic Frameworks Nanohybrid for Antimicrobial Activity: Synergistic Effect for Significantly Enhancing Photoactivation Capacity,” Colloids and Surfaces A: Physicochemical and Engineering Aspects 611 (2021): 125888, 10.1016/j.colsurfa.2020.125888.

[84] G. Zhang , G. Zhang , H. Zhang , et al., “Enhanced Reactive Oxygen Species Production for Antibacterial Treatment with Ultrasound‐Activated 2D Mxene‐Porphyrin MOF Composite,” Applied Surface Science 688 (2025): 162327, 10.1016/j.apsusc.2025.162327.

[85] Y. Han , H. Zeng , H. Hao , et al., “Self‐Driven Charge Transfer Mechanism of Bi NPs/PCN‐224 for Enhanced Photodynamic Antimicrobial Chemotherapy Effect,” Journal of Colloid and Interface Science 689 (2025): 137207, 10.1016/j.jcis.2025.02.215. 40054254

[86] X. Guo , B. Guo , Y. Wang , and S. Guan , “Preparation of Spherical Metal–organic Frameworks Encapsulating Ag Nanoparticles and Study on Its Antibacterial Activity,” Materials Science and Engineering: C 80 (2017): 698–707, 10.1016/j.msec.2017.07.027. 28866218

[87] X. Zhao , L. Chang , Y. Hu , et al., “Preparation of Photocatalytic and Antibacterial MOF Nanozyme Used for Infected Diabetic Wound Healing,” ACS Applied Materials & Interfaces 14, no. 16 (2022): 18194–18208, 10.1021/acsami.2c03001. 35412791

[88] Y. Chen , D. Li , Y. Zhong , Z. Lu , and D. Wang , “NIR Regulated Upconversion Nanoparticles@Metal‐organic Framework Composite Hydrogel Dressing with Catalase‐like Performance and Enhanced Antibacterial Efficacy for Accelerating Wound Healing,” International Journal of Biological Macromolecules 235 (2023): 123683, 10.1016/j.ijbiomac.2023.123683. 36806777

[89] Y. Luo , J. Li , X. Liu , et al., “Dual Metal–Organic Framework Heterointerface,” ACS Central Science 5, no. 9 (2019): 1591–1601, 10.1021/acscentsci.9b00639. PMC6764158 31572786

[90] M. Cao , S. Wang , J.‐H. Hu , B.‐H. Lu , Q.‐Y. Wang , and S.‐Q. Zang , “Silver Cluster‐Porphyrin‐Assembled Materials as Advanced Bioprotective Materials for Combating Superbacteria,” Advanced Science 9, no. 2 (2022): 2103721, 10.1002/advs.202103721. PMC8805555 34761563

[91] B. Xue , X. Geng , H. Cui , et al., “Size Engineering of 2D MOF Nanosheets for Enhanced Photodynamic Antimicrobial Therapy,” Chinese Chemical Letters 34, no. 9 (2023): 108140, 10.1016/j.cclet.2023.108140.

[92] Y. Zeng , Q. Ouyang , Y. Yu , et al., “Defective Homojunction Porphyrin‐Based Metal‐Organic Frameworks for Highly Efficient Sonodynamic Therapy,” Small Methods 7, no. 1 (2023): 2201248, 10.1002/smtd.202201248. 36549891

[93] M. Chen , J. Zhang , J. Qi , et al., “Boronic Acid‐Decorated Multivariate Photosensitive Metal–Organic Frameworks for Combating Multi‐Drug‐Resistant Bacteria,” ACS Nano 16, no. 5 (2022): 7732–7744, 10.1021/acsnano.1c11613. 35535857

[94] Y. Zeng , C. Wang , K. Lei , et al., “Multifunctional MOF‐Based Microneedle Patch with Synergistic Chemo‐Photodynamic Antibacterial Effect and Sustained Release of Growth Factor for Chronic Wound Healing,” Advanced Healthcare Materials 12, no. 19 (2023): 2300250, 10.1002/adhm.202300250. 36940484

[95] L. Ma , Y. Cheng , X. Feng , et al., “A Janus‐ROS Healing System Promoting Infectious Bone Regeneration via Sono‐Epigenetic Modulation,” Advanced Materials 36, no. 2 (2024): 2307846, 10.1002/adma.202307846. 37855420

[96] T. Min , X. Sun , L. Zhou , H. Du , Z. Zhu , and Y. Wen , “Electrospun Pullulan/PVA Nanofibers Integrated with Thymol‐Loaded Porphyrin Metal−Organic Framework for Antibacterial Food Packaging,” Carbohydrate Polymers 270 (2021): 118391, 10.1016/j.carbpol.2021.118391. 34364632

[97] M. Zhang , J. Cheng , Z. Shen , K. He , and B. Zheng , “Red Light‐Triggered Release of ROS and Carbon Monoxide for Combinational Antibacterial Application,” Journal of Materials Chemistry B 12, no. 4 (2024): 1077–1086, 10.1039/d3tb01829f. 38168810

[98] D. Han , Y. Han , J. Li , et al., “Enhanced Photocatalytic Activity and Photothermal Effects of Cu‐doped Metal‐Organic Frameworks for Rapid Treatment of Bacteria‐Infected Wounds,” Applied Catalysis B: Environmental 261 (2020): 118248, 10.1016/j.apcatb.2019.118248.

[99] L. Lai , Q. Guo , W. Zou , et al., “Space‐Confined Nanozyme with Cascade Reaction Based on PCN‐224 for Synergistic Bacterial Infection Treatment and NIR Fluorescence Imaging of Wound Bacterial Infections,” Chemical Engineering Journal 487 (2024): 150642, 10.1016/j.cej.2024.150642.

[100] S. Sun , X. Meng , J. Xu , Z. Yang , X. Zhang , and H. Dong , “Ag‐Decorated Multivariate Metal‐organic Frameworks for Enhanced Sonodynamic Therapy of Bacterial Infections in Wound Healing,” Nano Today 55 (2024): 102181, 10.1016/j.nantod.2024.102181.

[101] F. Dong , Y. Liu , H. Dong , et al., “A Nano‐Oxygen Generator for Enhanced Light‐Controlled Elimination of Anaerobic Bacteria in Periodontal Infections,” Nano Today 56 (2024): 102280, 10.1016/j.nantod.2024.102280.

[102] J. Sun , Y. Fan , W. Ye , et al., “Near‐Infrared Light Triggered Photodynamic and Nitric Oxide Synergistic Antibacterial Nanocomposite Membrane,” Chemical Engineering Journal 417 (2021): 128049, 10.1016/j.cej.2020.128049.

[103] X. Zhang , Z. Wang , Q. Liu , et al., “Targeted Modulation and Enhancement of Macrophages via Sonodynamic Therapy‐Driven Cupferroptosis‐Like Stress for Implant‐Associated Biofilm Infections,” Nano Today 54 (2024): 102092, 10.1016/j.nantod.2023.102092.

[104] Y. Luo , X. Liu , L. Tan , et al., “Enhanced Photocatalytic and Photothermal Properties of Ecofriendly Metal‐organic Framework Heterojunction for Rapid Sterilization,” Chemical Engineering Journal 405 (2021): 126730, 10.1016/j.cej.2020.126730.

[105] W. Sun , J. Sun , Q. Ding , et al., “Breaking Iron Homeostasis: Iron Capturing Nanocomposites for Combating Bacterial Biofilm,” Angewandte Chemie International Edition 63, no. 14 (2024): 202319690, 10.1002/anie.202319690. 38320965

[106] B. Xu , L. Wang , Z. Shen , B. Yan , D. Qi , and J. Wu , “Construction of Acidic Microenvironment by Cu‐TCPP Nanozyme Composited Deprotonatable Polymeric Nanofibers for Efficient Antibacterial Activity,” Chemical Engineering Journal 501 (2024): 157594, 10.1016/j.cej.2024.157594.

[107] H. Yan , X. Lin , N. Duan , I. M. Khan , Z. Wang , and S. Wu , “Gelatin‒Carboxymethyl Cellulose Film Incorporated Porphyrin Metal–Organic Frameworks with Photodynamic/Photothermal Synergistic Antibacterial Activities for Pork Packaging Application,” Journal of Food Engineering 353 (2023): 111556, 10.1016/j.jfoodeng.2023.111556.

[108] Q. Deng , P. Sun , L. Zhang , et al., “Porphyrin MOF Dots–Based, Function‐Adaptive Nanoplatform for Enhanced Penetration and Photodynamic Eradication of Bacterial Biofilms,” Advanced Functional Materials 29, no. 30 (2019): 1903018, 10.1002/adfm.201903018.

[109] C. Tan , X. Li , Z. Li , et al., “Near‐Infrared‐Responsive Nanoplatforms Integrating Dye‐Sensitized Upconversion and Heavy‐Atom Effect for Enhanced Photodynamic Therapy Efficacy,” Nano Today 54 (2024): 102089, 10.1016/j.nantod.2023.102089.

[110] Y.‐J. Wei , H. Chen , Z.‐W. Zhou , et al., “Kill Two Birds with One Stone: Dual‐Metal MOF‐Nanozyme‐Decorated Hydrogels with ROS‐Scavenging, Oxygen‐Generating, and Antibacterial Abilities for Accelerating Infected Diabetic Wound Healing,” Small 20, no. 48 (2024): 2403679, 10.1002/smll.202403679. 39240068

[111] X. Lv , J. Jiang , J. Ren , et al., “Nitric Oxide‐Assisted Photodynamic Therapy for Enhanced Penetration and Hypoxic Bacterial Biofilm Elimination,” Advanced Healthcare Materials 12, no. 29 (2023): 2302031, 10.1002/adhm.202302031. 37515529

[112] J. Li , X. Liu , C. Wang , et al., “Bio‐Heterointerface Charging through Ultrasound‐Boosted Extracellular and Intracellular Electron Transfer for Rapid Bacterial Killing,” Materials Science and Engineering: R: Reports 161 (2024): 100861, 10.1016/j.mser.2024.100861.

[113] Y. Wang , W. Guo , K. Zhang , et al., “Biomimetic Electrodynamic Metal‐Organic Framework Nanosponges for Augmented Treatment of Biofilm Infections,” Advanced Science 11, no. 46 (2024): 2408442, 10.1002/advs.202408442. PMC11633466 39422163

[114] X. Zhao , J. Li , P. Zhang , C. Xiao , and L. Chen , “Acid‐Responsive Bacteria‐Targeted Zinc‐Porphyrin Based Sonosensitizer with Enhancing Antibacterial Efficacy and Biofilm Eradication for Infected Wounds Healing,” Advanced Healthcare Materials 14, no. 9 (2025): 2404643, 10.1002/adhm.202404643. 39930718

[115] J. Li , Z. Chu , S. Wang , H. Liu , and B. Dong , “Study on Synergistic Mechanism of Cu‐TCPP@MnO_2_ Composites for Photothermal/Photodynamic/Chemodynamic Treatment of Infected Wound Healing,” Chemical Engineering Journal 512 (2025): 162334, 10.1016/j.cej.2025.162334.

[116] Y. Yu , Y. Zeng , Q. Ouyang , et al., “Ultrasound‐Induced Abiotic and Biotic Interfacial Electron Transfer for Efficient Treatment of Bacterial Infection,” ACS Nano 17, no. 21 (2023): 21018–21029, 10.1021/acsnano.3c03858. 37899553

[117] W. Nie , Y. Huang , Y. Wang , et al., “Temperature Sensitive PolyMOF Hydrogel Formed by in Situ Open‐Ring Polymerization for Infected Chronic Wound Treatment,” Chemical Engineering Journal 446 (2022): 136948, 10.1016/j.cej.2022.136948.

[118] J. Han , G. Liu , Y. Hou , et al., “Fabrication of Novel Porous Nano‐Pesticides by Modifying MPN onto Cu‐TCPP MOFs to Enhance Bactericidal Efficacy and Modulate Its Bioavailability,” Nano Letters 24, no. 45 (2024): 14405–14411, 10.1021/acs.nanolett.4c04277. 39470152

[119] Z. Zhu , L. Jiang , J. Cheng , et al., “Iridium‐Porphyrin MOF Nanospray: Dual‐Engined Photodynamic Antibacterial and Redox Homeostasis for Diabetic Wound Healing,” Chemical Engineering Journal 522 (2025): 167361, 10.1016/j.cej.2025.167361.

[120] H. Song , M. Zhang , Z. Qian , Z. Mao , and W. Tong , “A Mn‐incorporated Porphyrinic Metal‐organic Framework with Near‐Infrared Light Responsive Photothermal Effect and Intrinsic ROS Scavenging Ability for Promoted Healing of Bacteria‐Infected Wounds,” Colloids and Surfaces B: Biointerfaces 261 (2026): 115460, 10.1016/j.colsurfb.2026.115460. 41570415

[121] D. Gao , F. Wang , C. Zheng , B. Lv , and J. Ma , “A Durable Ag@MOF‐545/QCM‐Cotton Fabric with “Intelligent Bacteria‐Capturing and Dual Antibacterial” Properties,” ACS Applied Materials & Interfaces 17, no. 4 (2025): 6134–6143, 10.1021/acsami.4c21171. 39813137

[122] Z. Zhao , M. Luo , L. Li , and Z. Xiao , “Curcumin‐modified Mn‐based Metal–Organic Framework for Synergistic Sonodynamic and Cascade Anti‐Oxidation Therapy of Bacterial Osteomyelitis,” Colloids and Surfaces B: Biointerfaces 262 (2026): 115530, 10.1016/j.colsurfb.2026.115530. 41655336

[123] Q. Kong , M. Qi , W. Li , et al., “A Novel Z‐Scheme Heterostructured Bi_2_S_3_/Cu‐TCPP Nanocomposite with Synergistically Enhanced Therapeutics against Bacterial Biofilm Infections in Periodontitis,” Small 19, no. 43 (2023): 2302547, 10.1002/smll.202302547. 37376834

[124] J. Hynek , J. Zelenka , J. Rathouský , et al., “Designing Porphyrinic Covalent Organic Frameworks for the Photodynamic Inactivation of Bacteria,” ACS Applied Materials & Interfaces 10, no. 10 (2018): 8527–8535, 10.1021/acsami.7b19835. 29470048

[125] F.‐L. Meng , H.‐L. Qian , and X.‐P. Yan , “Conjugation‐Regulating Synthesis of High Photosensitizing Activity Porphyrin‐Based Covalent Organic Frameworks for Photodynamic Inactivation of Bacteria,” Talanta 233 (2021): 122536, 10.1016/j.talanta.2021.122536. 34215039

[126] J.‐X. Guo , X.‐M. Gao , T.‐Y. Gu , et al., “Porphyrin–Anthracene Covalent Organic Frameworks for Sustainable Photosterilization,” Journal of Materials Chemistry B 11, no. 46 (2023): 11094–11102, 10.1039/d3tb02017g. 37987617

[127] S. Wang , J. Zhang , L. Chu , et al., “Crown‐Ether Threaded Covalent Organic Polyrotaxane Framework (COPF) towards Synergistic Crown/Zn^2+^/Photothermal/Photodynamic Antibacterial and Infected Wound Healing Therapy,” Biomaterials Advances 159 (2024): 213814, 10.1016/j.bioadv.2024.213814. 38417206

[128] J. Li , H. Jin , T. Qin , F. Liu , S. Wu , and L. Feng , “Symmetrical Localized Built‐in Electric Field by Induced Polarization Effect in Ionic Covalent Organic Frameworks for Selective Imaging and Killing Bacteria,” ACS Nano 18, no. 5 (2024): 4539–4550, 10.1021/acsnano.3c11628. 38261792

[129] Y. Liu , Y. Li , L. Jiao , et al., “Hypoxia‐Activated Biodegradable Porphyrin‐Based Covalent Organic Frameworks for Photodynamic and Photothermal Therapy of Wound Infection,” ACS Applied Materials & Interfaces 16, no. 48 (2024): 65907–65917, 10.1021/acsami.4c14909. 39557630

[130] S.‐S. Zhao , X.‐P. Guo , X.‐H. Pan , Y.‐B. Huang , and R. Cao , “An “All in One” Strategy to Boost Antibacterial Phototherapy via Porphyrin and Boron Dipyrromethenes Based Covalent Organic Framework,” Chemical Engineering Journal 457 (2023): 141017, 10.1016/j.cej.2022.141017.

[131] Q. Yue , J. Yu , Q. Zhu , et al., “Polyrotaxanated Covalent Organic Frameworks Based on β‐Cyclodextrin towards High‐Efficiency Synergistic Inactivation of Bacterial Pathogens,” Chemical Engineering Journal 486 (2024): 150345, 10.1016/j.cej.2024.150345.

[132] B. Sun , Z. Ye , M. Zhang , et al., “Light‐Activated Biodegradable Covalent Organic Framework‐Integrated Heterojunction for Photodynamic, Photothermal, and Gaseous Therapy of Chronic Wound Infection,” ACS Applied Materials & Interfaces 13, no. 36 (2021): 42396–42410, 10.1021/acsami.1c10031. 34472332

[133] L.‐G. Ding , S. Wang , B.‐J. Yao , et al., “Synergistic Antibacterial and Anti‐Inflammatory Effects of a Drug‐Loaded Self‐Standing Porphyrin‐COF Membrane for Efficient Skin Wound Healing,” Advanced Healthcare Materials 10, no. 8 (2021): 2001821, 10.1002/adhm.202001821. 33433952

[134] W. Ji , P. Zhang , G. Feng , et al., “Synthesis of a Covalent Organic Framework with Hetero‐Environmental Pores and Its Medicine Co‐Delivery Application,” Nature Communications 14, no. 1 (2023): 6049, 10.1038/s41467-023-41622-x. PMC10539374 37770448

[135] Q. Fu , T. Zhang , M. An , et al., “Anchoring of Silver Nanoparticles by a Zinc‐Porphyrin Covalent Organic Framework for Synergistic Photodynamic Sterilization,” Chemical Engineering Journal 492 (2024): 152293, 10.1016/j.cej.2024.152293.

[136] L.‐G. Ding , S. Wang , B.‐J. Yao , et al., “Covalent Organic Framework Based Multifunctional Self‐Sanitizing Face Masks,” Journal of Materials Chemistry A 10, no. 7 (2022): 3346–3358, 10.1039/d1ta08743f.

[137] Y. Chen , T. Feng , X. Zhu , et al., “Ambient Synthesis of Porphyrin‐Based Fe‐Covalent Organic Frameworks for Efficient Infected Skin Wound Healing,” Biomacromolecules 25, no. 6 (2024): 3671–3684, 10.1021/acs.biomac.4c00261. 38720431

[138] Q. Fu , H. Li , M. An , et al., “Dialdehyde Cellulose Films Covalently Crosslinked with Porphyrin‐Based Covalent Organic Polymers for Photodynamic Sterilization,” International Journal of Biological Macromolecules 272 (2024): 132893, 10.1016/j.ijbiomac.2024.132893. 38838883

[139] M.‐C. Wang , J.‐X. Guo , L.‐J. Chen , and X. Zhao , “Acrylate‐Functionalized Porphyrin‐Covalent Organic Framework for Bacterial‐Targeted and Reaction‐Enhanced Synergistic Phototherapy/Chemotherapy toward Sterilization and Wound Healing,” Biomaterials Science 11, no. 5 (2023): 1776–1784, 10.1039/d2bm01723g. 36648453

[140] J. Wu , T. Meng , X. Zhang , et al., “Glucose‐Responsive Zn(II)‐Porphyrin COF Adhesive Hydrogels with Dual‐Active Sites and GOx‐like Activity for Accelerated Wound Healing,” Advanced Healthcare Materials 14, no. 6 (2025): 2404076, 10.1002/adhm.202404076. 39743951

[141] H. Zhao , W. Zhou , S. Cao , R. Guo , L. Zhou , and L. Qiu , “Dual‐Enzymatic Nanocatalyst for Optimizing Hypoxia Wound Healing via Linkage‐Enhanced Sonodynamic Antimicrobial Therapy,” Small 21, no. 33 (2025): 2504524, 10.1002/smll.202504524. 40545835

[142] J.‐J. Qian , J.‐X. Guo , M.‐C. Wang , L.‐J. Chen , X. Zhao , and X.‐P. Yan , “Cationic Porphyrin‐Based Covalent Organic Frameworks for Enhanced Phototherapy and Targeted Chemotherapy of Bacterial Infections,” Journal of Colloid and Interface Science 692 (2025): 137494, 10.1016/j.jcis.2025.137494. 40187135

[143] T. Wu , F. Han , J. Mei , et al., “Photoactive Metal‐Covalent Organic Framework Nanozymes with Enhanced Peroxidase‐Mimicking Activity for Eliminating Drug‐Resistant Bacterial Infections,” Journal of Colloid and Interface Science 699 (2025): 138178, 10.1016/j.jcis.2025.138178. 40527142

[144] D. Wei , W. Shi , Y. Zhao , et al., “Spatial Distortion‐Engineered Cationic Ru‐Covalent Organic Framework Overcoming Aggregation‐Caused Quenching for Synergistic Photodynamic/Photothermal Anti‐Infective Therapy,” ACS Applied Materials & Interfaces 17, no. 41 (2025): 56786–56801, 10.1021/acsami.5c13840. 40925878

[145] Y. Hu , L. Xiao , M. Zang , et al., “Self‐Amplifying Metal–Covalent Organic Framework for Programmable Antibacterial‐Regenerative Therapy via ROS Cascades,” ACS Applied Polymer Materials 7, no. 20 (2025): 13566–13585, 10.1021/acsapm.5c02395.

[146] W. Yao , T. Wang , W. Sun , et al., “Mature Biofilm‐Sensitive Lysozyme‐Grafted Bi‐Guanidine Backbone Porphyrin Nanorods for Deep Penetration and Double Phototherapy,” Biomaterials 323 (2025): 123431, 10.1016/j.biomaterials.2025.123431. 40435816

[147] X. Tao , Z.‐J. Fang , R. Shao , et al., “Covalent Organic Framework Nanohydrogel‐Based Oxidase‐Mimicking Nanozyme for Photocatalytic Antibacterial Therapy,” Angewandte Chemie International Edition 64, no. 41 (2025): 202513754, 10.1002/anie.202513754. 40827683

[148] J. Zhang , J. Wang , Y. Zhao , et al., “π‐Stacking‐Resistant Cationic Metalloporphyrin COF with Dual‐Enzyme Mimetics for Infected Wound Therapy,” ACS Applied Polymer Materials 7, no. 21 (2025): 14082–14095, 10.1021/acsapm.5c01959.

[149] J.‐J. Liu , J. Wang , Q.‐Y. Chen , F. Chen , and G.‐J. Wang , “Photoresponsive Nanoarchitectonics Based on Copper‐Porphyrins for Near‐Infrared‐Enhanced Bacterial Treatment,” ACS Omega 9, no. 23 (2024): 24513–24519, 10.1021/acsomega.4c00496. PMC11170729 38882152

[150] G. Zhang , G. Chen , M. Dong , J. Nie , and G. Ma , “Multifunctional Bacterial Cellulose/Covalent Organic Framework Composite Membranes with Antifouling and Antibacterial Properties for Dye Separation,” ACS Applied Materials & Interfaces 15, no. 27 (2023): 32903–32915, 10.1021/acsami.3c05074. 37377346

[151] Y.‐J. Hou , S. Fang , X.‐Y. Zhang , et al., “Tetrazolyl Porphyrin‐Based Hydrogen‐Bonded Organic Frameworks: Active Sites‐Mediated Host‐Guest Synergy for Advanced Antimicrobial Applications,” ACS Applied Materials & Interfaces 14, no. 44 (2022): 49875–49885, 10.1021/acsami.2c15869. 36288457

[152] B.‐T. Liu , X.‐H. Pan , D.‐Y. Nie , X.‐J. Hu , E.‐P. Liu , and T.‐F. Liu , “Ionic Hydrogen‐Bonded Organic Frameworks for Ion‐Responsive Antimicrobial Membranes,” Advanced Materials 32, no. 48 (2020): 2005912, 10.1002/adma.202005912. 33124716

[153] L. Wu , X. Yang , H. Jia , et al., “Freestanding Hydrogen‐Bonded Organic Framework Membrane for Efficient Wound Healing,” Advanced Materials 36, no. 47 (2024): 2411229, 10.1002/adma.202411229. 39363671

[154] J. Xiao , A. R. M. Shaheer , C. Liu , T.‐F. Liu , and R. Cao , “Hydrogen‐Bonded Organic Framework Core–shell Composite for Synergistic Antimicrobial Therapy,” Aggregate 5, no. 2 (2024): 481, 10.1002/agt2.481.

[155] Q. Yin , P. Zhao , R.‐J. Sa , et al., “An Ultra‐Robust and Crystalline Redeemable Hydrogen‐Bonded Organic Framework for Synergistic Chemo‐Photodynamic Therapy,” Angewandte Chemie International Edition 57, no. 26 (2018): 7691–7696, 10.1002/anie.201800354. 29696754

[156] J. Tang , J. Liu , Q. Zheng , et al., “In‐Situ Encapsulation of Protein into Nanoscale Hydrogen‐Bonded Organic Frameworks for Intracellular Biocatalysis,” Angewandte Chemie International Edition 60, no. 41 (2021): 22315–22321, 10.1002/anie.202105634. 34382314

[157] Y. Lin , X. Jiang , S. T. Kim , et al., “An Elastic Hydrogen‐Bonded Cross‐Linked Organic Framework for Effective Iodine Capture in Water,” Journal of the American Chemical Society 139, no. 21 (2017): 7172–7175, 10.1021/jacs.7b03204. 28506061

[158] Y. Hou , X.‐S. Huang , S.‐H. Gong , C. Liu , Y. Liu , and T.‐F. Liu , “Stable Hydrogen‐Bonded Organic Frameworks and Their Photo‐ and Electro‐Responses,” Nano Research 17, no. 8 (2024): 7675–7699, 10.1007/s12274-024-6665-7.

[159] J. Xu , H. Zhang , X. Ren , et al., “A Continuous Porous Porphyrinic Polymer Thin‐Film Composite Membrane for Anti‐Biofouling and Molecular Sieving,” Journal of Materials Chemistry A 12, no. 38 (2024): 26170–26177, 10.1039/d4ta04510f.

[160] D. Xu , S. Cao , W. Bian , et al., “Triple‐Enzyme Mimetic Flexible‐Interlaced Ferromagnetic Porous‐Organic‐Polymer for Self‐Adaptive Antibacterial Cascade Therapy via Microenvironment‐Reprogramming and Photosensitizer‐Aggregation‐Suppression,” ACS Applied Polymer Materials 7, no. 15 (2025): 9617–9633, 10.1021/acsapm.5c00977.

[161] Z. Zhang , L. Shi , L. Chu , et al., “Crown Ether‐Based Porous Organic Polymer Encapsulated Ag_2_[Fe(CN)_5_NO] Composite towards Ultra‐Low Dose Efficient Sterilization and Wound Healing Application,” Materials Today Chemistry 34 (2023): 101794, 10.1016/j.mtchem.2023.101794.

[162] Y. Wang , F. Chen , Q. Chen , et al., “Bioinspired Photothermal Zwitterionic Fibrous Membrane for High‐Efficiency Solar Desalination and Electricity Generation,” Nature Communications 16, no. 1 (2025): 6373, 10.1038/s41467-025-61244-9. PMC12246431 40640127

[163] Z. Li , X. Li , and Y.‐W. Yang , “Conjugated Macrocycle Polymer Nanoparticles with Alternating Pillarenes and Porphyrins as Struts and Cyclic Nodes,” Small 15, no. 12 (2019): 1805509, 10.1002/smll.201805509. 30735309

[164] Q. Fu , X. Sun , T. Zhang , et al., “Porphyrin‐Based Covalent Organic Polymers with Customizable Photoresponses for Photodynamic Inactivation of Bacteria,” Science of the Total Environment 906 (2024): 167475, 10.1016/j.scitotenv.2023.167475. 37797764

[165] P. Chen , R. Ze , X. Xia , et al., “Composite Porphyrin‐Based Conjugated Microporous Polymer/Graphene Oxide Capable of Photo‐Triggered Combinational Antibacterial Therapy and Wound Healing,” Biomaterials Advances 154 (2023): 213662, 10.1016/j.bioadv.2023.213662. 37862813

[166] S. C. Santamarina , D. A. Heredia , A. M. Durantini , and E. N. Durantini , “Antimicrobial Photosensitizing Material Based on Conjugated Zn(II) Porphyrins,” Antibiotics 11, no. 1 (2022): 91, 10.3390/antibiotics11010091. PMC8773278 35052968

[167] Z. J. Cheng , H. B. Xu , H. Y. Chen , et al., “Porphyrins Functionalized Covalent Organic Frameworks for Enhanced Photodynamic and Photothermal Antibacterial,” Journal of Photochemistry and Photobiology A: Chemistry 466 (2025): 116386, 10.1016/j.jphotochem.2025.116386.

[168] Z.‐Q. Wang , X. Wang , C. Li , and Y.‐W. Yang , “Covalent Organic Frameworks for Antibacterial Applications,” Cell Reports Physical Science 5, no. 3 (2024): 101845, 10.1016/j.xcrp.2024.101845.

[169] X. Zhang , Y. Liu , Q. Guo , et al., “Light‐Responsive Au@Zn‐TCPP Nanozyme Functionalized with Cell‐Penetrating Peptide and Antisense Oligonucleotide for Sensing Living Bacteria and Synergistic Therapy of Diabetic Wounds,” Chemical Engineering Journal 488 (2024): 150945, 10.1016/j.cej.2024.150945.

[170] K. Lin , H. Xu , H. Liu , et al., “Bioprinted Dressing with Symbiotic Microbes for Oxygen Supply and Antibacterial Therapy for Enhanced Diabetic Wound Healing,” Journal of Controlled Release 392 (2026): 114653, 10.1016/j.jconrel.2026.114653. 41580129

[171] Y.‐Y. Liu , L.‐J. Chen , X. Zhao , and X.‐P. Yan , “Effect of Topology on Photodynamic Sterilization of Porphyrinic Metal‐Organic Frameworks,” Chemistry – A European Journal 27, no. 39 (2021): 10151–10159, 10.1002/chem.202100920. 33978976

[172] S. He , A. Ban , Y. Chen , et al., “Defect‐Engineering Al‐CA/TCPP via Competitive Coordination with Carvacrol for Enhanced Photodynamic Antibacterial Therapy,” Chinese Chemical Letters (2025): 112239, 10.1016/j.cclet.2025.112239.

[173] S. He , D. Chu , Z. Pang , et al., “Pt Single‐Atom‐Functionalized 2D Al‐TCPP MOF Nanosheets for Enhanced Photodynamic Antimicrobial Therapy,” Acta Physico‐Chimica Sinica 41, no. 5 (2025): 100046, 10.1016/j.actphy.2025.100046.

[174] Y. Chen , T. Wang , Z. Li , B. Li , and H. Lyu , “A Core‐shell Upconversion Nanoparticle@Copper‐Porphyrin Metal‐Organic Framework for Near‐Infrared Light‐Triggered Synergistic Antibacterial Treatment,” Colloids and Surfaces B: Biointerfaces 262 (2026): 115539, 10.1016/j.colsurfb.2026.115539. 41678902

[175] Z. Feng , S. Chen , K. Han , et al., “Light Responsive Chitosan Hydrogel Incorporated with PCN‐224@Berberine for Antibiotic Resistant Bacterial Infected Wound Management,” International Journal of Biological Macromolecules 333 (2025): 148945, 10.1016/j.ijbiomac.2025.148945. 41224056

[176] X. Huang , J. Wang , H. Wang , et al., “Silver–Catechol Dynamic Redox Chemistry Provides Hydrogel Dressings with Sustained Antioxidant and Antibacterial Activity for Chronic Wound Care,” ACS Nano 19, no. 24 (2025): 22270–22290, 10.1021/acsnano.5c04690. 40504008

[177] A. Wang , W. Wang , W. Huang , et al., “A Dual Cross‐Linked Pillar[5]Arene/Porphyrin‐Based Supramolecular Polysaccharide Hydrogel for Promoted Wound Healing by Combating Multidrug‐Resistant Bacteria and Biofilm Eradication,” International Journal of Biological Macromolecules 338 (2026): 149761, 10.1016/j.ijbiomac.2025.149761. 41418955

[178] J. Cheng , H. Zhou , Z. Yu , et al., “Visible Light Driven Photodynamic Hydrogel with pH/Electrophysiological Dual‐Sensing Capabilities for Real‐Time Infection Monitoring and Smart Therapy,” Chemical Engineering Journal 528 (2026): 172490, 10.1016/j.cej.2025.172490.

[179] A. Kumar , A. Mondal , M. E. Douglass , et al., “Nanoarchitectonics of Nitric Oxide Releasing Supramolecular Structures for Enhanced Antibacterial Efficacy under Visible Light Irradiation,” Journal of Colloid and Interface Science 640 (2023): 144–161, 10.1016/j.jcis.2023.02.083. PMC10081829 36842420

[180] Y. Li , L. Wang , H. Liu , et al., “Ionic Covalent‐Organic Framework Nanozyme as Effective Cascade Catalyst against Bacterial Wound Infection,” Small 17, no. 32 (2021): 2100756, 10.1002/smll.202100756. 34212509

[181] C. Miao , L. Chu , D. Guo , et al., “Ferrocene‐Based Porous Polymer as Advanced Environmental Remediation Material for Self‐Enhanced Synergetic Photothermal‐Enzymatic Antibacterial and Reversible Iodine Capture,” Journal of Environmental Chemical Engineering 11, no. 5 (2023): 110514, 10.1016/j.jece.2023.110514.

[182] J. Li , Y. Fu , L. Chu , L. Shi , J. Zhang , and H. Ju , “Alder‐Longo Method Derived Cationic Porphyrin‐Based Porous Polymer as Physical Antibacterial Agent for the Bacteria‐Infected Wound Synergy Therapy,” ChemistrySelect 9, no. 24 (2024): 202401541, 10.1002/slct.202401541.

[183] S. Liang , M.‐H. Li , M.‐L. Qi , et al., “Reactive Oxygen Species‐Responsive Pillararene‐Embedded Covalent Organic Frameworks with Amplified Antimicrobial Photodynamic Therapy for the Targeted Elimination of Periodontitis Pathogens,” Nano Letters 24, no. 43 (2024): 13708–13717, 10.1021/acs.nanolett.4c03788. 39417607

[184] K. Su , Y. Yan , J. Huang , et al., “Mitochondrial‐Targeted Injectable Hydrogel for Periodontitis Therapy via Oral Immunity and Flora Regulation,” Bioactive Materials 58 (2026): 348–369, 10.1016/j.bioactmat.2025.12.002. PMC12765346 41492366

[185] J. Li , S. Song , J. Meng , et al., “2D MOF Periodontitis Photodynamic Ion Therapy,” Journal of the American Chemical Society 143, no. 37 (2021): 15427–15439, 10.1021/jacs.1c07875. 34516125

[186] S. Liang , M.‐H. Li , L. Cheng , et al., “H_2_S‐Amplified “Three‐in‐One” Antibacterial Strategy for Periodontitis Treatment Using a Photosensitive Pillararene‐Embedded COF/MOF Hybrid,” Advanced Science 13, no. 43 (2026): 75608, 10.1002/advs.75608. PMC13335815 42095449

[187] J. Zhang , Y. Sun , W. Yao , et al., “Self‐Regulated ROS Nanoplatform Enables Sequential Antibacterial Therapy and Inflammation Resolution in Periodontitis,” ACS Omega 11, no. 23 (2026): 34725–34737, 10.1021/acsomega.6c04003. PMC13557269 42719854

[188] Q. Liu , C. Tang , J. Liu , Y. Gao , and L. Peng , “Zr‐Porphyrinic MOF PCN‐224 Enables Sequential Antibacterial and Inflammation‐Modulating PDT for Periodontitis,” Colloids and Surfaces B: Biointerfaces 265 (2026): 115692, 10.1016/j.colsurfb.2026.115692. 41980357

[189] P. Ananthi , R. V. Naveen , K. Hemkumar , A. Praveen , and A. Pius , “Fluorescence Resonance Energy Transfer in Gelatin‐Based MOF/N‐CDs Films for Superior Photodynamic Antibacterial Packaging Materials,” ACS Sustainable Chemistry & Engineering 13, no. 8 (2025): 3052–3065, 10.1021/acssuschemeng.4c06496.

[190] B. Duan , Y. Yang , J. Tian , et al., “A Multitasking Metal‐Covalent Organic Framework for Synergistic Bacterial Elimination and Label‐Free Detection,” Chemical Engineering Journal 537 (2026): 176086, 10.1016/j.cej.2026.176086.

[191] Y. Pang , S. Wen , L. Shi , et al., “Intelligent and Sustainable Food Packaging Utilizing Quaternary Ammonium Chitosan with Porphyrin Structure for Extended Antibacterial Drug Release,” Food Chemistry 504 (2026): 148029, 10.1016/j.foodchem.2026.148029. 41558327

[192] C. Han , S. Zhang , W. Jiang , J. Cao , and G. Chen , “Polyvinyl Alcohol/Silk Fibroin‐Based Photodynamic Antimicrobial Composite Film Incorporated with CuBTC‐PDA‐TCPP for Fresh Pork Preservation,” Food Chemistry 495 (2025): 146417, 10.1016/j.foodchem.2025.146417. 41192240

[193] H. Wang , T. Shi , J. Ma , et al., “Chitosan‐Based Nanocomposite Films Incorporated with AgNPs/Porphyrinic MOFs for Killing Pathogenic Bacteria,” International Journal of Biological Macromolecules 295 (2025): 139584, 10.1016/j.ijbiomac.2025.139584. 39788251

[194] S. M. Sajjadinezhad , K. Tanner , and P. D. Harvey , “Metal‐Porphyrinic Framework Nanotechnologies in Modern Agricultural Management,” Journal of Materials Chemistry B 10, no. 44 (2022): 9054–9080, 10.1039/d2tb01516a. 36321474

[195] J. Tang , G. Ding , J. Niu , et al., “Preparation and Characterization of Tebuconazole Metal‐organic Framework‐based Microcapsules with Dual‐microbicidal Activity,” Chemical Engineering Journal 359 (2019): 225–232, 10.1016/j.cej.2018.11.147.

[196] H. Li , Y. Gao , X. Ding , et al., “Magnesium‐Doped Zr‐Porphyrin MOF Nanoarchitecture with pH/Photo Dual Stimuli‐Responsiveness for Targeted Pesticides and Improved Biosafety,” Journal of Nanobiotechnology 24, no. 1 (2025): 32, 10.1186/s12951-025-03899-6. PMC12797581 41354807

[197] X. Xiong , J. Li , M. Zhen , et al., “Supercritical CO_2_‐Assisted Synthesis of Porphyrin‐Based Covalent Organic Framework and Construction of Photodynamic Antibacterial Textiles,” Journal of Environmental Chemical Engineering 14, no. 3 (2026): 123026, 10.1016/j.jece.2026.123026.

[198] W.‐X. Wu , F. Li , B.‐J. Yao , et al., “Synthesis of Covalent Organic Frameworks via Kabachnik‐Fields Reaction for Water Treatment,” Journal of Hazardous Materials 433 (2022): 128831, 10.1016/j.jhazmat.2022.128831. 35417807

[199] K. S. Ndlovu , M. J. Moloto , K. E. Sekhosana , T. T. I. Nkambule , and M. Managa , “Porphyrins Developed for Photoinactivation of Microbes in Wastewater,” Environmental Science and Pollution Research 30, no. 5 (2023): 11210–11225, 10.1007/s11356-022-24644-8. 36515881

[200] Q. Wu , J. Liu , H. Wang , H. Wen , T. Sun , and Z. Xie , “Carrier‐Free Porphyrin‐Based Supramolecular Complexes for Near‐Infrared Photothermal‐Enhanced Wound Healing,” Chemical Engineering Journal 502 (2024): 158128, 10.1016/j.cej.2024.158128.

